# Design and synthesis of new energy restriction mimetic agents: Potent anti-tumor activities of hybrid motifs of aminothiazoles and coumarins

**DOI:** 10.1038/s41598-020-59685-x

**Published:** 2020-02-19

**Authors:** Fatema Hersi, Hany A. Omar, Raed A. Al-Qawasmeh, Zainab Ahmad, Areej M. Jaber, Dana M. Zaher, Taleb H. Al-Tel

**Affiliations:** 1 Sharjah Institute for Medical Research, University of Sharjah, Sharjah, 27272 United Arab Emirates; 2 College of Medicine, University of Sharjah, Sharjah, 27272 United Arab Emirates; 3 College of Pharmacy, University of Sharjah, Sharjah, 27272 United Arab Emirates; 4 Department of Pharmacology, Faculty of Pharmacy, Beni-Suef University, Beni-Suef, 62514 Egypt; 5 Department of Chemistry, Faculty of Science, University of Sharjah, Sharjah, 27272 United Arab Emirates; 6 Department of Chemistry, Faculty of Science, The University of Jordan, Amman, 11942 Jordan

**Keywords:** Biochemistry, Cancer screening, Cancer therapy, Organic chemistry, Medicinal chemistry, Drug discovery and development

## Abstract

The incidence of obesity-related diseases like diabetes, cardiovascular diseases, and different types of cancers shed light on the importance of dietary control as preventive and treatment measures. However, long-term dietary control is challenging to achieve in most individuals. The use of energy restriction mimetic agents (ERMAs) as an alternative approach to affect the energy machinery of cancer cells has emerged as a promising approach for cancer therapy. ERMAs limit the high need for energy in rapidly growing tumor cells, with their survival rate strongly dependent on the robust availability of energy. In this context, initial phenotypic screening of an in-house pilot compound library identified a new class of aminothiazole anchored on coumarin scaffold as potent anticancer lead drug candidates with potential activity as ERMA. The identified chemotypes were able to inhibit glucose uptake and increase ROS content in cancer cells. Compounds **9b**, **9c**, **9i**, **11b**, and **11c** were highly active against colorectal cancer cell lines, HCT116 and HT-29, with half-maximal inhibitory concertation (IC_50_) range from 0.25 to 0.38 µM. Further biological evaluations of **9b** and **9f** using Western blotting, caspase activity, glucose uptake, ROS production, and NADPH/NADP levels revealed the ability of these lead drug candidates to induce cancer cell death *via*, at least in part, energy restriction. Moreover, the assessment of **9b** and **9f** synergistic activity with cisplatin showed promising outcomes. The current work highlights the significant potential of the lead compounds, **9b**, and **9f** as potential anticancer agents *via* targeting the cellular energy machinery in cancer cells.

## Introduction

Many epidemiological studies indicated that obesity and excess body fat increase the risk of carcinogenesis^1,2^. In the last decade, the observed global rise in the body-mass index has led to an increase in the incidence of cancer-linked obesity^2,3^. The direct link to obesity makes colorectal cancer (CRC), the fourth leading cause of cancer morbidities and the second leading cause of cancer mortality worldwide^4,5^.

The conventional chemotherapeutic protocols such as FOLFIRI (Folinic acid, fluorouracil, and irinotecan) and FOLFOX (Folinic acid, fluorouracil, and oxaliplatin) are the currently available options for the management of advanced CRC^6^. Targeted therapy with epidermal growth factor receptor (EGFR) or vascular endothelial growth factor (VEGF) inhibitors improved the overall survival of CRC patients. However, the heterogeneous nature of CRC and the toxicity of chemotherapeutics continued the urgent need for the development of new agents targeting CRC^7^. On the other hand, caloric restriction (CR) has acquired a great interest as a booster for longevity, cancer prevention, and many therapeutic approaches^8–10^. The increased interest in CR is a consequence of the metabolic shift of tumor cells towards the aerobic glycolysis and the upregulation of glucose consumption or what is known as the Warburg effect^11,12^. Therefore, many studies focused on targeting the metabolism in cancer cells by energy restriction mimetic agents (ERMAs) or via reprogramming the energy production in cancer cells^13,14^. ERMAs are compounds that target cellular metabolism and activate the cellular stress response^15–17^. In addition to the more straightforward implementation, the use of ERMAs has the advantage of mimicking most of the CR cellular responses without reducing food consumption or causing undesired side effects^8,18^.

**Figure 1:**
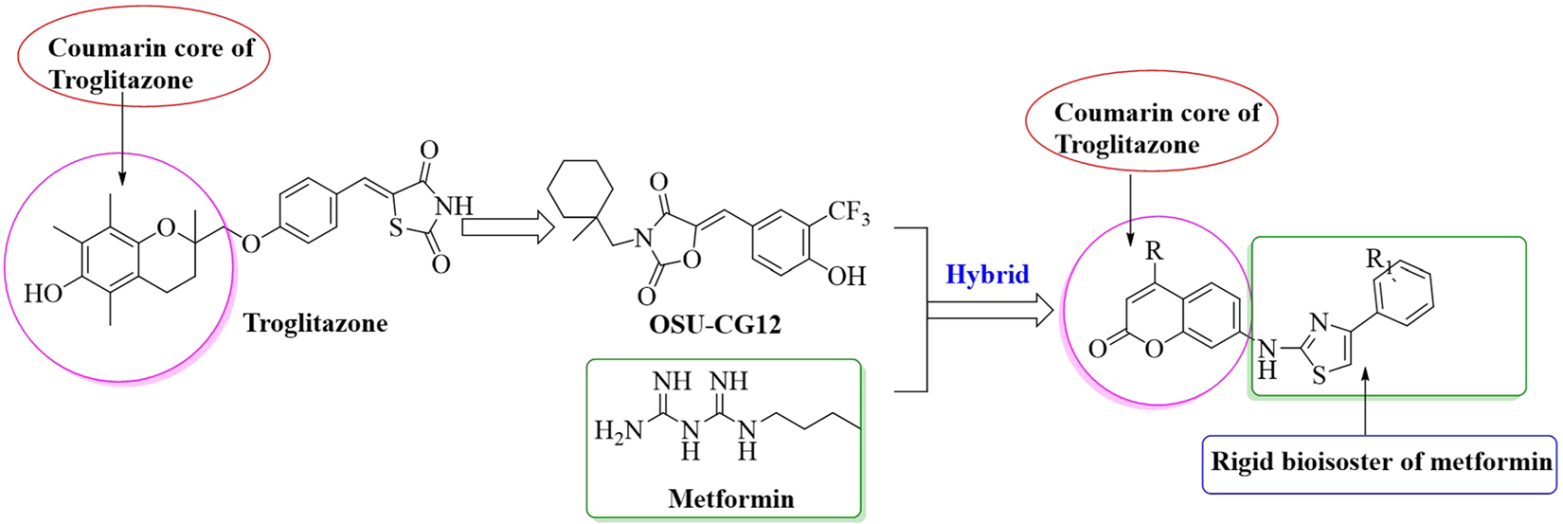
Analogue-based drug design of hybrid scaffolds encompassing coumarin and aminothiazoles.

In this study, the screening of a pilot compound library against an in-house panel of cancer cell lines identified new anticancer lead drug candidates. These molecules possess a hybrid structure that encompasses an aminothiazole moiety anchored on a coumarin core^19^. Coumarin system, represented by troglitazone analogues^13,19^, were reported as energy restriction mimetic agents. Furthermore, ERMAs such as metformin were also reported to deprive cells of energy^13,19,20^. Inspired by these reports, we envisioned that a hybrid structure encompassing an aminothiazole moiety, a rigid isostere of metformin, and coumarin system might lead to the discovery of potent anticancer lead drug candidates that deprive cellular energy. Henceforth, a pilot library of aminothiazoles anchored on the coumarin ring as potential ERMA was synthesized (Fig. 1). One of our key objectives is to examine the newly designed compounds as potential anticancer lead drug candidates for the treatment of CRC. Besides, the combination of ERMAs with classical chemotherapeutic agents like cisplatin can exploit the maximal benefit through synergistic mechanisms. Targeting the survival signaling in CRC by ERMAs represents a therapeutically relevant approach for treatment, and ultimately might lead to new therapies that improve the treatment and increase the survival of CRC patients.

## Results and Discussion

### Initial screening

**Scheme 1:**
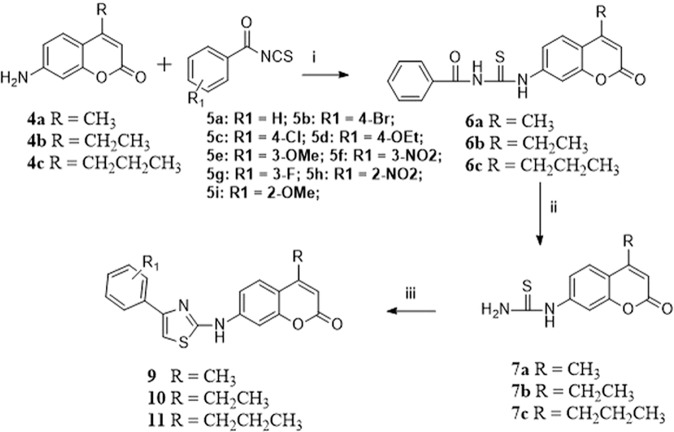
Preparation of the key intermediates **4a–c**. Reagents and conditions: (**i**) methoxycarbonyl chloride/KHCO_3(aq)_/EtOAc, RT, 4 h; (**ii**) β-ketoester/H_2_SO_4_, 10–15 ^o^C, RT, 3 h; (**iii**) 45% KOH, 80–90 ^o^C, 3 h.

**Figure 2:**
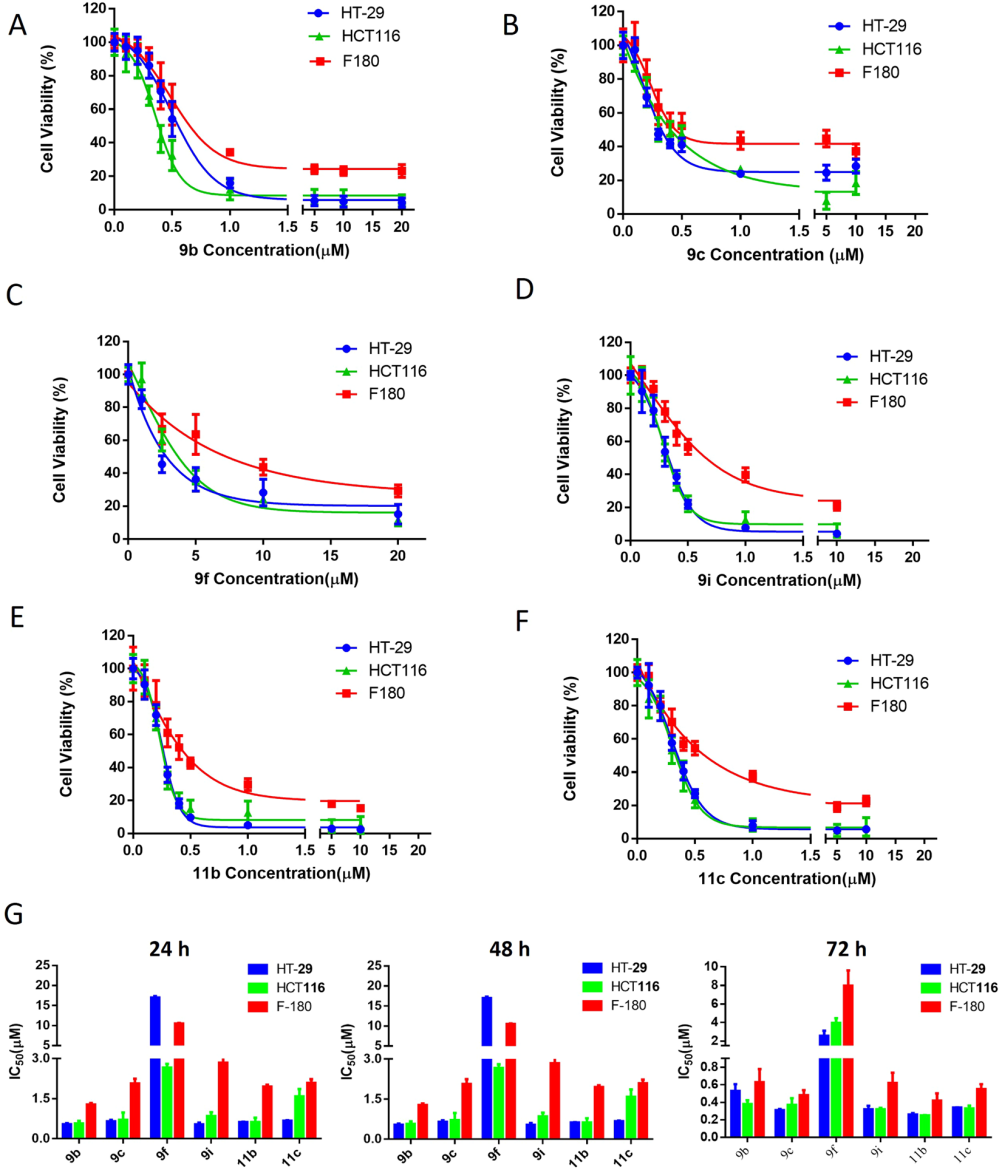
The antiproliferative activity of the highly potent compounds on different cell lines. (**A–F**) Dose-dependent effects of **9b**, **9c**, **9f**, **9i**, **11b**, and **11c** on the cell viability of colorectal cancer cell lines and F180 cells after 72 h treatment. Points, mean; bars, SD (n = 6). (**G**) The IC_50_ of selected compounds in normal fibroblasts (F180) and CRC cells after 24 h, 48 h, and 72 h treatments. Data are mean ± SD, n = 3.

In the search for Energy Restriction Mimetic Agents (ERMAs), we have initially screened an in-house pilot library of more than fifty compounds with diverse scaffolds against a panel of cancer cell lines. Such a phenotypic screen identified an anticancer compound that possesses thiourea derivative anchored on a coumarin scaffold. Thus, a systematic and biased structure-activity relationship (SAR) study was followed, and a small set of compounds was first assembled. Then, screening was performed for their anticancer activities against HCT116 and HT-29 cancer cell lines. The best hits were structurally assessed and subjected to subsequent SAR development. From these experiments and using a predictive physicochemical guideline of small molecules, we concluded that a hybrid structure that connects an aminothiazole to a coumarin moiety is essential for the anticancer activity. Such initiatives led to the development of potent motifs describes in Table 1. Specifically, we intended first to prepare a small library of various aminothiazoles anchored at the 7-position of coumarin through derivatization of the 7-amino coumarin of type **4**, as shown in Scheme 1. With the pilot library in hand, screening against cancer cell lines identified several potent anticancer probes, compounds **9b**, **9c**, **9i**, **11b**, and **11c**, with IC_50_ values of less than 0.6 µM (Table 1). The *in vitro* antiproliferative activities of the most potent compounds, as indicated by their IC_50_ values, are summarized in Fig. 2G.

**Table 1:** The half-maximal inhibitory concentration (IC_50_) values of the hybrid aminothiazole-coumarin synthesized compounds (72 h)^a^.

Compound	HT-29	HCT116
**9a**	1.54 ± 0.020	1.65 ± 307
**9b**	0.38 ± 0.044	0.53 ± 0.077
**9c**	0.37 ± 0.077	0.31 ± 0.014
**9d**	3.03 ± 0.0205	3.21 ± 0.88
**9e**	3.00 ± 0.159	3.05 ± 0.14
**9f**	3.96 ± 0.506	2.56 ± 0.553
**9g**	2.91 ± 1.25	3.5 ± 0.93
**9h**	>30	>30
**9i**	0.32 ± 0.017	0.32 ± 0.041
**10a**	>30	>30
**10b**	2.97 ± 0.20	3.03 ± 2.25
**10c**	1.3 ± 0.127	3.54 ± 0.53
**10e**	13.06 ± 1.39	>30
**10f**	4.41 ± 1.37	10.3 ± 3.15
**10g**	2.34 ± 0.615	4.08 ± 2.68
**10h**	1.68 ± 0.47	4.73 ± 1.93
**10i**	3.96 ± 1.65	13.49 ± 0.20
**11a**	2.13 ± 0.30	6.76 ± 0.28
**11b**	0.25 ± 0.004	0.26 ± 0.016
**11c**	0.33 ± 0.032	0.34 ± 0.0009
**11e**	8.89 ± 0.813	28.59 ± 6.558
**11f**	3.66 ± 0.038	>30
**11g**	1.80 ± 0.76	4.08 ± 1.36
**11h**	3.96 ± 0.014	6.3 ± 0.605
**4-Hydroxycoumarin**	>200	>200
**OSU-CG5**	10.14 ± 0.373	2.85 ± 0.111
**OSU-CG12**	10.82 ± 1.259	3.4 ± 0.581

^a^The half-maximal inhibitory concentration (IC50) values of all compounds, screened in CRC using the MTT assay. Values mean ± SD (n = 6).

### Synthesis and design

**Scheme 2:**
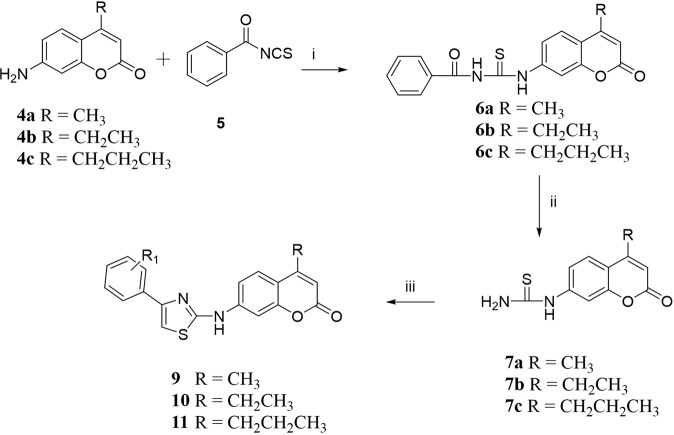
Synthesis of the target compounds **9**, **10** and **11**. Reagents and conditions: (**i**) Acetone/reflux 3–4 h; (**ii**) MeOH/1 N NaOH, reflux, 3–4 h; (**iii**) α-bromoacetophenone derivative/EtOH, reflux, 4 h.

The synthesis of the designed compounds is contemplated in Schemes 1 and 2. The key starting materials 7-Amino-4-substituted coumarin **4a–d** were obtained utilizing modified Pechmann reaction^21^ in which *m*-aminophenol **1** was protected with methoxycarbonyl chloride to afford the urethane **2**. The reaction of the latter with the appropriate β-ketoester ester using sulfuric acid produced the coumarins **3a–c**. Basic hydrolysis of the carbamates **3a–c** delivered the desired 7-amino-4-substituted coumarins **4a–c** in good yields^22^.

With the key building blocks **4a–c** in hand, it was envisaged that reactions with benzoyl isothiocyanate should deliver N-(4-Substituted-2-oxo-2H-chromen-7-ylcarbamothioyl) benzamide **6a–c**. The basic hydrolysis of the latter compounds produced the 7-thiourea coumarin conjugates **7a–c**. Construction of the aminothiazole scaffolds **9–11**, was achieved by reaction of the thiourea derivatives **7a–c**, with various α-bromoacetophenone derivatives **8a–i** in refluxing ethanol (Table 2). The characterization of the new compounds was carried out through ^1^H NMR, ^13^C NMR, and HRMS.

**Table 2:** structures of the synthesized hybrid aminothiazole-coumarin systems.


Code	R_1_								
	a	b	c	d	e	f	G	h	i
**9**	H	4-Br	4-Cl	4-OEt	3-OMe	3-NO_2_	3-F	2-NO_2_	2-OMe
**10**	H	4-Br	4-Cl	—	3-OMe	3-NO_2_	3-F	2-NO_2_	2-OMe
**11**	H	4-Br	4-Cl	—	3-OMe	3-NO_2_	3-F	2-NO_2_	—

Initial screening of the developed compounds against HT-29 and HCT116 cancer cell lines (Table 1 and Fig. 2) indicated that compounds **9a, 9b, 9c, 9f, 9i, 11b**, and **11c** exhibited significant anticancer activities. As a result, compounds possessing electron-withdrawing groups at the phenyl ring of the thiazole moiety are more potent than those with electron-releasing groups. Furthermore, when the coumarin ring carries a methyl group at the β-position of the cyclic ester (e.g. **9a, 9b, 9c, 9f**), the anticancer activities are higher than those with bulkier groups like ethyl or propyl (e.g. derivatives of scaffolds **10** and **11**, Scheme 2). This observation could also be further rationalized based on the structural similarity of our motifs and that of **OSU-CG12**^17,19^. To elaborate on this in brief: If our scaffolds possess a similar mechanism of action as that of **OSU-CG12** (Fig. 1), then an electron-withdrawing group on the phenyl ring of our motifs should mimic that of the phenyl ring in **OSU-CG12**. Therefore, compounds with electron-withdrawing groups (**9a**, **9b**, **9c**, and **9f**) are the most active as anticancer agents. Furthermore, the cyclohexyl group in **OSU-CG12** is not substituted. Therefore, bulkier groups on its analogue, the coumarin ring in our motifs, should not be tolerated and, therefore, less potent as anticancer.

## Biological Evaluation

### Cell viability of human colorectal cancer cell lines

The potential cytotoxicity of 24 candidate compounds was initially evaluated at a single concentration level (5 μM). Then a full dose-response curve was plotted for each compound against different colorectal cancer cell lines (HCT116 and HT-29) by MTT assay after 72 h treatments. The half-maximal inhibitory concertation (IC_50_) values after 72 h treatment are summarized in Table 1. 4-hydroxycoumarin, **OSU-CG5**, and **OSU-CG12** were used as positive controls. Most compounds exhibited higher potency in comparison to the positive controls except **9h** (>30 µM),**10a** (>30 µM) and **10e** (13.06 ± 1.39), in HT-29 and **9h** (>30 µM), **10a** (>30 µM), **10e** (>30 µM) **10f** (10.3 ± 3.15), **10i** (13.49 ± 0.20), **11a** (6.76 ± 0.28), **11e** (28.59 ± 6.56), and **11f** (>30 µM) in HCT116 cells. Compounds **9b**, **9c**, **9f**, **9i**, **11b**, and **11c** were selected for further studies to determine the full dose-response survival curves using different concentrations (0.1–20 µM) against CRC cell lines and normal fibroblast cell line (F180) at different time points (Fig. 2A–2F and supplementary material Page S63 and S64). The IC_50_ values of the new compounds are ranging from 0.25 µM to 3.96 µM at 24 h, 48 h, and 72 h (Table 1 and Fig. 2G). Moreover, to understand the structure-activity relationship (SAR) considering the potency and safety of the new compounds, the most potent compound **9b**, and the safest compound on normal cells (**9f**, IC_50_ = 7.97 µM), were selected for further biological evaluation and analysis.

### Activation of energy restriction cellular responses

**Figure 3:**
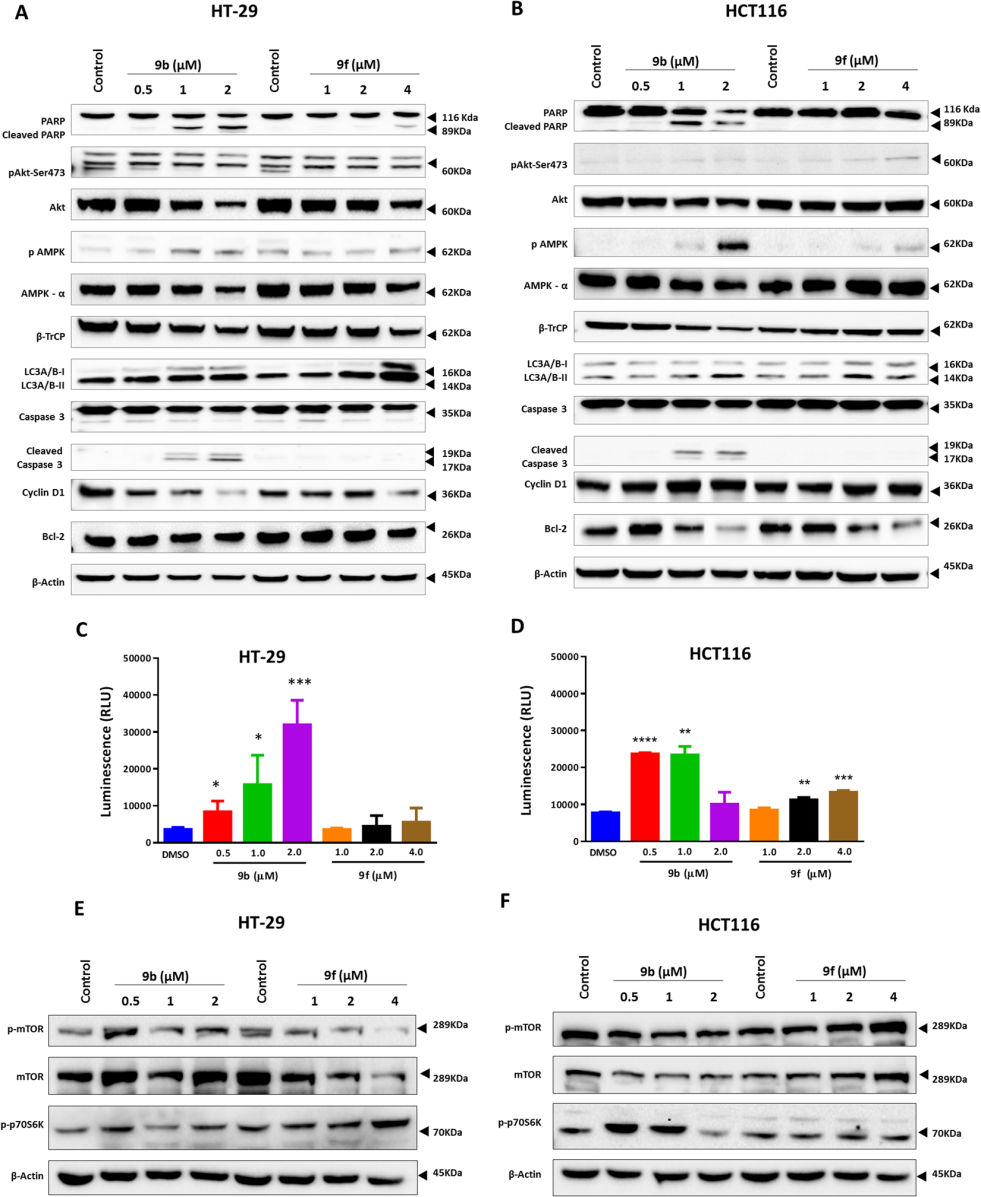
The anticancer activity of 9b and 9f is mediated through the activation of energy restriction cellular responses. (**A**,**B**) Western blot analysis of the expression levels of PARP, p-AKT, AKT p-AMPK, AMPK, β-TrCP, Cyclin D1, LC3A/B, Caspase-3, Cyclin D1, and Bcl-2 in HT-29 and HCT116 cells after 72 h of treatment with **9b** and **9f**. (**C**,**D**) Caspase 3/7 activities in HT-29 and HCT116 cells after treatment with **9b** and **9f** for 72 h. *indicates significant difference versus control at p < 0.05 (*), p < 0.01 (**), p < 0.001 (***), p < 0.0001 (****). All assays were performed in triplicates. Full-length blots are available in the supplementary material (Page S47-S62). (**E**,**F**) Western blot analysis of the expression levels of p-mTOR (Ser2448), mTOR and p-p70S6K (Thr389) in HT-29 and HCT116 cells after 72 h of treatment with **9b** and **9f**.

Many ERMAs were reported to induce energy restriction cellular responses, including AMPK phosphorylation, β-TrCP upregulation, downregulation of cyclin D1 and inhibition of Akt phosphorylation^13,23^. Based on this, the energy restriction mediated anticancer activity of the new coumarin tagged aminothiazole derivatives was investigated. The expression levels of PARP, p-Akt, Akt, p-AMPK, AMPK, β-TrCP, LC3A/B-I, LC3A/B-II, caspase-3, cyclin D1, and Bcl-2 proteins were determined by Western blot analysis after 72 h treatment of HT-29 and HCT116 at different concentrations based on the IC_50_ values of each compound (Fig. 3A,B). The glucose starvation and apoptosis proteins markers used in this study were found to be differentially expressed upon treatment with the selected compounds **9b** and **9f**. Moreover, the treatment with **9b** and **9f** induced caspase 3/7 activation, which was detected by caspase 3/7 activity assay and confirmed by Western blot. The activation of caspases was significant in **9b** in both cell lines and significant with **9f** in HCT116 cells only and minimal in HT-29 cells (Fig. 3C,D). On the other hand, both **9b** and **9f** induced dose-dependent proteolytic processing of PARP. **9b** induced PARP cleavage, a signature of apoptosis, in a dose-dependent manner in both tested cell lines. However, **9f** has shown PARP cleavage only in HT-29 cells at the highest tested dose. Besides, the protein expression level of the anti-apoptotic protein, Bcl-2, was reduced in HCT116 upon treatment with both compounds. These findings suggest the involvement of apoptosis as a mechanism of cell death (Fig. 3A,B).

The treatment with **9b** and **9f** resulted in the enhancement of AMPK activation through phosphorylation in both HT-29 and HCT116 cells. Besides, the inhibition of Akt phosphorylation was detected upon the treatment of HT-29 cells with **9b** and **9f**. However, Akt phosphorylation was not observed in HCT116 cells (Fig. 3A,B). This finding was in line with many studies in which this mutant PIK3CA cell line exhibited an absence of Akt phosphorylation^24,25^. Surprisingly, **9f** induced Akt phosphorylation at high concentrations in the HCT116 cell line only. The induction of Akt phosphorylation was previously demonstrated in different cells treated with the glucose inhibitor, 2-Deoxy-D-glucose (2-DG) and curcumin^26,27^.

Additionally, a dose-dependent downregulation of β-TrCP and cyclin D was observed in HT-29 cells on the treatment with both compounds, and the same effect was observed in HCT116 cells treated with **9b** (Fig. 3A,B). On the other hand, no significant changes in the expression of cyclin D and β-TrCP were noticed in HCT116 cells on other treatments. These findings are not in parallel to previous studies that showed the ability of ERMAs to induce β-TrCP-mediated proteolysis, leading to cell cycle arrest^13,19^. On the other hand, our results confirmed the clinical findings in colorectal cancer tissues, where β-TrCP is overexpressed and could possibly play a role as a tumor oncogene^28^. Also, it has been revealed that β-TrCP is controlled by the mammalian target of rapamycin (mTOR), the cellular metabolism regulator, with consequent downregulation of cyclin E in triple-negative breast cancer cells^29^.

Another anticancer mechanism of energy restriction is the induction of autophagy^30^. To explore the ability of the new compounds to induce autophagy, the expression levels of LC3-II (LC3A-II and LC3B-II), which is a central component of the autophagosome membrane, were assessed using Western blot^31^. The results indicated that treatment with compounds **9b** and **9f** induced the expression of LC3A-II and LC3B-II in CRC cell lines (Fig. 3A,B). These results provided further evidence that these new compounds could possibly activate autophagy *via* the AMPK-TSC1/2-mTOR signaling pathway. Therefore, we have analyzed the protein expression levels of p-mTOR (Ser2448), mTOR and p-p70S6K (Thr389) (Fig. 3E, F). Interestingly, we observed an initial increase in p-mTOR and p-p70S6K at 0.5 µM of **9b** in HT-29 and HCT116 cell lines. This increase could possibly serve as a protective mechanism to maintain cellular homeostasis. Earlier studies have also found an initial increase in mTOR due to the exposure to cellular stress such as radiation or treatment with hydrogen peroxide^32,33^. On the other hand, we have observed a decrease in p-mTOR and p-p70S6K levels at higher concentrations of **9b** and **9f** in both cell lines except at doses 1 and 2 µM in HCT116 cells, where induced p-mTOR was observed. In addition, decreased p-mTOR and p-p70S6K was parallel to an increase in p-AMPK level and a decrease in p-Akt in HT-29 cells. Furthermore, in HCT116 cells, which have mutant PIK3CA^24,25^, the observed increase in mTOR expression was not correlated to the increase in p-p70S6K and the upregulation of p-p70S6K was independent of mTOR, which requires further investigation. However, these results confirmed that the mechanism of anticancer activity of the new compounds was mediated, at least in part, through the activation of AMPK, which promotes the inhibition of mTOR^34^.

### Induction of cell cycle arrest at G1 phase in HT-29 cells upon treatment with 9b and 9f

**Figure 4:**
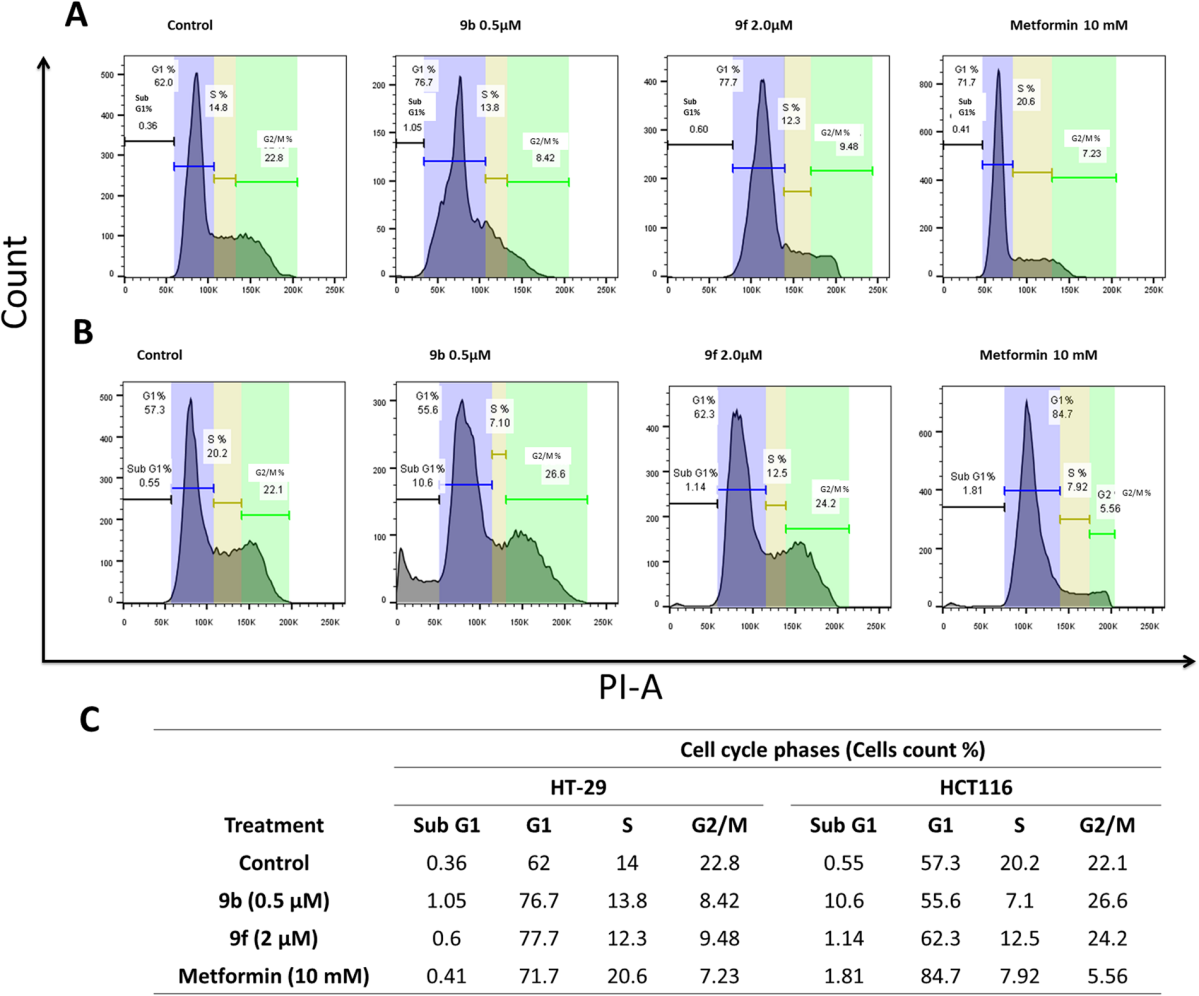
Cell cycle analysis of HT-29 and HCT116 treated with **9b**, **9f**, and metformin for 24 h. (**A**) Histogram representation of the cell cycle distributions of HT-29 treated with **9b** and **9f** at indicated treatments for 24 h. (**B**) Histogram representation of the cell cycle distributions of HCT-116 treated with **9b** and **9f** at the indicated treatments for 24 h. (**C**) Quantification of percentages of HT-29 and HCT116 cells in different sub-population phases in the histogram.

To further investigate the effect of the synthesized compounds on cell cycle progression, we examined cell cycle profile of cells upon 24 h treatment in comparison to metformin, a known AMPK activator that inhibits cell proliferation in CRC cells through p53-independent manner^35^. In HT-29 cells, we have observed the accumulation of cells in G1 and a decrease in the G2/M phase upon the treatment with **9b**, **9f**, and metformin (Fig. 4A, C). These changes in cell cycle were in correlation with the observed reduction in cyclin D1 expression in this p53-mutant cell line (Fig. 3A). These results could suggest that the induced cell cycle arrest by the candidate compounds is p53 independent. Whereas, in HCT116, a p53 wild type expressing cell line, **9b** treatment increased the number of cells in the sub-G1 cell population, which represents the dead cells. Besides, **9f** caused a slight increase in the G1 cell population (Fig. 4B,C). However, in Western blot, the expression of cyclin D1 was not reduced in HCT116 cells (Fig. 3B).

### Impact of 9b and 9f on glucose uptake and ROS production in CRC

**Figure 5:**
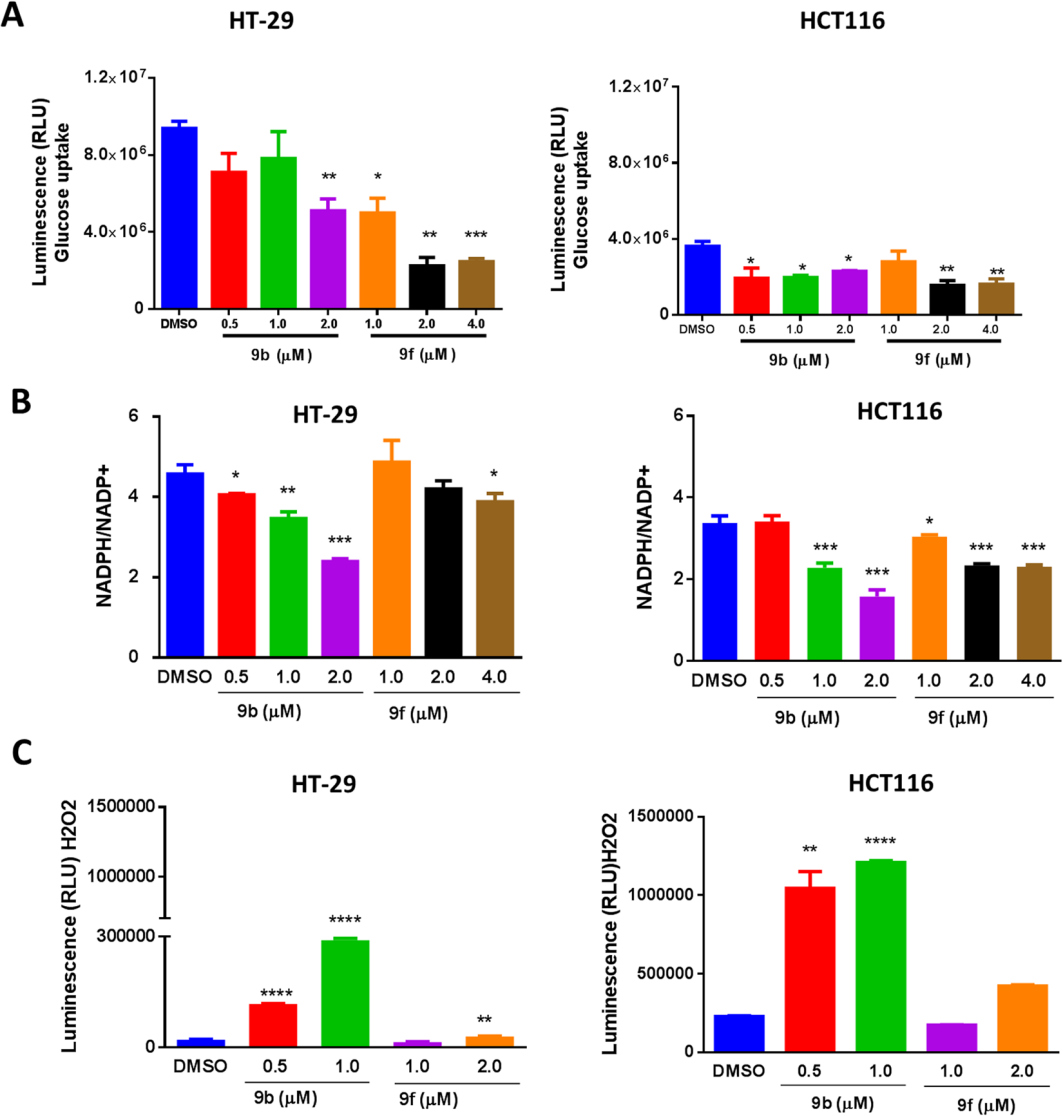
The impact of **9b** and **9f** on glucose uptake and ROS production in CRC. (**A**) Glucose uptake in HT-29 and HCT116 treated with **9b**, and **9f** measured as relative light units (**B**) Cells were analyzed for NADP+ and NADPH levels following treatment with **9b** and **9f**. The results are expressed as fold change of NADPH/NADP+. Data points are shown with error bars representing ± SEM. (**C**) H_2_O_2_ induction was measured as relative light units (RLU). Measured luminescence was converted to the concentration of H_2_O_2_ after background correction. Data points are shown with error bars representing ± SEM. *indicates significant difference versus control at P < 0.05 (*), P < 0.01 (**), P < 0.001 (***), P < 0.0001 (****). All assays were performed in triplicates.

According to previous studies, ERMAs inhibited glucose utilization in cancer cells^13,19^. Therefore, we investigated glucose uptake (Fig. 5A) upon treatment with **9b** and **9f** in HT-29 and HCT116 cells at different concentrations based on the IC_50_ of each compound. Glucose uptake was inhibited significantly in both cell lines in a dose-dependent manner in **9b** and **9f** after 16 h treatment. Further investigations of **9b** and **9f** activities have shown a significant decrease in NADPH/NADP + ratio (Fig. 5B). This effect is known to result in the accumulation of reactive oxygen species (ROS) and could play a role in glucose deprivation-induced cytotoxicity and oxidative stress in cancer cells^36^. Interestingly, **9b** and **9f** significantly increased the generation of hydrogen peroxide (H_2_O_2_) and ROS in HCT116, while only **9b** caused the same effect in HT-29 cells (Fig. 5C). The observed increase in ROS levels could lead to oxidative stress and extensive cell death in the absence of NADPH detoxification^36^.

### Anticancer activity of 9b and 9f in combination with cisplatin in CRC cells

**Figure 6:**
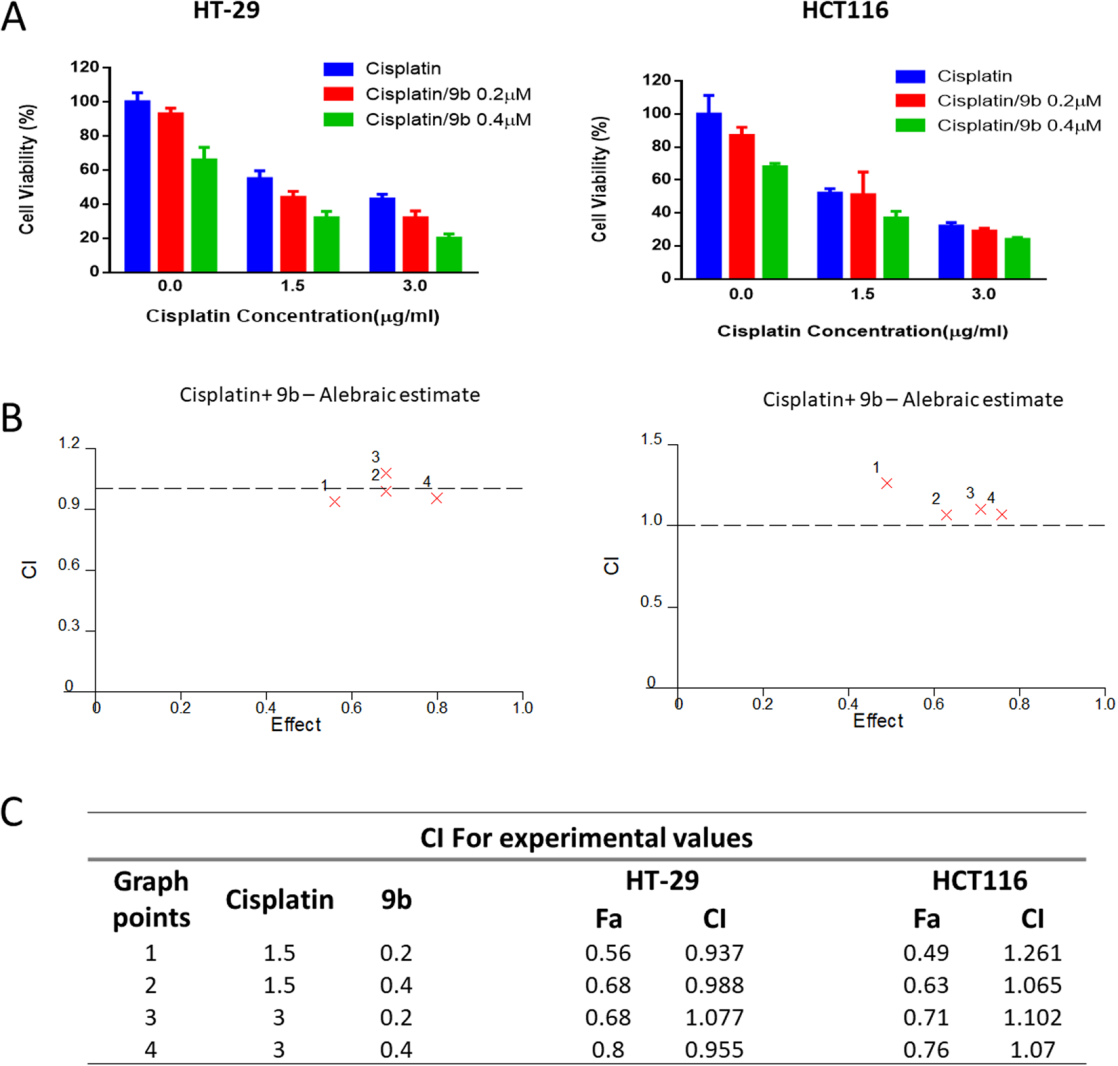
Cisplatin and **9b** combination. (**A**) HT-29 and HCT116 cell lines were treated with cisplatin or/and **9b** at the indicated concentrations for 72 h, and MTT assay was used to determine the cell viability. (**B**) Cisplatin/**9b** combination algebraic estimate calculated by Calcusyn software. (**C**) A table showing the fraction affected (Fa) and cisplatin/**9b** combination indices (CI) at the indicated concentrations in colorectal cell lines. CI <1, CI = 1 and CI >1 indicate synergism, additive effect, and antagonism, respectively.

**Figure 7:**
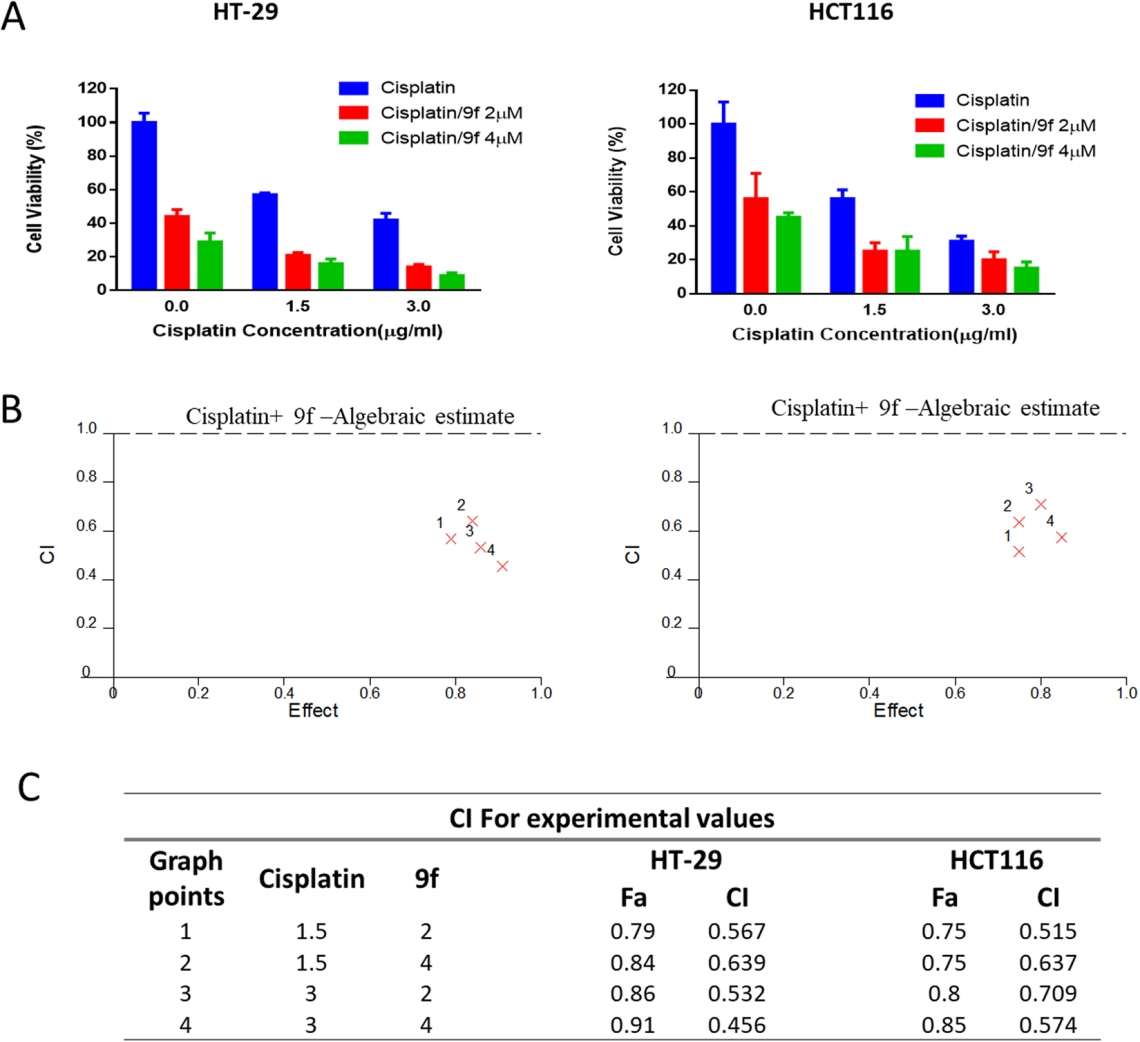
Cisplatin and 9f combination. (**A**) HT-29 and HCT116 cell lines were treated with cisplatin or/and **9f** at the indicated concentrations for 72 h, and MTT assay was used to determine the cell viability. (**B**) Cisplatin/**9f** combination algebraic estimate calculated by Calcusyn software. (**C**) A table showing the fraction affected (Fa) and cisplatin/**9f** combination indices (CI) at the indicated concentrations in colorectal cell lines. CI < 1, CI = 1 and CI > 1 indicate synergism, additive effect, and antagonism, respectively.

Cisplatin, a platinum-based anticancer agent, has been used in different combination regimes in CRC therapy to combat single drug cell resistance^37^. Therefore, we have explored cisplatin and compounds **9b** and **9f** combination as a potential therapeutic protocol. HT-29 and HCT116 cells were treated with different doses of cisplatin (1.5 and 3.0 µg/ml), **9b** (0.2 and 0.4 µM) and **9f** (2.0 and 4.0 µM) alone or in combinations. As a single compound, the IC_50_ of cisplatin was in the range of 1.81–1.93 µg/ml in HT-29 and HCT116 cells, respectively. Whereas the IC_50_ values of **9b** were 0.38 and 0.53 µM in HCT116 and HT-29 cells, respectively. While in **9f**, the IC_50_ values were 2.56 and 3.96 µM in HT-29 and HCT116 cells, respectively. Furthermore, the combination treatment of cisplatin with **9b** was weakly synergistic at 1.5 µg/ml concentration of cisplatin and 0.2 µM of **9b** in HT-29 cells. An additive effect was detected in HT-29 at 0.4 µM of **9b** with 1.5 and 3 µg/ml of cisplatin. While no synergistic effect was observed in HCT116 cells in any of the tested doses (Fig. 6), Interestingly, the combination treatment of cisplatin and **9f** was synergistic in all the tested concentrations (Fig. 7). These promising results need further investigations to illustrate the exact mechanism of synergy.

## Conclusions

In summary, we have identified a new series of low molecular weight compounds that possess potent anticancer activities with potential use as ERMA leads. The synthetic methodologies followed were simple, efficient, and economical. Several of the developed low molecular weight scaffolds possess high potency with IC_50_ values ranging from 0.25 µM to 3.96 µM in CRC cell lines. Several molecular techniques used in this study revealed the anticancer role of the developed compounds through the activation of energy restriction cellular responses and induction of cell apoptosis. Moreover, the tested compounds mediated glucose uptake inhibition, decreased NADPH/NADP+ ratio, and facilitated ROS generation, ultimately promoted cell death. The promising anticancer activity of the synthesized compounds and their ability to synergize cisplatin in targeting CRC cell lines supported the possibility for their combination with conventional chemotherapeutic agents specifically with compound **9f**. Finally, the current work highlights the potential role of the newly developed ERMAs, individually or in combination, in targeting CRC. However, further investigations are required to explore the potential benefits of these novel compounds in different types of cancers and their compatibility with other chemotherapeutic agents.

## Methods

### General chemistry

All used reagents were purchased from commercial suppliers without further purification. The reactions were carried out in oven-dried or flamed graduated vessels. Solvents were dried and purified by conventional methods before use. All reactions were monitored using Merck aluminum plated pre-coated with silica gel PF254 and detected by visualization of the plate under UV lamp (λ = 254 or 365). Column chromatography was performed using silica gel 60, 0.040–0.063 mm (230–400 mesh). ^1^H and ^13^C NMR spectra were recorded on a Bruker Avance DPX-300 MHz and DPX-500 MHz instruments. Splitting patterns are designated as s, singlet; d, doublet; dd, doublet of doublet; t, triplet; q, quartet; m, multiplet; br, broad. Chemical shifts (δ) are given in ppm with reference to TMS as an internal standard. High-resolution mass spectra (HRMS) were measured by Electrospray Ionization (ESI) on a Bruker APEX-IV instrument. The samples were dissolved in acetonitrile and infused using a syringe pump with a flow rate of 120 μL/min. External calibration was conducted using Arginine cluster in a mass range *m*/*z* 175–871. For all HRMS data, mass error: 0.00–0.50 mDa.

### General procedure for the preparing 7-Methoxycarbonylamino-4-substituted Coumarins 3 a–c

A mixture of 10 mmol (1 eq) of *m*-(N-methoxycarbonylamino) phenol **2** was added in portions to 15 ml of concentrated H_2_SO_4_ followed by dropwise addition of 11 mmol (1.1 eq) of appropriate β-Ketoester ester derivatives with starring and cooling (10–15 °C). The mixture was further stirred for 3 h, upon completion 40 ml of ice-water was added and stirred until crystals formed. The precipitate was filtrated, washed with water and methanol and dried to give the desired product, which was used in the next steps without further purification.

### 7-Methoxycarbonylamino-4-Methylcoumarin 3a

The title compound was obtained starting from the reaction of **2** with ethyl acetoacetate as described above (white solid, 72% yield, mp 255–257 °C). ^1^H NMR (300 MHz, DMSO-*d*_6_, in ppm): δ = 2.47 (s, 3H), 3.67 (s, 3H), 6.18 (s, 1H), 7.35 (dd, *J* = 8.7, 1.8 Hz, 1H), 7.49 (d, *J* = 1.8 Hz, 1H), 7.63 (d, *J* = 8.7 Hz, 1H), 10.13 (s, NH). ^13^C NMR (75 MHz, DMSO-*d*_6_, in ppm) δ = 18.5, 52.5, 104.9, 112.4, 114.7, 114.8, 126.5, 143.3, 153.7, 154.3, 154.3, 160.5. HRMS (ESI): calcd. for C_12_H_10_NO_4_ [M-H]^−^: 232.06153; found 232.06277.

### 7-Methoxycarbonylamino-4-Ethylcoumarins (3b)

The title compound was obtained starting from the reaction of **2** with ethyl propioacetate as described above (white solid, 60% yield, mp 192–194 °C). ^1^H NMR (300 MHz, DMSO-*d*_6_, in ppm): δ = 1.18 (t, *J* = 7.4 Hz, 3C), 2.74 (q, *J* = 7.4 Hz, 2C), 3.67 (s, 3H), 6.13 (s, 1H), 7.35 (dd, *J* = 8.7, 2.0 Hz, 1H), 7.51 (d, *J* = 2.0 Hz, 1H), 7.69 (d, *J* = 8.8 Hz, 1), 10.12 (s, NH). ^13^C NMR (75 MHz, DMSO-*d*_6_, in ppm): δ = 12.7, 24.4, 52.5, 105.1, 111.3, 114.0, 114.8, 126.1, 143.1, 154.4, 154.5, 158.5, 160.8. HRMS (ESI): calcd. for C_13_H_12_NO_4_ [M-H]^−^: 246.07718; found 246.07859.

### 7-Methoxycarbonylamino-4-Propylcoumarins (3c)

The title compound was obtained starting from the reaction of **2** with ethyl butyrylacetate as described above (white solid, 89% yield, mp 187–188 °C). ^1^H NMR (300 MHz, DMSO-*d*_6_, in ppm): δ = 0.93 (t, *J* = 7.3 Hz, 3H), 1.59 (m, 2H), 2.69 (t, *J* = 7.5 Hz, 2H), 3.67 (s, 3H), 6.14 (s, 1H), 7.35 (d, *J* = 8.8 Hz, 1H), 7.52 (br s, 1H), 7.71 (d, *J* = 8.8 Hz, 1H), 10.13 (s, NH). ^13^C NMR (75 MHz, DMSO-*d*_6_, in ppm): δ = 14.2, 21.8, 33.2, 52.6, 105.1, 111.5, 114.1, 114.8, 126.3, 143.2, 154.3, 154.6, 157.0, 160.7. HRMS (ESI): calcd. for C_14_H_14_NO_4_ [M-H]^−^: 260.09283; found 260.09396.

### General procedure for preparing 7-amino-4-substituted Coumarins 4a–c

A suspension of 15 mmol of **(3a–c)** in 15 ml of 45% of KOH was stirred at 80–90 °C for 3 h until completion. The mixture was cooled and diluted with 50 ml water; the solution was acidified with concentrated HCl to 7–8 pH with stirring and cooling until crystallization occurred. The precipitate was filtrated, washed with water and ether then air-dried.

### 7-Amino-4-methylcoumarin 4a

The title compound was obtained starting from the reaction of **3a** (brown solid, 85% yield, mp 222–223 °C). ^1^H NMR (300 MHz, DMSO-*d*_6_, in ppm): ^1^H NMR (300 MHz, DMSO-*d*_6_, in ppm): δ = 2.26 (s, 3H), 5.86 (s, 1H), 6.06 (br s, NH_***2***_), 6.36 (d, *J* = 2.0 Hz, 1H), 6.52 (dd, *J* = 8.6, 2.0 Hz, 1H), 7.36 (d, *J* = 8.6 Hz, 1H). ^13^C NMR (75 MHz, DMSO-d_6_, in ppm) δ = 18.5, 99.0, 108.0, 109.3, 111.7, 126.7, 153.6, 154.3, 156.0, 161.2. HRMS (ESI): calcd. for C_10_H_9_NO_2_Na [M + Na]^+^: 198.05255; found 198.05240.

### 7-Amino-4-ethylcoumarin (4b)

The title compound was obtained starting from the reaction of 3b (brown solid, 50% yield, mp 223–224 °C). ^1^H NMR (300 MHz, DMSO-*d*_6_, in ppm): δ = 1.15 (t, *J* = 7.5 Hz, 3C), 2.59 (q, *J* = 7.5 Hz, 2H), 5.82 (s, 1H), 6.05 (br s, N***H***_***2***_), 6.37 (d, *J* = 2.1 Hz, 1H), 6.52 (dd, *J* = 8.7, 2.1 Hz, 1H), 7.41 (d, *J* = 8.7 Hz, 1H). ^13^C NMR (75 MHz, DMSO-*d*_6_, in ppm) δ = 13.2, 24.5, 99.2, 106.1, 108.4, 111.8, 126.3, 153.4, 156.1, 159.3, 161.5. HRMS (ESI): calcd. for C_12_H_12_NO_2_ [M + H]^+^: 190.08626; found 190.08635

### 7-Amino-4-propylcoumarin (4c)

The title compound was obtained starting from the reaction of **3c** (brown solid, 62.5% yield, mp 203–204 °C). ^1^H NMR (300 MHz, DMSO-*d*_6_, in ppm): δ = 0.91 (t, *J* = 7.3 Hz, 3H), 1.56 (m, 2H), 2.59 (t, *J* = 7.6 Hz, 2H), 5.82 (s, 1H), 6.05 (br s, N***H***_***2***_), 6.37 (d, *J* = 2.1 Hz, 1H), 6.52 (dd, *J* = 8.7, 2.1 Hz, 1H), 7.40 (d, *J* = 8.7 Hz, 1H). ^13^C NMR (75 MHz, DMSO-d_6_, in ppm) δ = 14.2, 22.1, 33.3, 99.2, 107.0, 108.6, 111.7, 126.4, 153.4, 156.2, 157.7, 161.3. HRMS (ESI): calcd. for C_12_H_13_NO_2_Na [M + Na]^+^: 226.08385; found 226.08362.

### General procedure for the synthesis of N-(4-Substituted -2-Oxo-2H-Chromen-7-ylcarbamothioyl)benzamide derivative 6**a–**c

Phenyl isothiocyanate (1.1 mmol, 1.1 eq) was added dropwise to a stirred solution of **4a–c** (1 mmol, 1eq) in 10 ml acetone at room temperature. The mixture was refluxed for about 3–4 h. The mixture was cooled, diluted with ice-water then stirred for an additional 30 min. The precipitate was filtered washed with water, and air-dried to produce the corresponding products **6a–c**.

### N-(4-Methyl-2-oxo-2H-chromen-7-ylcarbamothioyl)benzamide 6a

The title compound was obtained starting from **4a** (bright yellow solid, 95% yield, mp 201–203 °C). ^1^H NMR (300 MHz, DMSO-*d*_6_, in ppm): δ = 2.41 (s, 3H), 6.35 (s, 1H), 7.51 (pseudo t, 2H), 7.60 (dd, *J* = 8.6, 2.0 Hz, 1H), 7.62 (t, *J* = 7.4 Hz, 1H), 7.76 (d, *J* = 8.6 Hz, 1H), 7.95 (d, *J* = 7.2 Hz, 2H), 8.1 (s, 1H), 11.66 (s, NH), 12.79 (s, Ar-NH). ^13^C NMR (125 MHz, DMSO-d_6_, in ppm) δ = 18.5, 111.1, 114.2, 117.9, 120.3, 126.1, 129.0, 129.2, 132.5, 133.7, 141.5, 153.3, 153.5, 160.2, 168.7, 179.5. HRMS (ESI): calcd. for C_18_H_13_N_2_O_3_S [M-H]^−^: 337.06524; found 337.06745.

### N-(4-Ethyl-2-oxo-2H-cromen-7-ylcarbamothioyl)benzamide (6b)

The title compound was obtained starting from **4b (**bright yellow solid, 54% yield, mp 197–200 °C). ^1^H NMR (300 MHz, DMSO-*d*_6_, in ppm): δ = 1.21 (t, *J* = 7.4 Hz, 3H), 2.81 (q, *J* = 7.4 Hz, 2H), 6.28 (s, 1H), 7.51 (pseudo t, 2H), 7.60 (dd, *J* = 6.7, 2.2 Hz, 1H), 7.64 (t, *J* = 7.7 Hz, 1H), 7.81 (d, *J* = 8.7 Hz, 1H), 7.95 (d, *J* = 7.4 Hz, 2H), 8.08 (d, *J* = 2.2 Hz, 1H), 11.68 (s, NH), 12.78(s, Ar-NH). ^13^C NMR (75 MHz, DMSO-*d*_6_, in ppm) δ = 12.7, 24.5, 111.3, 112.3, 117.1, 120.4, 125.7, 129.0, 129.3, 132.5), 133.8, 141.5, 153.6, 158.2, 160.5, 168.7, 179.6. HRMS (ESI): calcd. for C_19_H_15_N_2_O_3_S [M-H]^−^: 351.08089; found 351.08330.

### N-(4-Propyl-2-oxo-2H-chromen-7-ylcarbamothioyl)benzamide (6c)

The title compound was obtained starting from **4a** (bright yellow solid, 97% yield, mp 184–186 °C). ^1^H NMR (300 MHz, DMSO-*d*_6_, ppm): δ = 0.96 (t, *J* = 7.3 Hz, 3H), 1.47 (m, 2H), 2.76 (t, *J* = 7.7 Hz, 2H), 6.29 (s, 1H), 7.50 (pseudo t, 2H), 7.60 (m, 1H), 7.63 (m, 1H), 7.84 (d, *J* = 8.7 Hz, 1H), 7.94 (dd, *J* = 5.2, 1.5 Hz, 2H), 8.07 (d, *J* = 1.8 Hz, 1H), 11.67(s, NH), 12.77(s, Ar-NH). ^13^C NMR (75 MHz, DMSO-*d*_6_, in ppm): δ = 14.2, 21.7, 33.2, 111.3, 113.3, 117.1, 120.4, 125.9, 128.9, 132.6, 132.7, 133.8, 141.5, 153.6, 156.7, 160.4, 168.7, 179.5. HRMS (ESI): calcd. for C_20_H_17_N_2_O_3_S [M-H]^−^: 365.09654; found 365.09877.

### General procedure for the synthesis of 7-Aminocoumarin thiourea derivatives (7**a–**c)

To a stirred solution of **6a–c** (1 mmol) in 20 ml methanol at room temperature was added 5 ml of aqueous (1 N) NaOH. The mixture was refluxed for 3–4 h at 80 °C until hydrolysis was completed. The mixture was cooled and diluted with 20 ml ice-water, then the mixture was acidified to pH 7 using aqueous 1N HCl. The precipitate formed was filtered and dried. The products were further purified by column chromotography using gradient system of (*n*-hexane-ethyl acetate).

### 1-(4-Methyl-2-oxo-2H-chromen-7-yl)thiourea (7a)

The title compound was obtained starting from **6a** (brown solid, 97% yield, mp 219–222 °C). ^1^H NMR (300 MHz, DMSO-*d*_6_, ppm): δ = 2.30 (s, 3H), 6.24 (s, 1H), 7.36 (dd, *J* = 8.7, 2.1 Hz, 1H), 7.66 (d, *J* = 8.7 Hz, 1H), 7.78 (d, *J* = 2.1 Hz, 1H), 10.08 (s, N***H***). ^13^C NMR (75 MHz, DMSO-*d*_6_, in ppm): δ = 18.5, 108.5, 112.9, 115.7, 118.2, 126.1, 143.5, 153.7, 153.8, 160.5, 181.6. HRMS (ESI): calcd. for C_11_H_9_N_2_O_2_S [M-H]^−^ :233.03792; found 233.03536.

### 1-(4-Ethyl-2-oxo-2H-chromen-7-yl)thiourea (7b)

The title compound was obtained starting from **6b** (brown solid, 71% Yield, mp 237–239 °C). ^1^H NMR (300 MHz, DMSO-*d*_6_, ppm): δ = 1.19 (t, *J* = 7.4 Hz, 3H), 2.77 (q, *J* = 7.4 Hz, 2H), 6.18 (s, 1H), 7.36 (d, *J* = 8.7 Hz, 1H), 7.71 (d, *J* = 8.7 Hz, 1H), 7.78 (br s, 1H), 10.06 (br s, N***H***). ^13^C NMR (75 MHz, DMSO-*d*_6_, in ppm): δ = 12.8, 24.5, 108.7, 111.0, 114.9, 118.2, 125.6, 143.3, 153.9, 158.4, 160.8, 181.6. HRMS (ESI): calcd. for C_12_H_11_N_2_O_2_S [M-H]^−^: 247.05467; found 247.05577.

### 1-(2-Oxo-4-propyl-2H-chromen-7-yl)thiourea (7c)

The title compound was obtained starting from **6c** (brown solid, 97% yield, mp 234–235 °C). ^1^H NMR (300 MHz, DMSO-*d*_6_, in ppm): δ = 0.94 (t, *J* = 7.4 Hz, 3H), 1.60 (m, 2H), 2.71 (t, *J* = 7.4 Hz, 2H), 6.19 (s, 1H), 7.34 (dd, *J* = 8.7, 2.0 Hz, 1H), 7.71 (d, *J* = 8.7 Hz, 1H), 7.78 (d, *J* = 2.0 Hz, 1H), 10.06 (br s, NH). ^13^C NMR (75 MHz, DMSO-*d*_6_, in ppm): δ = 14.2, 21.8, 33.2, 108.8, 112.0, 115.0, 118.2, 125.8, 143.3, 154.1, 156.9, 160.6, 181.6. HRMS (ESI): calcd. for C_13_H_13_N_2_O_2_S [M-H]^−^: 261.06922; found 261.06972.

### General procedure for the synthesis of target compounds 9**a–**i, 10**a–**c, 10e-i, 11**a–**c, 11e–h

The appropriate α-bromophenone **8a–i** (1.1 mmol) was added with stirring to a solution of a particular thiourea derivative **7a–c** (1 mmol) in 10 ml dry ethanol. The mixture was refluxed for 4 h. Upon completion, the mixture was cooled and poured into 20 ml of ice-water and stirred for additional 30 min. The pH of the solution was adjusted to 8 using 1N Na_2_CO_3_. The product was filtered off, washed with water and ether then purified by column chromatography (ethyl acetate- chloroform: 10–90%).

### 4-Methyl-7-(4-phenylthiazol-2-ylamino)-2H-chromen-2-one (9a)

The title compound was obtained starting from the reaction of **7a** and **8a** (bright Brown solid, 97% yield, mp 255–257 °C). ^1^H NMR (300 MHz, DMSO-*d*_6_, in ppm): δ = 2.53 (s, 3H), 6.16 (s, 1H), 7.31 (t, *J* = 7.3 Hz, 1H), 7.45 (s, 1H), 7.47 (m, 2H), 7,48 (d, *J* = 8.7 Hz, 1H), 7.70 (d, *J* = 8.7 Hz, 1H), 7.91 (d, *J* = 7.3 Hz, 2H), 7.99 (d, *J* = 2.0 Hz, 1H), 10.82 (s, NH). ^13^C NMR (75 MHz, DMSO-*d*_6_, in ppm): δ = 18.5, 103.3, 105.0, 111.5, 113.7, 113.9, 126.1, 126.7, 129.3, 129.6, 134.8, 144.7, 150.8, 153.9, 154.9, 162.8, 160.8. HRMS (ESI): calcd. for C_19_H_13_N_2_O_2_S [M-H]^−^: 333.07032; found 333.06892.

### 7-(4-(4-Bromophenyl)thiazol-2-ylamino)-4-methyl-2H-chromen-2-one (9b)

The title compound was obtained starting from the reaction of **7a** and **8b** (yellow solid, 97% yield, mp 292–293 °C). ^1^H NMR (300 MHz, DMSO-*d*_6_, in ppm): δ = 2.37 (s, 3H), 6.16 (s, 1H), 7.46 (dd, *J* = 8.7, 1.9 Hz, 1H), 7.53 (s, 1H), 7.64 (d, *J* = 8.5 Hz, 2H), 7.70 (d, *J* = 8.7 Hz, 1H), 7.86 (d, *J* = 8.5 Hz, 2H), 7.93 (d, *J* = 1.9 Hz, 1H), 10.84 (s, NH). ^13^C NMR (75 MHz, DMSO-*d*_6_, in ppm): δ = 18.5, 103.4, 106.0, 111.6, 113.8, 114.0, 121.4, 126.7, 128.1, 132.2, 134.0, 144.6, 149.6, 153.8, 154.9, 160.8, 162.9. HRMS (ESI): calcd. for C_19_H_12_BrN_2_O_2_S [M-H]^−^: 412.97875; found 412.98069.

### 7-(4-(4-Chlorophenyl)thiazol-2-ylamino)-4-methyl-2H-chromen-2-one (9c)

The title compound was obtained starting from the reaction of **7a** and **8c** (yellow solid 60% yield, mp 286–289 °C). ^1^H NMR (300 MHz, DMSO-*d*_6_, in ppm): δ = 2.37 (s, C***H***_***3***_), 6.17 (s, H-3), 7.47 (dd, *J* = 8.7, 2.1 Hz, H-6), 7.51 (d, *J* = 8.5 Hz, H-3′′, H-5′′), 7.52 (s, H-5’), 7.67 (d, *J* = 8.7 Hz, H-5), 7.92 (d, *J* = 8.5 Hz, H-2′′,6′′), 7.94 (s, H-8), 10.84 (s, N***H***). ^13^C NMR (75 MHz, DMSO-*d*_6_, in ppm): δ = 18.5, 103.3, 105.9, 111.6, 113.8, 114.0, 126.7, 127.8, 129.3, 132.8, 133.6, 144.6, 149.6, 153.8, 154.9, 160.8, 162.8. HRMS (ESI): calculated for C_19_H_12_ClN_2_O_2_S [M-H]^−^: 367.03025; found 367.02719.

### 7-(4-(4-Ethoxyphenyl)thiazol-2-ylamino)-4-methyl-2H-chromen-2-one (9d)

The title compound was obtained starting from the reaction of **7a** and **8d** (brown solid, 86% yield, mp 222–224 °C). ^1^H NMR (300 MHz, DMSO-*d*_6_, in ppm): δ = 1.31 (t, *J* = 6.8 Hz, 3H), 2.37 (s, 3H), 4.12 (q, *J* = 6.8 Hz, 2H), 6.16 (s, 1H), 6.99 (d, *J* = 8.4 Hz, 2H), 7.26 (s, H-5’), 7.43 (d, *J* = 8.6 Hz, 1H), 7.69 (d, *J* = 8.6 Hz, 1H),), 7.81 (d, *J* = 8.4 Hz, 2H), 8.00 (br s, H-8), 10.78 (s, NH). ^13^C NMR (75 MHz, DMSO-*d*_6_, in ppm): δ = 14.9, 18.5, 63.6, 102.7, 103.3, 111.4, 113.7, 113.9, 115.1, 126.6, 127.5, 132.5, 144.8, 150.7, 153.9, 154.9, 158.8, 160.9, 162.5. HRMS (ESI): calcd. for C_21_H_19_N_2_O_3_S [M + H]^+^: 379.11109; found 379.11102.

### 7-(4-(3-Methoxyphenyl)thiazol-2-ylamino)-4-methyl-2H-chromen-2-one (9e)

The title compound was obtained starting from the reaction of **7a** and **8e** (brown solid, 95% yield, mp 210–212 °C). ^1^H NMR (300 MHz, DMSO-*d*_6_, in ppm): δ = 2.46 (s, 3H), 3.86 (s, 3H), 6.16 (s, 1H), 6.87 (dd, *J* = 8.1, 2.4 Hz, 1H), 7.35 (pseudo t, 1H), 7.45 (d, *J* = 8.7 Hz, 1H), 7.47 (s, 1H),7.48 (s, 1H), 7.49 (m, 1H), 7.70 (d, *J* = 8.7 Hz, 1H), 7.99 (d, *J* = 1.9 Hz, 1H), 10.82 (s, NH). ^13^C NMR (75 MHz, DMSO-*d*_6_, in ppm): δ = 18.5, 55.6, 103.4, 105.4, 111.5, 112.0, 113.6, 113.7, 113.9, 118.6, 126.7, 130.4, 136.1, 144.7, 150.7, 153.8, 154.9, 160.1, 160.8, 162.5. HRMS (ESI): calcd. for C_20_H_15_N_2_O_3_S [M-H]^−^: 363.08089; found 363.08302.

### 4-Methyl-7-(4-(3-Nitrophenyl)thiazol-2-ylamino)-2H-chromen-2-one (**9f**)

The title compound was obtained starting from the reaction of **7a** and **8f** (bright brown solid, 90% yield, mp 298–299 °C). ^1^H NMR (300 MHz, DMSO-*d*_6_, in ppm): δ = 2.37 (s, 3H), 6.18 (s, 1H), 7.46 (dd, *J* = 8.7, 1.9 Hz, 1H), 7.71 (dd, *J* = 7.9, 8.0 Hz, 1H), 7.75 (d, *J = *8.7 Hz, 1H), 7.78 (s, 1H), 7.89 (d, *J* = 1.9 Hz, 1H), 8.14 (dd, *J* = 8.0, 1.9 Hz, 1H), 8.38 (d, *J* = 7.9 Hz, 1H), 8.67 (s, 1H), 10.89 (s, N*H*). ^13^C NMR (75 MHz, DMSO-*d*_6_, in ppm): δ = 18.5, 103.4, 107.8, 112.0, 113.2, 113.9, 120.5, 122.8, 126.7, 130.9, 132.3, 136.3, 144.5, 148.4, 153.7, 154.7, 154.9, 160.7, 160.7. HRMS (ESI): calcd. for C_19_H_12_N_3_O_4_S [M-H]^−^: 378.05540; found 378.05367.

### 7-(4-(3-Fluorophenyl)thiazol-2-ylamino)-4-methyl-2H-chromen-2-one (9g)

The title compound was obtained starting from the reaction of **7a** and **8g** (bright brown solid, 90% yield, mp 280–281 °C). ^1^H NMR (300 MHz, DMSO-*d*_6_, in ppm): δ = 2.48 (s, 3H), 2.73, 6.17 (s, 1H), 7.16 (dd, *J* = 8.6, 2.2 Hz, 1), 7.49 (m, 1H), 7.50 (m, 1H), 7.59 (s, 1H), 7.59 (d, ^2^*J*^H-F^ = 2.0 Hz, H-2”), 7.69 (d, *J* = 8.2 Hz, 1H), 7.72 (d, *J* = 7.9 Hz, 1H), 7.93 (d, *J* = 2.1 Hz, 1H), 10.84 (s, NH). ^13^C NMR (75 MHz, DMSO-*d*_6_, in ppm): δ = 18.5, 103.4, 106.5, 111.6, 112.7 (d, ^2^*J*^C-F^ = 22.9 Hz, 113.8, 114.0, 115.0 (d, ^2^*J*^C-F^ = 21.0 Hz), 122.2, 126.7, 131.2 (d, ^3^*J*^C-F^ = 8.6 Hz), 137.1 (d, ^3^*J*^C-F^ = 8.38 Hz), 144.6, 149.5, 153.8, 154.8, 160.8, 162.7, 163.1 (d, ^1^*J*^C-F = ^242.6 Hz). HRMS (ESI): calcd. for C_19_H_14_FN_2_O_2_S [M + H]^+^: 353.07545; found 353.07583.

### 4-Methyl-7-(4-(2-nitrophenyl)thiazol-2-ylamino)-2H-chromen-2-one (**9h**)

The title compound was obtained starting from the reaction of **7a** and **8h** (bright brown solid, 90% yield, mp dcomp. at 338 °C). ^1^H NMR (300 MHz, DMSO-*d*_6_, in ppm): δ = 2.36 (s, 3H), 6.16 (s, 1H), 7.31 (dd, *J* = 8.8, 1.9 Hz, 1H), 7.46 (s, 1H), 7.57 (pseudo t, 1), 7.63 (d, *J* = 8.8 Hz, 1H), 7.70 (m, 1H), 7.77 (br s, 1H), 7.82 (d, *J = *7.9 Hz, 2H), 10.79 (s, NH). ^13^C NMR (75 MHz, DMSO-*d*_6_, in ppm): δ = 18.5, 103.5, 109.1, 111.6, 113.8, 113.9, 124.1, 126.7, 128, 129.7, 130.5, 132.6), 144.3, 146.4, 149.4, 153.7, 154.9, 160.7, 162.7. HRMS (ESI): calcd. for C_19_H_12_N_3_O_4_S [M-H]^−^: 378.05540; found 378.05821.

### 7-(4-(2-Methoxyphenyl)thiazol-2-ylamino)-4-methyl-2H-chromen-2-one (9i)

The title compound was obtained starting from the reaction of **7a** and **8i** (bright brown solid, 88% yield, mp 224–226 °C). ^1^H NMR (300 MHz, DMSO-*d*_6_, in ppm): δ = 2.37 (s, 3H), 3.90 (s, 3H), 6.16 (s, 1H), 7.09 (m, 1H), 7.11 (d, *J* = 7.7 Hz, 1H), 7.31 (pseudo t, 1H), 7.48 (dd, *J* = 8.7, 2.0 Hz, 1H), 7.50 (s, 1H), 7.71 (d, *J* = 8.7 Hz, 1H), 7.95 (d, *J* = 2.0 Hz, 1H), 8.11 (dd, *J* = 7.6, 1.6 Hz, 1H), 10.75 (s, NH). ^13^C NMR (75 MHz, DMSO-*d*_6_, in ppm): δ = 18.5, 56.0, 103.2, 109.0, 111.4, 112.2, 113.6, 113.9, 121.1, 123.0, 126.7, 129.3, 129.5, 144.8, 146.7, 153.8, 154.9, 157.2, 160.8, 160.9. HRMS (ESI): calcd. for C_20_H_16_N_2_O_3_S [M-H]^−^: 363.08089; found 363.08277.

### 4-Ethyl-7-(4-phenylthiazol-2-ylamino)-2H-chromen-2-one (10a)

The title compound was obtained starting from the reaction of **7b** and **8a (**bright brown solid, 80% yield, mp 215–216 °C). ^1^H NMR (300 MHz, DMSO-*d*_6_, in ppm): δ = 1.21 (t, *J* = 7.4 Hz, 3HC), 2.78 (q, *J* = 7.4 Hz, 2H), 6.12 (s, 1H), 7.31(d, *J* = 7.0 Hz, 1H), 7.31 (t, *J* = 7.31, 1H), 7.43 (m, 2H), 7.44 (s, 1H), 7.46 (d, *J* = 8.8 Hz, 1H), 7.76 (d, *J* = 8.8 Hz, 1H), 7.93 (d, *J* = 7.9 Hz, 2H), 7.99 (br s, 1H), 10.82 (s, NH). ^13^C NMR (75 MHz, DMSO-*d*_6_, in ppm): δ = 12.9, 24.5, 103.5, 105.0, 109.6, 112.9, 114.0, 126.2, 126.3, 128.3, 129.3, 134.8, 144.6, 150.8, 155.0, 158.7, 161.1, 162.6. HRMS (ESI): calcd. for C_20_H_15_N_2_O_2_S [M-H]^−^: 347.08597; found 347.08669.

### 7-(4-(4-Bromophenyl)thiazol-2-ylamino)-4-ethyl-2H-chromen-2-one (10b)

The title compound was obtained starting from the reaction of **7b** and **8b (**yellow solid, 98% yield, mp 263–265 °C). ^1^H NMR (300 MHz, DMSO-*d*_6_, in ppm): δ = 1.21 (t, *J* = 7.4 Hz, 3H), 2.79 (q, *J* = 7.4 Hz, 2H), 6.12 (s, 1H), 7.46 (dd, *J* = 8.8, 2.1 Hz, 1H), 7.54 (s, 1H), 7.64 (d, *J* = 8.5 Hz, 2H), 7.75 (d, *J* = 8.8 Hz, 1H), 7.86 (d, *J* = 8.5 Hz, 2H), 7.93 (d, *J* = 2.1 Hz, 1H), 10.84 (s, NH). ^13^C NMR (75 MHz, DMSO-*d*_6_, in ppm): δ = 12.9, 24.5, 103.5, 106.0, 109.7, 113.0, 114.0, 121.4, 126.3, 128.1, 132.2, 134.0, 144.5, 149.6, 155.0, 158.6, 161.1, 162.8. HRMS (ESI) m/z, calculated for C_20_H_14_BrN_2_O_2_S [M-H]^−^ = 426.99440, found 426.99681.

### 7-(4-(4-Chlorophenyl)thiazol-2-ylamino)-4-ethyl-2H-chromen-2-one (10c)

The title compound was obtained starting from the reaction of **7b** and **8c (**yellow solid, 97% yield, mp 258–260 °C. ^1^H NMR (300 MHz, DMSO-*d*_6_, in ppm): δ = 1.24 (t, *J* = 7.2 Hz, 3H), 2.80 (q, *J* = 7.2 Hz, 2H), 6.14 (s, 1H), 7.50 (d, *J* = 8.5 Hz, 1H), 7.54 (d, *J* = 8.2 Hz, 2H), 7.55 (s, 1H), 7.77 (d, *J* = 8.5 Hz, 1H), 7.96 (d, *J* = 8.2 Hz, 2H), 7.97 (s, 1H), 10.85 (s, N*H*). ^13^C NMR (75 MHz, DMSO-*d*_6_, in ppm): δ = 12.8, 24.4, 103.5, 105.8, 109.6, 112.9, 114.0, 126.2, 127.8, 129.3, 132.7, 133.6, 144.5, 149.6, 154.8, 158.6, 161.0, 162.8. HRMS (ESI): calcd. for C_20_H_14_ClN_2_O_2_S [M-H]^−^: 381.04700; found 381.04484.

### 4-Ethyl-7-(4-(3-methoxyphenyl)thiazol-2-ylamino)-2H-chromen-2-one (10e)

The title compound was obtained starting from the reaction of **7b** and **8e (**brown solid, 80% yield, mp 215–217 °C). ^1^H NMR (300 MHz, DMSO-*d*_6_, in ppm): δ = 1.20 (t, *J* = 7.4 Hz, 3H), 2.76 (q, *J* = 7.4 Hz, 2H), 3.80 (s, 3H), 6.10 (s, 1H), 6.90 (d, *J* = 8.0 Hz, 1H), 7.35 (pseudo t, 1H),7.45 (d, *J* = 8.8 Hz, 1H), 7.46 (s, 1H), 7.48 (s, 1H), 7.49 (d, *J* = 7.7 Hz, 1H), 7.74 (d, *J* = 8.8 Hz, 1H), 8.00 (br s, 1H), 10.81 (s, NH). ^13^C NMR (75 MHz, DMSO-*d*_6_, in ppm): δ = 12.8, 24.4, 55.6, 103.5, 105.3, 109.6, 112.0, 112.9, 113.6, 113.5, 118.6, 126.2, 130.3, 136.1, 144.6, 150.6, 155.0, 158.6, 160.1, 161.0, 162.4. HRMS (ESI): calcd. for C_21_H_17_N_2_O_3_S [M-H]^−^: 377.09654; found 377.09832.

### 4-Ethyl-7-(4-(3-nitrophenyl)thiazol-2-ylamino)-2H-chromen-2-one (10f)

The title compound was obtained starting from the reaction of **7b** and **8f (**bright brown solid, 86% yield, mp 303–304 °C). ^1^H NMR (300 MHz, DMSO-*d*_6_, in ppm): δ = 1.20 (t, *J* = 7.3 Hz, 3H), 2.76 (q, *J* = 7.3 Hz, 2H), 6.10 (s, 1H), 7.45 (d, *J* = 7.4 Hz, 1H), 7.72 (m, 1H), 7.74 (d, *J* = 7.4 Hz, 1H), 7.77 (s, 1H), 7.89 (s, 1H), 8.14 (d, *J* = 8.1 Hz, 1H), 8.33 (d, *J* = 7.8 Hz, 1H), 8.66 (s, 1H), 10.87 (s, NH). ^13^C NMR (75 MHz, DMSO-*d*_6_, in ppm): δ = 12.8, 24.5, 103.6, 107.7, 109.7, 113.1, 114.0, 120.5, 122.8, 126.2, 130.9, 132.3, 136.3, 144.3, 148.4, 148.9, 154.5, 158.5, 161.0, 163.1. HRMS (ESI): calcd. for C_20_H_14_N_3_O_4_S [M-H]^−^: 392.06995; found 392.06959.

### 4-Ethyl-7-(4-(3-fluorophenyl)thiazol-2-ylamino)-2H-chromen-2-one (10g)

The title compound was obtained starting from the reaction of **7b** and **8g** bright brown solid, 81% yield, mp 245–247 °C). ^1^H NMR (300 MHz, DMSO-*d*_6_, in ppm): δ = 1.18 (t, *J* = 7.2 Hz, 3H), 2.73 (q, *J* = 7.2 Hz, 2H), 6.07 (s, 1H), 7.12 (pseudo t, 1H), 7.45 (d, *J* = 9.2 Hz, 1H), 7.47 (m, 1H), 7.54 (s, 1H), 7.67 (d, ^2^*J*^H-F^ = 2.0 Hz, 1H), 7.70 (d, *J* = 9.2 Hz, 1H), 7.74 (d, *J* = 7.5 Hz, 1H), 7.90 (broad s, 1H), 10.80 (s, NH). ^13^C NMR (75 MHz, DMSO-*d*_6_, in ppm): δ = 12.7, 24.4, 103.5, 106.4, 109.6, 112.7 (d, ^2^*J*^C-F^ = 22.9 Hz), 112.9, 113.9, 115.0 (d, ^2^*J*^C-F^ = 21.0 Hz), 122.1, 126.1, 131.2 (d, ^3^*J*^C-F^ = 8.4 Hz), 137.1 (d, ^3^*J*^C-F^ = 11.9 Hz), 144.4, 149.4, 154.9, 158.5, 161.0, 162.7, 163.1 (d, ^1^*J*^C-F^ = 242.5 Hz). HRMS (ESI): m/z calcd. for C_20_H_16_FN_2_O_2_S [M + H]^+^: 367.09110; found 367.09112.

### 4-Ethyl-7-(4-(2-nitrophenyl)thiazol-2-ylamino)-2H-chromen-2-one (**10h**)

The title compound was obtained starting from the reaction of **7b** and **8h (**bright brown solid, 81% yield, mp 261–263 °C). ^1^H NMR (300 MHz, DMSO-*d*_6_, in ppm): δ = 1.20 (t, *J* = 7.4 Hz, 3H), 2.77 (q, *J* = 7.4 Hz, 2H), 6.11 (s, 1H), 7.29 (dd, *J* = 8.7, 2.1 Hz, 1H), 7.46 (s, 1H), 7.57 (ddd, *J* = 6.5, 8.1, 1.3 Hz, 1H), 7.66 (d, *J* = 8.7 Hz, 1H), 7.68 (m, 1H),7.72 (d, *J* = 2.1 Hz, 1H), 7.83 (d, *J* = 8.1 Hz, 2H), 10.81 (s, N*H*). ^13^C NMR (75 MHz, DMSO-*d*_6_, in ppm): δ = 12.8, 28.9, 103.7, 109.1, 109.8, 113.1, 113.9, 124.1, 126.0, 128.0, 129.7, 130.5, 132.6, 144.2, 146.4, 149.4, 155.1, 158.5, 161.1, 162.7. HRMS (ESI): calcd. for C_20_H_14_N_3_O_4_S [M-H]^−^: 392.07276; found 392.07276.

### 4-Ethyl-7-(4-(2-methoxyphenyl)thiazol-2-ylamino)-2H-chromen-2-one (10i)

The title compound was obtained starting from the reaction of **7b** and **8i** (bright brown solid, 84% yield, mp 200–202 °C). ^1^H NMR (300 MHz, DMSO-*d*_6_, in ppm): δ = 1.21 (t, *J* = 7.4 Hz, 3H), 2.78 (q, *J* = 7.4 Hz, 2H), 3.90 (s, 3H), 6.12 (s, 1H), 7.08 (m, 1H), 7.11 (d, *J* = 7.7 Hz, 1H), 7.30 (pseudo t, 1H), 7.48 (d, *J* = 8.8 Hz, 1H), 7.50 (s, 1H), 7.75 (d, *J* = 8.8 Hz, 1H), 7.96 (d, *J* = 2.0 Hz, 1H), 8.11 (d, *J* = 6.4 Hz, 1H), 10.76 (s, NH). ^13^C NMR (75 MHz, DMSO-*d*_6_, in ppm): δ = 12.9, 24.5, 56.0, 103.4, 108.9, 109.6, 112.2, 112.9, 113.9, 121.1, 123.0, 126.3, 129.3, 129.5, 144.7, 146.8, 155.1, 157.2, 158.7, 160.9, 161.1. HRMS (ESI): calcd. for C_21_H_17_N_2_O_3_S [M-H]^−^: 377.09654; found 377.09654.

### 7-(4-Phenylthiazol-2-ylamino)-4-propyl-2H-chromen-2-one (11a)

The title compound was obtained starting from the reaction of **7c** and **8a (**bright brown solid, 71% yield, mp 230–232 °C). ^1^H NMR (500 MHz, DMSO-*d*_6_, in ppm): δ = 0.94 (t, *J* = 7.0 Hz, 3H), 1.62 (m, 2H), 2.71 (t, *J* = 7.0 Hz, 2H), 6.11 (s, 1H), 7.31 (t, *J* = 6.9 Hz, 1H), 7.45 (s, 1H), 7.46 (m, 2H), 7.48 (d, *J* = 8.6, Hz, 1H), 7.75 (d, *J* = 8.6 Hz,1H), 7.91 (d, *J* = 7.3 Hz, 2H), 8.0 (s, 1H),10.2 (s, NH). ^13^C NMR (125 MHz, DMSO-*d*_6_, in ppm) δ = 14.2, 21.9, 33.3, 103.7, 105.0, 110.7, 113.0, 114.0, 126.1, 126.3, 128.3, 129.2, 134.8, 144.6, 150.8, 155.1, 157.2, 160.9, 162.6. HRMS (ESI): calcd. for C_21_H_17_N_2_O_2_S [M-H]^−^: 361.10162; found 361.09916.

### 7-(4-(4-Bromophenyl)thiazol-2-ylamino)-4-propyl-2H-chromen-2-one (11b)

The title compound was obtained starting from the reaction of **7c** and **8a (**yellow solid, 95% yield, mp 290–293 °C). ^1^H NMR (300 MHz, DMSO-*d*_6_, in ppm): δ = 0.95 (t, *J* = 7.3 Hz, 3H), 1.63 (m, 2H), 2.71 (t, *J* = 7.4 Hz, 2H), 6.11 (s, 1H), 7.48 (dd, *J* = 8.8, 1.9 Hz, 1H), 7.53 (s, 1H), 7.63 (d, *J* = 8.4 Hz, 2H), 7.75 (d, *J* = 8.8 Hz, 1H), 7.85 (d, *J = *8.4 Hz, 2H), 7.90 (d, J = 1.9 Hz, 1H), 10.85 (s, NH). ^13^C NMR (75 MHz, DMSO-*d*_6_, in ppm) δ = 14.3, 21.9, 33.3, 103.6, 106.0, 110.6, 113.0, 114.0, 121.4, 126.5, 128.1, 132.2, 134.0, 144.5, 149.6, 155.1, 157.2, 160.9, 162.8. HRMS (ESI): calcd. for C_21_H_16_BrN_2_O_2_S [M-H]^−^: 439.01463; found 439.01213.

### 7-(4-(4-Chlorophenyl)thiazol-2-ylamino)-4-propyl-2H-chromen-2-one (11c)

The title compound was obtained starting from the reaction of **7c** and **8c (**yellow solid, 65% yield, mp. 244–245 °C). ^1^H NMR (300 MHz, DMSO-*d*_6_, in ppm): δ = 0.95 (t, *J* = 7.3 Hz, 3H), 1.62 (m, 2H), 2.71 (t, *J* = 7.4 Hz, 2H), 6.12 (s, 1H), 7.46 (dd, *J* = 8.8, 2.0 Hz, 1H), 7.51 (d, *J* = 8.8 Hz, 2H), 7.52 (s, 1H), 7.76 (d, *J* = 8.8 Hz, 1H), 7.93 (d, *J* = 8.8 Hz, 2H), 7.94 (broad s, 1H), 10.84 (s, NH). ^13^C NMR (75 MHz, DMSO-*d*_6_, in ppm) δ = 14.2, 21.6, 33.3, 103.6, 105.9, 110.6, 113.0, 114.0, 126.5, 127.8, 129.3, 132.7, 133.6, 144.5, 149.6, 155.1, 157.2, 160.9, 162.8. HRMS (ESI): calcd. for C_21_H_16_ClN_2_O_2_S [M-H]^−^: 395.06265; found 395.06559.

### 7-(4-(3-Methoxyphenyl)thiazol-2-ylamino)-4-propyl-2H-chromen-2-one (11e)

The title compound was obtained starting from the reaction of **7c** and **8e (**brown solid, 85% yield, mp 200–203 °C). ^1^H NMR (300 MHz, DMSO-*d*_6_, in ppm): δ = 0.95 (t, *J* = 7.3 Hz, 3H), (m, 2H), 2.70 (t, *J* = 7.6 Hz, 2H), 3.78 (s, 3H), 6.11 (s, 1), 6.89 (dd, *J* = 8.1, 2.4 Hz, 1H, 7.35 (pseudo t, 1H, 7.45 (dd, *J* = 8.8, 2.0 Hz, 1H), 7.46 (m, 1H),7.47 (s, 1H), 7.50 (m, 1H, 7.76 (d, *J* = 8.8 Hz, 1H), 7.99 (d, *J* = 2.0 Hz, 1H), 10.82 (s, NH). ^13^C NMR (75 MHz, DMSO-*d*_6_, in ppm): δ = 14.2, 21.9, 33.3, 55.6,103.6, 105.4, 110.6, 112.0, 113.0, 114.0, 114.2, 118.6, 126.4, 130.4, 136.1, 144.6, 150.6, 155.2, 157.2, 160.1, 161.0, 162.5. HRMS (ESI): calcd. for C_22_H_19_N_2_O_3_S [M-H]^−^: 391.11219; found 391.11430.

### 7-(4-(3-Nitrophenyl)thiazol-2-ylamino)-4-propyl-2H-chromen-2-one (11f)

The title compound was obtained starting from the reaction of **7c** and **8f (**bright brown solid, 88% yield, mp 260–263 °C). ^1^H NMR (300 MHz, DMSO-*d*_6_, in ppm): δ = 0.95 (t, *J = *7.4 Hz, 3H), 1.63 (m, 2H), 2.72 (t, *J* = 7.6 Hz, 2H), 6.12 (s, 1H), 7.48 (dd, *J* = 8.8, 2.1 Hz, 1H), 7.73 (m, 1H), 7.76 (d, *J* = 8.8 Hz, 1H), 7.79 (s, 1H), 7.91 (d, *J* = 2.1 Hz, 1H), 8.14 (dd*, J* = 8.2, 1.8 Hz, 1H), 8.35 (d*, J* = 8.1 Hz, 1H), 8.63 (pseudo t, 1H), 10.90 (s, NH). ^13^C NMR (75 MHz, DMSO-*d*_6_, in ppm): δ = 14.2, 21.9, 33.3, 103.7, 107.8, 110.7, 113.2, 114.0, 120.5, 122.8, 126.5, 130.9, 132.3, 136.3, 144.4, 148.4, 148.9, 155.1, 157.1, 160.9, 163.1. HRMS (ESI): calcd. for C_21_H_16_N_3_O_4_S [M-H]^−^: 406.08670; found 406.08880.

### 7-(4-(3-Fluorophenyl)thiazol-2-ylamino)-4-propyl-2H-chromen-2-one (11g)

The title compound was obtained starting from the reaction of **7c** and **8g (**bright brown solid, 78% yield, mp 230–233 °C). ^1^H NMR (300 MHz, DMSO-*d*_6_, in ppm): δ = 0.94 (t, *J* = 7.3 Hz, 3H), 1.61 (m, 2H), 2.70 (t, *J* = 7.7 Hz, 2H), 6.10 (s, 1H), 7.14 (ddd, *J* = 8.5, 2.5 Hz, 1H), 7.47 (d, *J* = 8.7 Hz, 1H), 7.50 (m, 1H), 7.58 (s, 1H’), 7.70 (br s, 1H), 7.74 (d, *J = *8.7 Hz, 1H), 7.75 (d, *J* = 8.8 Hz, 1H), 7.91 (d, *J* = 2.0 Hz, 1H), 10.80 (s, NH). ^13^C NMR (75 MHz, DMSO-*d*_6_, in ppm): δ = 14.2, 21.9, 33.3, 103.5, 106.5, 110.6, 112.7 (d, ^2^*J*^C-F = ^22.8 Hz), 113.0, 114.0, 115.0 (d, ^2^*J*^C-F^ = 21.1 Hz), 121.9 (d, ^4^*J*^C-F^ = 0.6 Hz), 126.5), 131.3 (d, ^3^*J*^C-F^ = 8.2 Hz), 137.1 (d, ^3^*J*^C-F^ = 8.15 Hz), 144.4, 149.5, 155.1, 157.2, 161.0, 162.7, 163.1 (d, ^1^*J*^C-F^ = 242.7 Hz).

### 7-(4-(2-Nitrophenyl)thiazol-2-ylamino)-4-propyl-2H-chromen-2-one (11h)

The title compound was obtained starting from the reaction of **7c** and **8h (**bright brown solid, 85% yield, mp 199–200 °C). ^1^H NMR (300 MHz, DMSO-*d*_6_, in ppm): δ = 0.94 (t, *J* = 7.4 Hz, 3H), 1.61 (m, 2H), 2.70 (t, *J* = 7.6 Hz, 2H), 6.10 (s, 1H), 7.30 (d, *J* = 8.7 Hz, 1H), 7.45 (s, 1H), 7.57 (pseudo t, *J* = 7.7, 7.5 Hz, 1H), 7.67 (d, *J* = 8.7 Hz, 1H), 7.69 (m, 1H), 7.70 (s, 1H),7.83 (d, *J* = 7.9 Hz, 2H’), 10.79 (s, NH). ^13^C NMR (125 MHz, DMSO-*d*_6_, in ppm): δ = 14.2, 21.8, 33.2, 103.7, 109.0, 110.7, 113.1, 113.9, 124.1, 126.1, 128.0, 129.7, 130.5, 132.6, 144.2, 146.4, 149.4, 155.2, 157.0, 160.9, 162.6. HRMS (ESI): calcd. for C_21_H_16_N_3_O_4_S [M-H]^−^: 406.08670; found 406.08848.

### Cell culture

Human colorectal carcinoma cell lines (HCT116) and (HT-29) were obtained from the European Collection of Cell Cultures (ECACC, UK). The cell lines were cultured in Roswell Park Memorial Institute medium (RPMI-1640, Sigma-Aldrich-St. Louis, MO, USA) supplemented with 10% fetal bovine serum (Sigma-Aldrich-St. Louis, MO, USA) and 1% penicillin/streptomycin (Sigma-Aldrich- Louis, MO, USA). F180 cell line (Human Normal Fibroblast), was kindly given by professor Ekkehard Dikomey (University Cancer Center, Hamburg University, Hamburg, Germany) and were cultured in Dulbecco’s Modified Eagle’s medium (DMEM, Sigma-Aldrich-St. Louis, MO, USA) supplemented with 10% fetal bovine serum (Sigma-Aldrich-St. Louis, MO, USA) and 1% penicillin/streptomycin (Sigma-Aldrich- Louis, MO, USA). All cell incubations were done at 37^o^ C in a humidified atmosphere of 5% CO_2_.

### Cell viability analysis

Cell viability was assessed using, 3-(4,5-dimethylthiazol-2-yl)-2,5-diphenyltetrazolium bromide (MTT) assay as previously described^38^. In summary, cancer cell lines or F180 were seeded in complete medium at the density of 3 × 10^3^/well and 5 × 10^3^/well respectively in 96 well flat-bottom plates. After 24 h, cells were incubated with the candidate compounds at the indicated concentration for (24, 48 or 72 h). DMSO was used as vehicle control and 4-Hydroxycoumarin was purchased from (Sigma-Aldrich, St. Louis, MO, USA) and Cisplatin from Hospira UK Ltd. At the end of treatment, the media were aspirated from each well and incubated with 200 µL of MTT tetrazolium dye (Sigma-Aldrich, St. Louis, MO, USA) at a final concentration of 0.5 mg/ml for 2 h at 37^o^C. The reduced MTT crystals were solubilized in 200 µl/well of DMSO and the absorbance was measured at 570 nm using a microplate reader (Thermo-Scientific, Vantaa, Finland).

### Caspase 3/7 activity analysis

Caspase-3/7 activities in HT-29 and HCT116 cells treated with candidate compounds were measured using Caspase-Glo 3/7 assay kit (Promega, Madison, WI, USA) following the manufacturer’s instructions. In brief, 1 × 10^5^ Cells were plated into 6-well plates in triplicates. Following treatment with the candidate compounds, the media were aspirated, and the cells were washed with PBS and lysed in RIPA buffer. 50 µL of lysate and 50 µL of caspase substrate were added to a white opaque 96-well plate, and the luminescence was measured by Varioskan Flash multimode reader (Thermo-Scientific, Vantaa, Finland)

### Western blot

Following 72 h of treatment, cells were lysed and the extracted proteins were quantified using DC protein assay kit (Bio-Rad, Hercules, CA, USA) following the manufacturer’s instructions. Proteins were resolved on 8% or 12% SDS-poly-acrylamide gels, then transferred onto a nitrocellulose membrane using a semi-dry transfer cell. The membranes were blocked in TBS-T containing 5% non-fat milk for 1 h and incubated overnight at 4^o^C overnight with indicated primary antibodies (Cell Signaling Technologies, Beverly, MA, USA) and then incubated with goat anti-mouse or anti-rabbit secondary antibody (Cell Signaling Technologies, Beverly, MA, USA) for 1 h at room temperature. Detection was done using chemiluminescence substrate (Bio-Rad, Hercules, CA, USA). β-actin (Cell Signaling Technologies, Beverly, MA, USA) was used as a loading control.

The blots were captured with ChemiDoc Imaging System (Bio-Rad, Hercules, CA, USA) and bands were visualized with the Image Lab v5.2.1 software (Bio-Rad, Hercules, CA, USA).

### Cell cycle analysis

The cell cycle was assessed by flow cytometry, in which cells were treated at the indicated concentrations with the tested compounds for 24h^39^. Then cells were harvested, fixed with 70% ethanol, and stained with propidium iodide (Sigma-Aldrich, St. Louis, MO, USA), a DNA fluorescent binding dye. BD FACS Aria (Becton-Dickinson, Ann Arbor, MI, USA) was used to acquire the results and data were analyzed using FlowJo V.10 software (Ashland, OR, USA). DMSO was used as vehicle control and metformin (Sigma-Aldrich-St. Louis, MO, USA) as a positive control

### Glucose uptake assay

CRC cells (1 × 10^4^) were seeded into a 96-well plate overnight. Cells were treated with test compounds or vehicle (negative control) glucose-free RPMI-1640 medium for 16 h; then, the cells were incubated with 2 mM 2-DG for 10 minutes. Eventually, glucose uptake of cells was measured using Glucose Uptake-Glo Assay (Promega, Madison, WI, USA) according to the manufacturer’s instructions. Luminescence was measured post 2 h of incubation at room temperature using Varioskan Flash multimode reader (Thermo Scientific). Glucose uptake was normalized to the total protein content in the samples using DC protein assay kit (Bio-Rad, Hercules, CA, USA).

### Measurement of NADPH/NADP+ and ROS Levels

NADPH/NADP+ and ROS concentrations were measured using the NADP/NADPH-Glo Assay Kit (Promega, Madison, WI, USA) and ROS-Glo H2O2 Assay (Promega, Madison, WI, USA), respectively, based on the manufacturer instructions. In NADPH/NADP+ assay, CRC cells were seeded into a 96-well flat-bottomed plate and incubated overnight. On the following day, cells were treated with the testing compound or vehicle for 48 h. At the endpoint of treatment, the media were aspirated and replaced with 60 µl PBS/well, then cells were lysed in 60 µl base solution with 1% dodecyl (trimethyl) ammonium bromide (Sigma-Aldrich-St. Louis, MO, USA). Afterward, cell lysate was transferred into a white opaque 96-well plate to measure NADP+ and NADPH individually. Luminescence was measured after 30 minutes using Varioskan Flash multimode reader (Thermo Scientific). Whereas in ROS assay cells were plated in 96-well flat-bottomed plates in a total volume of 70 µl overnight. Later, cells were treated with different concentrations of compounds or the vehicle, followed by 20 µl of the H_2_O_2_ substrate solution for a total volume of 100 µl. After the 6 h incubation at 37 °C in a 5% CO_2_ incubator, 50 µl of each reaction mixture was transferred to a white opaque 96-well plate and mixed with 50 µl of the ROS-Glo detection solution. Luminescence was measured after 20 min incubation at room temperature.

### Statistical analysis

The data are expressed as mean ± SD, and the statistical analysis was performed using unpaired student’s t-test using Graphpad Prism V. 6.01 software. Differences were considered significant at p < 0.05.

## Supplementary information

Supplementary information

## Data Availability

The data generated and analyzed that support the scientific findings and claims of this study are presented in this published article.
