# Supplementary material for: Design and synthesis of new energy restriction mimetic agents: Potent anti-tumor activities of hybrid motifs of aminothiazoles and coumarins
Source: Sci Rep. 2020 Feb 19;10:2893. doi: 10.1038/s41598-020-59685-x (PMC7031302; doi:10.1038/s41598-020-59685-x)
Supplement: Supplementary file 1 — Supplementary information [file 41598_2020_59685_MOESM1_ESM.docx]

**Supporting Information**

**Design and synthesis of new energy restriction mimetic agents: Potent anti-tumor activities of hybrid motifs of aminothiazoles and coumarins**

Fatema Hersi ^a^_,_^b^, Hany A. Omar ^a,c,d,*^,Raed A. Al-Qawasmeh ^e^ ,Zainab Ahmad ^f^ ,Areej M. Jaber ^f^, Dana M. Zaher ^a,b^, Taleb H. Al-Tel ^a,c,*^

*^a^Sharjah Institute for Medical Research, University of Sharjah, Sharjah 27272, United Arab Emirates*

*^b^College of Medicine, University of Sharjah, Sharjah 27272, United Arab Emirates*

*^c^College of Pharmacy, University of Sharjah, Sharjah 27272, United Arab Emirates*

*^d^Department of Pharmacology, Faculty of Pharmacy, Beni-Suef University, Beni-Suef 62514, Egypt*

*^e^Department of Chemistry, Faculty of Science, University of Sharjah, Sharjah 27272, United Arab Emirates*

*^f^Department of Chemistry, Faculty of Science, The University of Jordan, Amman 11942, Jordan*

*Correspondence should be addressed to:

**Hany A. Omar, Ph.D**

Sharjah Institute for Medical Research, University of Sharjah, Sharjah 27272, United Arab Emirates. Email: [hanyomar@sharjah.ac.ae](mailto:hanyomar@sharjah.ac.ae)

**Taleb H. Al-Tel, Ph.D**

Sharjah Institute for Medical Research, University of Sharjah, Sharjah 27272, United Arab Emirates. Email: [taltal@sharjah.ac.ae](mailto:taltal@sharjah.ac.ae)

**Table of Contents**

**Synthesis of compounds S3**

**Biological Assays S20**

**^1^H- and ^13^C-NMR spectra of compounds S23**

**Full length WB gels S47**

**IC50 of the most potent compounds S62**

**The antiproliferative activity of most potent compounds at 24h S63**

**The antiproliferative activity of most potent compounds at 48h S64**

**Protein Quantitation Results S65**

**Synthesis of compounds**

# General chemistry. All reagents used were purchased from commercial suppliers without further purification. The reactions were carried out in oven-dried or flamed graduated vessels. Solvents were dried and purified by conventional methods prior use. All reactions were monitored using Merck aluminum plated pre-coated with silica gel PF254 and detected by visualization of the plate under UV lamp (λ =254 or 365). Column chromatography was performed using silica gel 60, 0.040-0.063 mm (230-400 mesh). ^1^H and ^13^C NMR spectra were recorded on a Bruker Avance DPX-300 HMz and DPX-500 HMz instruments. Splitting patterns are designated as s, singlet; d, doublet; dd, doublet of doublet; t, triplet; q, quartet; m, multiplet; br, broad. Chemical shifts (δ) are given in ppm with reference to TMS as internal standard. High-resolution mass spectra (HRMS) were measured by Electrospray Ionization (ESI) on a Bruker APEX-IV instrument. The samples were dissolved in acetonitrile and infused using a syringe pump with a flow rate of 120 μL/min. External calibration was conducted using Arginine cluster in a mass range *m*/*z* 175-871. For all HRMS data, mass error: 0.00-0.50 mDa.

**General procedure for the preparing 7-Methoxycarbonylamino-4-substituted Coumarins 3 a-c.** A mixture of 10 mmol (1 eq) of of *m*-(N-methoxycarbonylamino) phenol **2** was added in portions to 15 ml of concentrated H_2_SO_4_ followed by dropwise addition of 11 mmol (1.1 eq) of appropriate β-Ketoester ester derivatives with starring and cooling (10-15 ˚C). The mixture was further stirred for 3 h, upon completion 40 ml of ice-water was added and stirred until crystals formed. The precipitate was filtrated, washed with water and methanol and dried to give the desired product, which was used in the next steps without further purification.

**7-Methoxycarbonylamino-4-Methylcoumarin** ***3a.*** The title compound was obtained starting from the reaction of **2** with ethyl acetoacetate as described above (white solid, 72% yield, mp 255-257 ˚C). ^1^H NMR (300 MHz, DMSO-*d*_6_, in ppm): δ = 2.47 (s, 3H), 3.67 (s, 3H), 6.18 (s, 1H), 7.35 (dd, *J* = 8.7, 1.8 Hz, 1H), 7.49 (d, *J* = 1.8 Hz, 1H), 7.63 (d, *J* = 8.7 Hz, 1H), 10.13 (s, NH). ^13^C NMR (75 MHz, DMSO-*d*_6_, in ppm) δ = 18.5, 52.5, 104.9, 112.4, 114.7, 114.8, 126.5, 143.3, 153.7, 154.3, 154.3, 160.5. HRMS (ESI): calcd. for C_12_H_10_NO_4_ [M-H]^˗^: 232.06153; found 232.06277.

**7-Methoxycarbonylamino-4-Ethylcoumarins** *(****3b****)*. The title compound was obtained starting from the reaction of **2** with ethyl propioacetate as described above (white solid, 60% yield, mp 192-194 ˚C). ^1^H NMR (300 MHz, DMSO-*d*_6_, in ppm): δ = 1.18 (t, *J* = 7.4 Hz, 3C), 2.74 (q, *J* = 7.4 Hz, 2C), 3.67 (s, 3H), 6.13 (s, 1H), 7.35 (dd, *J* = 8.7, 2.0 Hz, 1H), 7.51 (d, *J* = 2.0 Hz, 1H), 7.69 (d, *J* = 8.8 Hz, 1), 10.12 (s, NH). ^13^C NMR (75 MHz, DMSO-*d*_6_, in ppm): δ = 12.7, 24.4, 52.5, 105.1, 111.3, 114.0, 114.8, 126.1, 143.1, 154.4, 154.5, 158.5, 160.8. HRMS (ESI): calcd. for C_13_H_12_NO_4_ [M-H]^˗^ : 246.07718; found 246.07859.

**7-Methoxycarbonylamino-4-Propylcoumarins** *(****3c****).* The title compound was obtained starting from the reaction of **2** with ethyl butyrylacetate as described above (white solid, 89% yield, mp 187-188 ˚C). ^1^H NMR (300 MHz, DMSO-*d*_6_, in ppm): δ = 0.93 (t, *J* = 7.3 Hz, 3H), 1.59 (m, 2H), 2.69 (t, *J* = 7.5 Hz, 2H), 3.67 (s, 3H), 6.14 (s, 1H), 7.35 (d, *J* = 8.8 Hz, 1H), 7.52 (br s, 1H), 7.71 (d, *J* = 8.8 Hz, 1H), 10.13 (s, NH). ^13^C NMR (75 MHz, DMSO-*d*_6_, in ppm): δ = 14.2, 21.8, 33.2, 52.6, 105.1, 111.5, 114.1, 114.8, 126.3, 143.2, 154.3, 154.6, 157.0, 160.7. HRMS (ESI): calcd. for C_14_H_14_NO_4_ [M-H]^˗^: 260.09283; found 260.09396.

**General procedure for preparing 7-Amino-4-Subsitutied Coumarins** ***4a-c****.* A suspension of 15 mmole of **(3a-c)** in 15 mL of 45% of KOH was stirred at 80-90 ˚C for 3 h until completion. The mixture was cooled and diluted with 50 mL water, the solution was acidified with concentrated HCl to 7-8 pH with stirring and cooling until crystallization occurred. The precipitate was filtrated, washed with water and ether then air dried.

**7-Amino-4-methylcoumarin** ***4a***. The title compound was obtained starting from the reaction of **3a** (brown solid, 85% yield, mp 222-223˚C). ^1^H NMR (300 MHz, DMSO-*d*_6_, in ppm): ^1^H NMR (300 MHz, DMSO-*d*_6_, in ppm): δ = 2.26 (s, 3H), 5.86 (s, 1H), 6.06 (br s, NH***_2_***), 6.36 (d, *J* = 2.0 Hz, 1H), 6.52 (dd, *J* = 8.6, 2.0 Hz, 1H), 7.36 (d, *J* = 8.6 Hz, 1H). ^13^C NMR (75 MHz, DMSO-d_6_, in ppm) δ = 18.5, 99.0, 108.0, 109.3, 111.7, 126.7, 153.6, 154.3, 156.0, 161.2. HRMS (ESI): calcd. for C_10_H_9_NO_2_Na [M+Na]^+^: 198.05255; found 198.05240.

**7-Amino-4-ethylcoumarin** (**4b**). The title compound was obtained starting from the reaction of 3b (brown solid, 50% yield, mp 223-224 ˚C). ^1^H NMR (300 MHz, DMSO-*d*_6_, in ppm): δ = 1.15 (t, *J* = 7.5 Hz, 3C), 2.59 (q, *J* = 7.5 Hz, 2H), 5.82 (s, 1H), 6.05 (br s, N***H_2_***), 6.37 (d, *J* = 2.1 Hz, 1H), 6.52 (dd, *J* = 8.7, 2.1 Hz, 1H), 7.41 (d, *J* = 8.7 Hz, 1H). ^13^C NMR (75 MHz, DMSO-*d_6_*, in ppm) δ = 13.2, 24.5, 99.2, 106.1, 108.4, 111.8, 126.3, 153.4, 156.1, 159.3, 161.5. HRMS (ESI): calcd. for C_12_H_12_NO_2_ [M+ H]^+^: 190.08626; found 190.08635

**7-Amino-4-propylcoumarin** (***4c*)**. The title compound was obtained starting from the reaction of **3c** (brown solid, 62.5% yield, mp 203-204 ˚C). ^1^H NMR (300 MHz, DMSO-*d*_6_, in ppm): δ = 0.91 (t, *J* = 7.3 Hz, 3H), 1.56 (m, 2H), 2.59 (t, *J* = 7.6 Hz, 2H), 5.82 (s, 1H), 6.05 (br s, N***H_2_***), 6.37 (d, *J* = 2.1 Hz, 1H), 6.52 (dd, *J* = 8.7, 2.1 Hz, 1H), 7.40 (d, *J* = 8.7 Hz, 1H). ^13^C NMR (75 MHz, DMSO-d_6_, in ppm) δ = 14.2, 22.1, 33.3, 99.2, 107.0, 108.6, 111.7, 126.4, 153.4, 156.2, 157.7, 161.3. HRMS (ESI): calcd. for C_12_H_13_NO_2_Na [M+Na]^+^: 226.08385; found 226.08362.

**General procedure for the synthesis of N-(4-Subsituted -2-Oxo-2H-Chromen-7-ylcarbamothioyl)benzamide derivative 6a-c**. Phenyl isothiocyanate (1.1 mmol, 1.1 eq) was added drop wise to a stirred solution of **4a-c** (1 mmol, 1eq) in 10 mL acetone at room temperature. The mixture was refluxed for about 3-4 h. The mixture was cooled, diluted with ice-water then stirred for additional 30 min. The precipitate was filtered washed with water, and air dried to produce the corresponding products **6a-c**.

**N-(4-Methyl-2-oxo-2H-chromen-7-ylcarbamothioyl)benzamide** **6a**. The title compound was obtained starting from **4a** (bright yellow solid, 95% yield, mp 201-203˚C). ^1^H NMR (300 MHz, DMSO-*d*_6_, in ppm): δ = 2.41 (s, 3H), 6.35 (s, 1H), 7.51 (pseudo t, 2H), 7.60 (dd, *J* = 8.6, 2.0 Hz, 1H), 7.62 (t, *J* = 7.4 Hz, 1H), 7.76 (d, *J* = 8.6 Hz, 1H), 7.95 (d, *J* = 7.2 Hz, 2H), 8.1 (s, 1H), 11.66 (s, NH), 12.79 (s, Ar-NH). ^13^C NMR (125 MHz, DMSO-d_6_, in ppm) δ = 18.5, 111.1, 114.2, 117.9, 120.3, 126.1, 129.0, 129.2, 132.5, 133.7, 141.5, 153.3, 153.5, 160.2, 168.7, 179.5. HRMS (ESI): calcd. for C_18_H_13_N_2_O_3_S [M-H]^˗^ : 337.06524; found 337.06745.

**N-(4-Ethyl-2-oxo-2H-cromen-7-ylcarbamothioyl)benzamide** (**6b**). The title compound was obtained starting from **4b (**bright yellow solid, 54% yield, mp 197-200˚C). ^1^H NMR (300 MHz, DMSO-*d*_6_, in ppm): δ = 1.21 (t, *J* = 7.4 Hz, 3H), 2.81 (q, *J* = 7.4 Hz, 2H), 6.28 (s, 1H), 7.51 (pseudo t, 2H), 7.60 (dd, *J* = 6.7, 2.2 Hz, 1H), 7.64 (t, *J* = 7.7 Hz, 1H), 7.81 (d, *J* = 8.7 Hz, 1H), 7.95 (d, *J* = 7.4 Hz, 2H), 8.08 (d, *J* = 2.2 Hz, 1H), 11.68 (s, NH), 12.78(s, Ar-NH). ^13^C NMR (75 MHz, DMSO-*d*_6_, in ppm) δ = 12.7, 24.5, 111.3, 112.3, 117.1, 120.4, 125.7, 129.0, 129.3, 132.5), 133.8, 141.5, 153.6, 158.2, 160.5, 168.7, 179.6. HRMS (ESI): calcd. for C_19_H_15_N_2_O_3_S [M-H]^˗^ : 351.08089; found 351.08330.

**N-(4-Propyl-2-oxo-2H-chromen-7-ylcarbamothioyl)benzamide** (**6c**). The title compound was obtained starting from **4a** (bright yellow solid, 97% yield, mp 184-186 ˚C). ^1^H NMR (300 MHz, DMSO-*d*_6_, ppm): δ = 0.96 (t, *J* = 7.3 Hz, 3H), 1.47 (m, 2H), 2.76 (t, *J* = 7.7 Hz, 2H), 6.29 (s, 1H), 7.50 (pseudo t, 2H), 7.60 (m, 1H), 7.63 (m, 1H), 7.84 (d, *J* = 8.7 Hz, 1H), 7.94 (dd, *J* = 5.2, 1.5 Hz, 2H), 8.07 (d, *J* = 1.8 Hz, 1H), 11.67(s, NH), 12.77(s, Ar-NH). ^13^C NMR (75 MHz, DMSO-*d*_6_, in ppm): δ = 14.2, 21.7, 33.2, 111.3, 113.3, 117.1, 120.4, 125.9, 128.9, 132.6, 132.7, 133.8, 141.5, 153.6, 156.7, 160.4, 168.7, 179.5. HRMS (ESI): calcd. for C_20_H_17_N_2_O_3_S [M-H]^˗^ : 365.09654; found 365.09877.

**General procedure for the synthesis of 7-Aminocoumarin thiourea derivatives** (**7a-c**). To a stirred solution of **6a-c** (1 mmol) in 20 mL methanol at room temperature was added 5 mL of aqueous (1N) NaOH. The mixture was refluxed for 3-4 h at 80˚C until hydrolysis was completed. The mixture was cooled and diluted with 20 mL ice-water, then the mixture was acidified to pH 7 using aqueous 1N HCl. The precipitate formed was filtered and dried. The products were further purified by column chromotography using gradient system of (*n*-hexane-ethyl acetate).

**1-(4-Methyl-2-oxo-2H-chromen-7-yl)thiourea** (**7a**). The title compound was obtained starting from **6a** (brown solid, 97% yield, mp 219-222 ˚C). ^1^H NMR (300 MHz, DMSO-*d*_6_, ppm): δ = 2.30 (s, 3H), 6.24 (s, 1H), 7.36 (dd, *J* = 8.7, 2.1 Hz, 1H), 7.66 (d, *J* = 8.7 Hz, 1H), 7.78 (d, *J* = 2.1 Hz, 1H), 10.08 (s, N***H***). ^13^C NMR (75 MHz, DMSO-*d*_6_, in ppm): δ = 18.5, 108.5, 112.9, 115.7, 118.2, 126.1, 143.5, 153.7, 153.8, 160.5, 181.6. HRMS (ESI): calcd. for C_11_H_9_N_2_O_2_S [M-H]^˗^ :233.03792; found 233.03536.

**1-(4-Ethyl-2-oxo-2H-chromen-7-yl)thiourea** (**7b**). The title compound was obtained starting from **6b** (brown solid, 71% Yield, mp 237-239˚C). ^1^H NMR (300 MHz, DMSO-*d*_6_, ppm): δ = 1.19 (t, *J* = 7.4 Hz, 3H), 2.77 (q, *J* = 7.4 Hz, 2H), 6.18 (s, 1H), 7.36 (d, *J* = 8.7 Hz, 1H), 7.71 (d, *J* = 8.7 Hz, 1H), 7.78 (br s, 1H), 10.06 (br s, N***H***). ^13^C NMR (75 MHz, DMSO-*d*_6_, in ppm): δ = 12.8, 24.5, 108.7, 111.0, 114.9, 118.2, 125.6, 143.3, 153.9, 158.4, 160.8, 181.6. HRMS (ESI): calcd. for C_12_H_11_N_2_O_2_S [M-H]^˗^ : 247.05467; found 247.05577.

**1-(2-Oxo-4-propyl-2H-chromen-7-yl)thiourea** (**7c**). The title compound was obtained starting from **6c** (brown solid, 97% yield, mp 234-235 ˚C). ^1^H NMR (300 MHz, DMSO-*d*_6_, in ppm): δ = 0.94 (t, *J* = 7.4 Hz, 3H), 1.60 (m, 2H), 2.71 (t, *J* = 7.4 Hz, 2H), 6.19 (s, 1H), 7.34 (dd, *J* = 8.7, 2.0 Hz, 1H), 7.71 (d, *J* = 8.7 Hz, 1H), 7.78 (d, *J* = 2.0 Hz, 1H), 10.06 (br s, NH). ^13^C NMR (75 MHz, DMSO-*d*_6_, in ppm): δ = 14.2, 21.8, 33.2, 108.8, 112.0, 115.0, 118.2, 125.8, 143.3, 154.1, 156.9, 160.6, 181.6. HRMS (ESI): calcd. for C_13_H_13_N_2_O_2_S [M-H]^˗^ : 261.06922; found 261.06972.

**General procedure for the synthesis of target compounds 9a-i, 10a-c, 10e-i, 11a-c, 11e-h**. The appropriate α-bromophenone **8a-i** (1.1 mmol) was added with stirring to a solution of a particular thiourea derivative **7a-c** (1 mmol) in 10 mL dry ethanol. The mixture was refluxed for 4 h. Upon completion, the mixture was cooled and poured into 20 mL of ice-water and stirred for additional 30 min. The pH of the solution was adjusted to 8 using 1N Na_2_CO_3_. The product was filtered off, washed with water and ether then purified by column chromatography (ethyl acetate- chloroform: 10-90%).

**4-Methyl-7-(4-phenylthiazol-2-ylamino)-2H-chromen-2-one** (**9a**)

The title compound was obtained starting from the reaction of **7a** and **8a** (bright Brown solid, 97% yield, mp 255-257 ˚C). ^1^H NMR (300 MHz, DMSO-*d*_6_, in ppm): δ = 2.53 (s, 3H), 6.16 (s, 1H), 7.31 (t, *J* = 7.3 Hz, 1H), 7.45 (s, 1H), 7.47 (m, 2H), 7,48 (d, *J* = 8.7 Hz, 1H), 7.70 (d, *J* = 8.7 Hz, 1H), 7.91 (d, *J* = 7.3 Hz, 2H), 7.99 (d, *J* = 2.0 Hz, 1H), 10.82 (s, NH). ^13^C NMR (75 MHz, DMSO-*d*_6_, in ppm): δ = 18.5, 103.3, 105.0, 111.5, 113.7, 113.9, 126.1, 126.7, 129.3, 129.6, 134.8, 144.7, 150.8, 153.9, 154.9, 162.8, 160.8. HRMS (ESI): calcd. for C_19_H_13_N_2_O_2_S [M-H]^˗^ : 333.07032; found 333.06892.

**7-(4-(4-Bromophenyl)thiazol-2-ylamino)-4-methyl-2H-chromen-2-one** (**9b**)

The title compound was obtained starting from the reaction of **7a** and **8b** (yellow solid, 97% yield, mp 292-293 ˚C). ^1^H NMR (300 MHz, DMSO-*d*_6_, in ppm): δ = 2.37 (s, 3H), 6.16 (s, 1H), 7.46 (dd, *J* = 8.7, 1.9 Hz, 1H), 7.53 (s, 1H), 7.64 (d, *J* = 8.5 Hz, 2H), 7.70 (d, *J* = 8.7 Hz, 1H), 7.86 (d, *J* = 8.5 Hz, 2H), 7.93 (d, *J* = 1.9 Hz, 1H), 10.84 (s, NH). ^13^C NMR (75 MHz, DMSO-*d*_6_, in ppm): δ = 18.5, 103.4, 106.0, 111.6, 113.8, 114.0, 121.4, 126.7, 128.1, 132.2, 134.0, 144.6, 149.6, 153.8, 154.9, 160.8, 162.9. HRMS (ESI): calcd. for C_19_H_12_BrN_2_O_2_S [M-H]^˗^ : 412.97875; found 412.98069.

**7-(4-(4-Chlorophenyl)thiazol-2-ylamino)-4-methyl-2H-chromen-2-one** (**9c)**

The title compound was obtained starting from the reaction of **7a** and **8c** (yellow solid 60% yield, mp 286-289˚C). ^1^H NMR (300 MHz, DMSO-*d*_6_, in ppm): δ = 2.37 (s, C***H_3_***), 6.17 (s, H-3), 7.47 (dd, J = 8.7, 2.1 Hz, H-6), 7.51 (d, J = 8.5 Hz, H-3'', H-5''), 7.52 (s, H-5'), 7.67 (d, J = 8.7 Hz, H-5), 7.92 (d, J = 8.5 Hz, H-2'',6''), 7.94 (s, H-8), 10.84 (s, N***H***). ^13^C NMR (75 MHz, DMSO-*d*_6_, in ppm): δ = 18.5, 103.3, 105.9, 111.6, 113.8, 114.0, 126.7, 127.8, 129.3, 132.8, 133.6, 144.6, 149.6, 153.8, 154.9, 160.8, 162.8. HRMS (ESI): calculated for C_19_H_12_ClN_2_O_2_S [M-H]^˗^: 367.03025; found 367.02719.

**7-(4-(4-Ethoxyphenyl)thiazol-2-ylamino)-4-methyl-2H-chromen-2-one** (**9d**)

The title compound was obtained starting from the reaction of **7a** and **8d** (brown solid, 86% yield, mp 222-224 ˚C). ^1^H NMR (300 MHz, DMSO-*d*_6_, in ppm): δ = 1.31 (t, *J* = 6.8 Hz, 3H), 2.37 (s, 3H), 4.12 (q, *J* = 6.8 Hz, 2H), 6.16 (s, 1H), 6.99 (d, *J* = 8.4 Hz, 2H), 7.26 (s, H-5'), 7.43 (d, *J* = 8.6 Hz, 1H), 7.69 (d, *J* = 8.6 Hz, 1H), ), 7.81 (d, *J* = 8.4 Hz, 2H), 8.00 (br s, H-8), 10.78 (s, NH). ^13^C NMR (75 MHz, DMSO-*d*_6_, in ppm): δ = 14.9, 18.5, 63.6, 102.7, 103.3, 111.4, 113.7, 113.9, 115.1, 126.6, 127.5, 132.5, 144.8, 150.7, 153.9, 154.9, 158.8, 160.9, 162.5. HRMS (ESI): calcd. for C_21_H_19_N_2_O_3_S [M+H]^+^ : 379.11109; found 379.11102.

**7-(4-(3-Methoxyphenyl)thiazol-2-ylamino)-4-methyl-2H-chromen-2-one** (**9e**)**.**

The title compound was obtained starting from the reaction of **7a** and **8e** (brown solid, 95% yield, mp 210-212 ˚C). ^1^H NMR (300 MHz, DMSO-*d*_6_, in ppm): δ = 2.46 (s, 3H), 3.86 (s, 3H), 6.16 (s, 1H), 6.87 (dd, *J* = 8.1, 2.4 Hz, 1H), 7.35 (pseudo t, 1H), 7.45 (d, *J* = 8.7 Hz, 1H), 7.47 (s, 1H),7.48 (s, 1H), 7.49 (m, 1H), 7.70 (d, *J* = 8.7 Hz, 1H), 7.99 (d, *J* = 1.9 Hz, 1H), 10.82 (s, NH). ^13^C NMR (75 MHz, DMSO-*d*_6_, in ppm): δ = 18.5, 55.6, 103.4, 105.4, 111.5, 112.0, 113.6, 113.7, 113.9, 118.6, 126.7, 130.4, 136.1, 144.7, 150.7, 153.8, 154.9, 160.1, 160.8, 162.5. HRMS (ESI): calcd. for C_20_H_15_N_2_O_3_S [M-H]^˗^ : 363.08089; found 36308302.

**4-Methyl-7-(4-(3-Nitrophenyl)thiazol-2-ylamino)-2H-chromen-2-one** (**9f).**

The title compound was obtained starting from the reaction of **7a** and **8f** (bright brown solid, 90% yield, mp 298-299 ˚C). ^1^H NMR (300 MHz, DMSO-*d*_6_, in ppm): δ = 2.37 (s, 3H), 6.18 (s, 1H), 7.46 (dd, *J* = 8.7, 1.9 Hz, 1H), 7.71 (dd, *J* = 7.9, 8.0 Hz, 1H), 7.75 (d, *J* = 8.7 Hz, 1H), 7.78 (s, 1H), 7.89 (d, *J* = 1.9 Hz, 1H), 8.14 (dd, *J* = 8.0, 1.9 Hz, 1H), 8.38 (d, *J* = 7.9 Hz, 1H), 8.67 (s, 1H), 10.89 (s, N*H*). ^13^C NMR (75 MHz, DMSO-*d*_6_, in ppm): δ = 18.5, 103.4, 107.8, 112.0, 113.2, 113.9, 120.5, 122.8, 126.7, 130.9, 132.3, 136.3, 144.5, 148.4, 153.7, 154.7, 154.9, 160.7, 160.7. HRMS (ESI): calcd. for C_19_H_12_N_3_O_4_S [M-H]^˗^ : 378.05540; found 378.05367.

**7-(4-(3-Fluorophenyl)thiazol-2-ylamino)-4-methyl-2H-chromen-2-one** (**9g**)

The title compound was obtained starting from the reaction of **7a** and **8g** (bright brown solid, 90% yield, mp 280-281 ˚C). ^1^H NMR (300 MHz, DMSO-*d*_6_, in ppm): δ = 2.48 (s, 3H), 2.73, 6.17 (s, 1H), 7.16 (dd, *J* = 8.6, 2.2 Hz, 1), 7.49 (m, 1H), 7.50 (m, 1H), 7.59 (s, 1H), 7.59 (d, *J*^2^ H-F = 2.0 Hz, H-2"), 7.69 (d, *J* = 8.2 Hz, 1H), 7.72 (d, *J* = 7.9 Hz, 1H), 7.93 (d, *J* = 2.1 Hz, 1H), 10.84 (s, NH). ^13^C NMR (75 MHz, DMSO-*d*_6_, in ppm): δ = 18.5, 103.4, 106.5, 111.6, 112.7 ( d, *J*^2 13^C-F = 22.9 Hz, 113.8, 114.0, 115.0 ( d, *J*^2^ ^13^C-F = 21.0 Hz), 122.2, 126.7, 131.2 ( d, *J*^3^ ^13^C-F = 8.6 Hz), 137.1 ( d, *J*^3^ ^13^C-F = 8.38 Hz), 144.6, 149.5, 153.8, 154.8, 160.8, 162.7, 163.1 (d, *J*^1^ ^13^C-F = 242.6 Hz). HRMS (ESI): calcd. for C_19_H_14_FN_2_O_2_S [M+H]^+^: 353.07545; found 353.07583.

**4-Methyl-7-(4-(2-nitrophenyl)thiazol-2-ylamino)-2H-chromen-2-one (9h**).

The title compound was obtained starting from the reaction of **7a** and **8h** (bright brown solid, 90% yield, mp dcomp. at 338˚C). ^1^H NMR (300 MHz, DMSO-*d*_6_, in ppm): δ = 2.36 (s, 3H), 6.16 (s, 1H), 7.31 (dd, *J* = 8.8, 1.9 Hz, 1H), 7.46 (s, 1H), 7.57 (pseudo t, 1), 7.63 (d, *J* = 8.8 Hz, 1H), 7.70 (m, 1H), 7.77 (br s, 1H), 7.82 (d, *J* = 7.9 Hz, 2H), 10.79 (s, NH). ^13^C NMR (75 MHz, DMSO-*d*_6_, in ppm): δ = 18.5, 103.5, 109.1, 111.6, 113.8, 113.9, 124.1, 126.7, 128, 129.7, 130.5, 132.6), 144.3, 146.4, 149.4, 153.7, 154.9, 160.7, 162.7. HRMS (ESI): calcd. for C_19_H_12_N_3_O_4_S [M-H]^˗^: 378.05540; found 378.05821.

**7-(4-(2-Methoxyphenyl)thiazol-2-ylamino)-4-methyl-2H-chromen-2-one** (**9i**)

The title compound was obtained starting from the reaction of **7a** and **8i** (bright brown solid, 88% yield, mp 224-226 ˚C). ^1^H NMR (300 MHz, DMSO-*d*_6_, in ppm): δ = 2.37 (s, 3H), 3.90 (s, 3H), 6.16 (s, 1H), 7.09 (m, 1H), 7.11 (d, *J* = 7.7 Hz, 1H), 7.31 (pseudo t, 1H), 7.48 (dd, *J* = 8.7, 2.0 Hz, 1H), 7.50 (s, 1H), 7.71 (d, *J* = 8.7 Hz, 1H), 7.95 (d, *J* = 2.0 Hz, 1H), 8.11 (dd, *J* = 7.6, 1.6 Hz, 1H), 10.75 (s, NH). ^13^C NMR (75 MHz, DMSO-*d*_6_, in ppm): δ = 18.5, 56.0, 103.2, 109.0, 111.4, 112.2, 113.6, 113.9, 121.1, 123.0, 126.7, 129.3, 129.5, 144.8, 146.7, 153.8, 154.9, 157.2, 160.8, 160.9. HRMS (ESI): calcd. for C_20_H_16_N_2_O_3_S [M-H]^˗^ : 363.08089; found 363.08277.

**4-Ethyl-7-(4-phenylthiazol-2-ylamino)-2H-chromen-2-one** (**10a**).

The title compound was obtained starting from the reaction of **7b** and **8a (**bright brown solid, 80% yield, mp 215-216 ˚C). ^1^H NMR (300 MHz, DMSO-*d*_6_, in ppm): δ = 1.21 (t, *J* = 7.4 Hz, 3HC), 2.78 (q, *J* = 7.4 Hz, 2H), 6.12 (s, 1H), 7.31(d, *J* = 7.0 Hz, 1H), 7.31 (t, *J* = 7.31, 1H), 7.43 (m, 2H), 7.44 (s, 1H), 7.46 (d, *J* = 8.8 Hz, 1H), 7.76 (d, *J* = 8.8 Hz, 1H), 7.93 (d, *J* = 7.9 Hz, 2H), 7.99 (br s, 1H), 10.82 (s, NH). ^13^C NMR (75 MHz, DMSO-*d*_6_, in ppm): δ = 12.9, 24.5, 103.5, 105.0, 109.6, 112.9, 114.0, 126.2, 126.3, 128.3, 129.3, 134.8, 144.6, 150.8, 155.0, 158.7, 161.1, 162.6. HRMS (ESI): calcd. for C_20_H_15_N_2_O_2_S [M-H]^˗^ : 347.08597; found 347.08669.

**7-(4-(4-Bromophenyl)thiazol-2-ylamino)-4-ethyl-2H-chromen-2-one** (**10b**).

The title compound was obtained starting from the reaction of **7b** and **8b (**yellow solid, 98% yield, mp 263-265 ˚C). ^1^H NMR (300 MHz, DMSO-*d*_6_, in ppm): δ = 1.21 (t, *J* = 7.4 Hz, 3H), 2.79 (q, *J* = 7.4 Hz, 2H), 6.12 (s, 1H), 7.46 (dd, *J* = 8.8, 2.1 Hz, 1H), 7.54 (s, 1H), 7.64 (d, *J* = 8.5 Hz, 2H), 7.75 (d, *J* = 8.8 Hz, 1H), 7.86 (d, *J* = 8.5 Hz, 2H), 7.93 (d, *J* = 2.1 Hz, 1H), 10.84 (s, NH). ^13^C NMR (75 MHz, DMSO-*d*_6_, in ppm): δ = 12.9, 24.5, 103.5, 106.0, 109.7, 113.0, 114.0, 121.4, 126.3, 128.1, 132.2, 134.0, 144.5, 149.6, 155.0, 158.6, 161.1, 162.8. HRMS (ESI) m/z, calculated for C_20_H_14_BrN_2_O_2_S [M-H]^˗^ = 426.99440, found 426.99681.

**7-(4-(4-Chlorophenyl)thiazol-2-ylamino)-4-ethyl-2H-chromen-2-one** (**10c**)

The title compound was obtained starting from the reaction of **7b** and **8c (**yellow solid, 97% yield, mp 258-260 ˚C. ^1^H NMR (300 MHz, DMSO-*d*_6_, in ppm): δ = 1.24 (t, *J* = 7.2 Hz, 3H), 2.80 (q, *J* = 7.2 Hz, 2H), 6.14 (s, 1H), 7.50 (d, *J* = 8.5 Hz, 1H), 7.54 (d, *J* = 8.2 Hz, 2H), 7.55 (s, 1H), 7.77 (d, *J* = 8.5 Hz, 1H), 7.96 (d, *J* = 8.2 Hz, 2H), 7.97 (s, 1H), 10.85 (s, N*H*). ^13^C NMR (75 MHz, DMSO-*d*_6_, in ppm): δ = 12.8, 24.4, 103.5, 105.8, 109.6, 112.9, 114.0, 126.2, 127.8, 129.3, 132.7, 133.6, 144.5, 149.6, 154.8, 158.6, 161.0, 162.8. HRMS (ESI): calcd. for C_20_H_14_ClN_2_O_2_S [M-H]^˗^ : 381.04700; found 381.04484.

**4-Ethyl-7-(4-(3-methoxyphenyl)thiazol-2-ylamino)-2H-chromen-2-one** (**10e)**

The title compound was obtained starting from the reaction of **7b** and **8e (**brown solid, 80% yield, mp 215-217 ˚C). ^1^H NMR (300 MHz, DMSO-*d*_6_, in ppm): δ = 1.20 (t, *J* = 7.4 Hz, 3H), 2.76 (q, *J* = 7.4 Hz, 2H), 3.80 (s, 3H), 6.10 (s, 1H), 6.90 (d, *J* = 8.0 Hz, 1H), 7.35 (pseudo t, 1H),7.45 (d, *J* = 8.8 Hz, 1H), 7.46 (s, 1H), 7.48 (s, 1H), 7.49 (d, *J* = 7.7 Hz, 1H), 7.74 (d, *J* = 8.8 Hz, 1H), 8.00 (br s, 1H), 10.81 (s, NH). ^13^C NMR (75 MHz, DMSO-*d*_6_, in ppm): δ = 12.8, 24.4, 55.6, 103.5, 105.3, 109.6, 112.0, 112.9, 113.6, 113.5, 118.6, 126.2, 130.3, 136.1, 144.6, 150.6, 155.0, 158.6, 160.1, 161.0, 162.4. HRMS (ESI): calcd. for C_21_H_17_N_2_O_3_S [M-H]^˗^ : 377.09654; found 377.09832.

**4-Ethyl-7-(4-(3-nitrophenyl)thiazol-2-ylamino)-2H-chromen-2-one** (**10f**).

The title compound was obtained starting from the reaction of **7b** and **8f (**bright brown solid, 86% yield, mp 303-304 ˚C). ^1^H NMR (300 MHz, DMSO-*d*_6_, in ppm): δ = 1.20 (t, *J* = 7.3 Hz, 3H), 2.76 (q, *J* = 7.3 Hz, 2H), 6.10 (s, 1H), 7.45 (d, *J* = 7.4 Hz, 1H), 7.72 (m, 1H), 7.74 (d, *J* = 7.4 Hz, 1H), 7.77 (s, 1H), 7.89 (s, 1H), 8.14 (d, *J* = 8.1 Hz, 1H), 8.33 (d, *J* = 7.8 Hz, 1H), 8.66 (s, 1H), 10.87 (s, NH). ^13^C NMR (75 MHz, DMSO-*d*_6_, in ppm): δ = 12.8, 24.5, 103.6, 107.7, 109.7, 113.1, 114.0, 120.5, 122.8, 126.2, 130.9, 132.3, 136.3, 144.3, 148.4, 148.9, 154.5, 158.5, 161.0, 163.1. HRMS (ESI): calcd. for C_20_H_14_N_3_O_4_S [M-H]^˗^ : 392.06995; found 392.06959.

**4-Ethyl-7-(4-(3-fluorophenyl)thiazol-2-ylamino)-2H-chromen-2-one** (**10g**).

The title compound was obtained starting from the reaction of **7b** and **8g** bright brown solid, 81% yield, mp 245-247 ˚C). ^1^H NMR (300 MHz, DMSO-*d*_6_, in ppm): δ = 1.18 (t, *J* = 7.2 Hz, 3H), 2.73 (q, *J* = 7.2 Hz, 2H), 6.07 (s, 1H), 7.12 (pseudo t, 1H), 7.45 (d, *J* = 9.2 Hz, 1H), 7.47 (m, 1H), 7.54 (s, 1H), 7.67 (d, *J*^2^ H-F = 2.0 Hz, 1H), 7.70 (d, *J* = 9.2 Hz, 1H), 7.74 (d, *J* = 7.5 Hz, 1H), 7.90 (broad s, 1H), 10.80 (s, NH). ^13^C NMR (75 MHz, DMSO-*d*_6_, in ppm): δ = 12.7, 24.4, 103.5, 106.4, 109.6, 112.7 (d, *J*^2^ ^13^C-F = 22.9 Hz), 112.9, 113.9, 115.0 ( d, *J*^2 13^C-F = 21.0 Hz), 122.1, 126.1, 131.2 ( d, *J*^3^ ^13^C-F = 8.4 Hz), 137.1 ( d, *J*^3^ ^13^C-F = 11.9 Hz), 144.4, 149.4, 154.9, 158.5, 161.0, 162.7, 163.1 (d, *J*^1^ ^13^C-F = 242.5 Hz). HRMS (ESI): m/z calcd. for C_20_H_16_FN_2_O_2_S [M+H]^+^ : 367.09110; found 367.09112.

**4-Ethyl-7-(4-(2-nitrophenyl)thiazol-2-ylamino)-2H-chromen-2-one** (**10h**).

The title compound was obtained starting from the reaction of **7b** and **8h (**bright brown solid, 81% yield, mp 261-263˚C). ^1^H NMR (300 MHz, DMSO-*d*_6_, in ppm): δ = 1.20 (t, J = 7.4 Hz, 3H), 2.77 (q, J = 7.4 Hz, 2H), 6.11 (s, 1H), 7.29 (dd, *J* = 8.7, 2.1 Hz, 1H), 7.46 (s, 1H), 7.57 (ddd, *J* = 6.5, 8.1, 1.3 Hz, 1H), 7.66 (d, *J* = 8.7 Hz, 1H), 7.68 (m, 1H),7.72 (d, *J* = 2.1 Hz, 1H), 7.83 (d, *J* = 8.1 Hz, 2H), 10.81 (s, N*H*). ^13^C NMR (75 MHz, DMSO-*d*_6_, in ppm): δ = 12.8, 28.9, 103.7, 109.1, 109.8, 113.1, 113.9, 124.1, 126.0, 128.0, 129.7, 130.5, 132.6, 144.2, 146.4, 149.4, 155.1, 158.5, 161.1, 162.7. HRMS (ESI): calcd. for C_20_H_14_N_3_O_4_S [M-H]^˗^ : 392.07276; found 392.07276.

**4-Ethyl-7-(4-(2-methoxyphenyl)thiazol-2-ylamino)-2H-chromen-2-one** (**10i**).

The title compound was obtained starting from the reaction of **7b** and **8i** (bright brown solid, 84% yield, mp 200-202˚C). ^1^H NMR (300 MHz, DMSO-*d*_6_, in ppm): δ = 1.21 (t, *J* = 7.4 Hz, 3H), 2.78 (q, J = 7.4 Hz, 2H), 3.90 (s, 3H), 6.12 (s, 1H), 7.08 (m, 1H), 7.11 (d, *J* = 7.7 Hz, 1H), 7.30 (pseudo t, 1H), 7.48 (d, *J* = 8.8 Hz, 1H), 7.50 (s, 1H), 7.75 (d, *J* = 8.8 Hz, 1H), 7.96 (d, *J* = 2.0 Hz, 1H), 8.11 (d, *J* = 6.4 Hz, 1H), 10.76 (s, NH). ^13^C NMR (75 MHz, DMSO-*d*_6_, in ppm): δ = 12.9, 24.5, 56.0, 103.4, 108.9, 109.6, 112.2, 112.9, 113.9, 121.1, 123.0, 126.3, 129.3, 129.5, 144.7, 146.8, 155.1, 157.2, 158.7, 160.9, 161.1. HRMS (ESI): calcd. for C_21_H_17_N_2_O_3_S [M-H]^˗^ : 377.09654; found 377.09654.

**7-(4-Phenylthiazol-2-ylamino)-4-propyl-2H-chromen-2-one** (**11a**).

The title compound was obtained starting from the reaction of **7c** and **8a (**bright brown solid, 71% yield, mp 230-232 ˚C). ^1^H NMR (500 MHz, DMSO-*d*_6_, in ppm): δ = 0.94 (t, *J* = 7.0 Hz, 3H), 1.62 (m, 2H), 2.71 (t, *J* = 7.0Hz, 2H), 6.11 (s, 1H), 7.31 (t, *J* = 6.9 Hz, 1H), 7.45 (s, 1H), 7.46 (m, 2H), 7.48 (d, *J* = 8.6, Hz, 1H), 7.75 (d, *J* = 8.6 Hz,1 H), 7.91 (d, *J* = 7.3 Hz, 2H), 8.0 (s, 1H),10.2 (s, NH). ^13^C NMR (125 MHz, DMSO-*d*_6_, in ppm) δ = 14.2, 21.9, 33.3, 103.7, 105.0, 110.7, 113.0, 114.0, 126.1, 126.3, 128.3, 129.2, 134.8, 144.6, 150.8, 155.1, 157.2, 160.9, 162.6. HRMS (ESI): calcd. for C_21_H_17_N_2_O_2_S [M-H]^˗^ : 361.10162; found 361.09916.

**7-(4-(4-Bromophenyl)thiazol-2-ylamino)-4-propyl-2H-chromen-2-one** (**11b**).

The title compound was obtained starting from the reaction of **7c** and **8a (**yellow solid, 95% yield, mp 290-293 ˚C). ^1^H NMR (300 MHz, DMSO-*d*_6_, in ppm): δ = 0.95 (t, *J* = 7.3 Hz, 3H), 1.63 (m, 2H), 2.71 (t, *J* = 7.4 Hz, 2H), 6.11 (s, 1H), 7.48 (dd, *J* = 8.8, 1.9 Hz, 1H), 7.53 (s, 1H), 7.63 (d, *J* = 8.4 Hz, 2H), 7.75 (d, *J* = 8.8 Hz, 1H), 7.85 (d, *J* = 8.4 Hz, 2H), 7.90 (d, J = 1.9 Hz, 1H), 10.85 (s, NH). ^13^C NMR (75 MHz, DMSO-*d*_6_, in ppm) δ = 14.3, 21.9, 33.3, 103.6, 106.0, 110.6, 113.0, 114.0, 121.4, 126.5, 128.1, 132.2, 134.0, 144.5, 149.6, 155.1, 157.2, 160.9, 162.8. HRMS (ESI): calcd. for C_21_H_16_BrN_2_O_2_S [M-H]^˗ :^ 439.01463; found 439.01213.

**7-(4-(4-Chlorophenyl)thiazol-2-ylamino)-4-propyl-2H-chromen-2-one** (**11c).**

The title compound was obtained starting from the reaction of **7c** and **8c (**yellow solid, 65% yield, mp. 244-245 ˚C). ^1^H NMR (300 MHz, DMSO-*d*_6_, in ppm): δ = 0.95 (t, *J* = 7.3 Hz, 3H), 1.62 (m, 2H), 2.71 (t, *J* = 7.4 Hz, 2H), 6.12 (s, 1H), 7.46 (dd, *J* = 8.8, 2.0 Hz, 1H), 7.51 (d, *J* = 8.8 Hz, 2H), 7.52 (s, 1H), 7.76 (d, *J* = 8.8 Hz, 1H), 7.93 (d, *J* = 8.8 Hz, 2H), 7.94 (broad s, 1H), 10.84 (s, NH). ^13^C NMR (75 MHz, DMSO-*d*_6_, in ppm) δ = 14.2, 21.6, 33.3, 103.6, 105.9, 110.6, 113.0, 114.0, 126.5, 127.8, 129.3, 132.7, 133.6, 144.5, 149.6, 155.1, 157.2, 160.9, 162.8. HRMS (ESI): calcd. for C_21_H_16_ClN_2_O_2_S [M-H]^˗^: 395.06265; found 395.06559.

**7-(4-(3-Methoxyphenyl)thiazol-2-ylamino)-4-propyl-2H-chromen-2-one** (**11e**).

The title compound was obtained starting from the reaction of **7c** and **8e (**brown solid, 85% yield, mp 200-203 ˚C). ^1^H NMR (300 MHz, DMSO-*d*_6_, in ppm): δ = 0.95 (t, *J* = 7.3 Hz, 3H), (m, 2H), 2.70 (t, *J* = 7.6 Hz, 2H), 3.78 (s, 3H), 6.11 (s, 1), 6.89 (dd, *J* = 8.1, 2.4 Hz, 1H, 7.35 (pseudo t, 1H, 7.45 (dd, *J* = 8.8, 2.0 Hz, 1H), 7.46 (m, 1H),7.47 (s, 1H), 7.50 (m, 1H, 7.76 (d, *J* = 8.8 Hz, 1H), 7.99 (d, *J* = 2.0 Hz, 1H), 10.82 (s, NH). ^13^C NMR (75 MHz, DMSO-*d*_6_, in ppm): δ = 14.2, 21.9, 33.3, 55.6,103.6, 105.4, 110.6, 112.0, 113.0, 114.0, 114.2, 118.6, 126.4, 130.4, 136.1, 144.6, 150.6, 155.2, 157.2, 160.1, 161.0, 162.5. HRMS (ESI): calcd. for C_22_H_19_N_2_O_3_S [M-H]^˗^: 391.11219; found 391.11430.

**7-(4-(3-Nitrophenyl)thiazol-2-ylamino)-4-propyl-2H-chromen-2-one** (**11f**).

The title compound was obtained starting from the reaction of **7c** and **8f (**bright brown solid, 88% yield, mp 260-263 ˚C). ^1^H NMR (300 MHz, DMSO-*d*_6_, in ppm): δ = 0.95 (t, *J* = 7.4 Hz, 3H), 1.63 (m, 2H), 2.72 (t, *J* =7.6 Hz, 2H), 6.12 (s, 1H), 7.48 (dd, *J* = 8.8, 2.1 Hz, 1H), 7.73 (m, 1H), 7.76 (d, *J* = 8.8 Hz, 1H), 7.79 (s, 1H), 7.91 (d, *J* = 2.1 Hz, 1H), 8.14 (dd*, J* = 8.2, 1.8 Hz, 1H), 8.35 (d*, J* = 8.1 Hz, 1H), 8.63 (pseudo t, 1H), 10.90 (s, NH). ^13^C NMR (75 MHz, DMSO-*d*_6_, in ppm): δ = 14.2, 21.9, 33.3, 103.7, 107.8, 110.7, 113.2, 114.0, 120.5, 122.8, 126.5, 130.9, 132.3, 136.3, 144.4, 148.4, 148.9, 155.1, 157.1, 160.9, 163.1. HRMS (ESI): calcd. for C_21_H_16_N_3_O_4_S [M-H]^˗:^ 406.08670; found 406.08880.

**7-(4-(3-Fluorophenyl)thiazol-2-ylamino)-4-propyl-2H-chromen-2-one** (**11g)**

The title compound was obtained starting from the reaction of **7c** and **8g (**bright brown solid, 78% yield, mp 230-233 ˚C). ^1^H NMR (300 MHz, DMSO-*d*_6_, in ppm): δ = 0.94 (t, *J* = 7.3 Hz, 3H),1.61 (m, 2H), 2.70 (t, *J* = 7.7 Hz, 2H), 6.10 (s, 1H), 7.14 (ddd, *J* = 8.5, 2.5 Hz, 1H), 7.47 (d, *J* = 8.7 Hz, 1H), 7.50 (m, 1H), 7.58 (s, 1H'), 7.70 (br s, 1H), 7.74 (d, *J* = 8.7 Hz, 1H), 7.75 (d, *J* = 8.8 Hz, 1H), 7.91 (d, *J* = 2.0 Hz, 1H), 10.80 (s, NH). ^13^C NMR (75 MHz, DMSO-*d*_6_, in ppm): δ = 14.2, 21.9, 33.3, 103.5, 106.5, 110.6, 112.7 ( d, *J*^2^ ^13^C-F = 22.8 Hz), 113.0, 114.0, 115.0 (d, *J*^2^ ^13^C-F = 21.1 Hz), 121.9 ( d, *J*^4^ ^13^C-F = 0.6 Hz), 126.5), 131.3 ( d, *J*^3^ ^13^C-F = 8.2 Hz), 137.1 ( d, *J*^3^ ^13^C-F = 8.15 Hz), 144.4, 149.5, 155.1, 157.2, 161.0, 162.7, 163.1 (d, *J*^1^ ^13^C-F = 242.7 Hz).

**7-(4-(2-Nitrophenyl)thiazol-2-ylamino)-4-propyl-2H-chromen-2-one** (**11h).**

The title compound was obtained starting from the reaction of **7c** and **8h (**bright brown solid, 85% yield, mp 199-200˚C). ^1^H NMR (300 MHz, DMSO-*d*_6_, in ppm): δ = 0.94 (t, *J* = 7.4 Hz, 3H), 1.61 (m, 2H), 2.70 (t, *J* = 7.6 Hz, 2H), 6.10 (s, 1H), 7.30 (d, *J* = 8.7 Hz, 1H), 7.45 (s, 1H), 7.57 (pseudo t , *J* = 7.7, 7.5 Hz, 1H), 7.67 (d, *J* =8.7 Hz, 1H), 7.69 (m, 1H), 7.70 (s, 1H),7.83 (d, *J* = 7.9 Hz, 2H'), 10.79 (s, NH). ^13^C NMR (125 MHz, DMSO-*d*_6_, in ppm): δ = 14.2, 21.8, 33.2, 103.7, 109.0, 110.7, 113.1, 113.9, 124.1, 126.1, 128.0, 129.7, 130.5, 132.6, 144.2, 146.4, 149.4, 155.2, 157.0, 160.9, 162.6. HRMS (ESI): calcd. for C_21_H_16_N_3_O_4_S [M-H]^˗^: 406.08670; found 406.08848.

**Biological Assays**

**Cell culture.** Human colorectal carcinoma cell lines (HCT116) and (HT-29) were obtained from the European Collection of Cell Cultures (ECACC, UK). The cell lines were cultured in Roswell Park Memorial Institute medium (RPMI-1640, Sigma-Aldrich-St. Louis, MO, USA) supplemented with 10% fetal bovine serum (Sigma-Aldrich-St. Louis, MO, USA) and 1% penicillin/streptomycin (Sigma-Aldrich- Louis, MO, USA). F180 (Human Normal Fibroblast), were kindly given by professor Ekkehard Dikomey (University Cancer Center, Hamburg University, Hamburg, Germany) and were cultured in Dulbecco’s Modified Eagle’s medium (DMEM, Sigma-Aldrich-St. Louis, MO, USA) supplemented with 10% fetal bovine serum (Sigma-Aldrich-St. Louis, MO, USA) and 1% penicillin/streptomycin (Sigma-Aldrich- Louis, MO, USA). All cell incubations were done at 37^o^ C in humidified atmosphere of 5 % CO_2_.

**Cell viability analysis.** For cell viability was assessment, 3-(4,5-dimethylthiazol-2-yl)-2,5-diphenyltetrazolium bromide (MTT) assay was used as previously described. In summary, cancer cell lines or F180 were seeded in complete medium at the density of 3x10^4^/well and 5x10^4^/well respectively in 96 well flat-bottom plates. After 24h, cells were incubated with the candidate compounds, OSU-CG5 and OSU-CG12 at indicated concentration for 72h. DMSO was used as a vehicle control. At the end of treatment, the media were aspirated from each well and incubated with 200 µL of MTT tetrazolium dye (Sigma-Aldrich, St. Louis, MO, USA) at final concentration of 0.5 mg/mL for 2h at 37^o^C. The reduced MTT dye was solubilized in 200 µl/well of DMSO and absorbance was measured at 570 nm using microplate reader (Thermo-Scientific, Vantaa, Finland).

**Caspase 3/7 activity analysis.** Caspase-3/7 activities in HT-29 and A549 cells treated with candidate compounds were measured using Caspase-Glo® 3/7 assay kit (Promega, Madison, WI, USA) following the manufacturer’s instructions. In brief, 1×10^5^ Cells were plated into 6-well plates in triplicates. Following treatment with the candidate compounds, the media was aspirated, and the cells were washed with PBS and were lysed in RIPA buffer.50 µL of lysate and caspase substrate was added to white opaque 96-well plate and the luminescence was measured by Varioskan Flash multimode reader (Thermo-Scientific, Vantaa, Finland)

**Western blot.** Following 72h of treatment, cells were lysed and quantified using DC protein assay kit (Bio-Rad, Hercules, CA, USA) following manufacturer’s instructions. Proteins were resolved on 8% or 12% SDS-poly-acrylamide gels, then transferred onto nitrocellulose membrane using semi-dry transfer cell. The membranes were blocked in TBS-T containing 5% non-fat milk for 1h and incubated overnight at 4^o^C overnight with indicated primary antibodies (Cell signaling Technologies, Beverly, MA, USA) and then incubated with secondary antibodies (Cell signaling Technologies, Beverly, MA, USA) for 1h at room temperature before the detection using chemi-luminescent substrate (Bio-Rad, Hercules, CA, USA). β-actin (Cell signaling Technologies, Beverly, MA, USA) was used as a loading control.

**Glucose uptake assay.** Glucose uptake was measured using Glucose Uptake Cell-Based Assay Kit (Cayman, Ann Arbor, Michigan) according to the manufacturer’s instructions. CRC cells (2 × 10^5)^ were seeded into a 6-well plate overnight. Cells were treated with test compounds or vehicle (control) in 1 mL glucose-free RPMI-1640 medium for 24h, then the cells were incubated with 150 µg/mL 2-NBDG for 1h at the end of treatment. Finally, cells were harvested and the 2-NBDG taken up by cells was measured using BD Accuri C6 (Becton-Dickinson, Ann Arbor, MI, USA) (excitation/emission= 485/535 nm). Data were obtained using FlowJo V.10 software (Ashland, OR, USA). DMSO was used as a vehicle control and 2-DG as positive control.

**Measurement of NADPH/NADP+ and ROS Levels.** NADPH/NADP+ and ROS concentrations were measured using the NADP/NADPH-Glo Assay Kit (Promega, Madison, WI, USA) ROS-Glo™ H₂O₂ Assay (Promega, Madison, WI, USA) respectively based on manufacturer instructions. In NADPH /NADP+ assay CRC cells were seeded into a 96-well flat-bottomed plate overnight and treated with testing compound or vehicle following day for 48h. At endpoint of treatment, media was aspirated and replaced with 60 µl PBS/well and cells were lysed in 60 µl base solution with 1 % dodecyl (trimethyl)azanium bromide (Sigma-Aldrich-St. Louis, MO, USA). Afterwards, cell lysate was transferred into a white opaque 96-well plate to measure NADP+ and NADPH individually. Luminescence was measured after 30 minutes using Varioskan Flash multimode reader (Thermo Scientific). Whereas in ROS assay cells were plated in 96-well flat-bottomed plates in a total volume of 70 µl overnight. Later, cells were treated with different concentration of compounds or their vehicle, followed by 20 µl of the H_2_O_2_ substrate solution for a total volume of 100 µl. After the 6h incubation at 37°C in a 5% CO_2_ incubator, 50 µl of each reaction mixture was transferred to a white opaque 96-well plate and mixed with 50 µl of the ROS-Glo detection solution. Luminescence was measured after 20 min incubation at room temperature.

**1H- and 13C-NMR spectra of compounds**


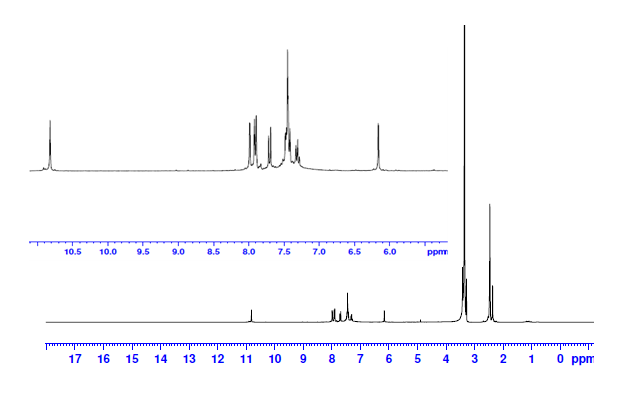


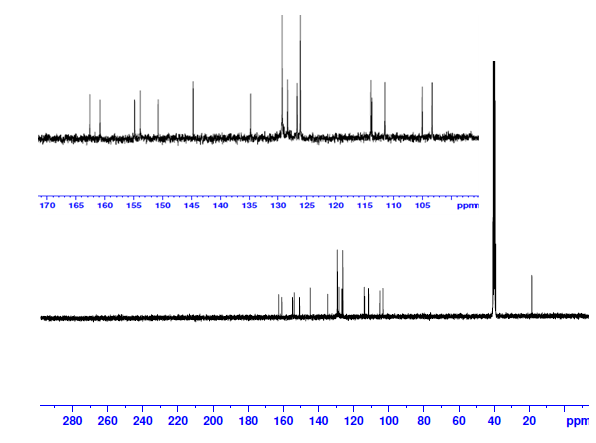


**Figure 1:** ^1^H and^13^C-NMR spectrum for compound **(9a)**

**
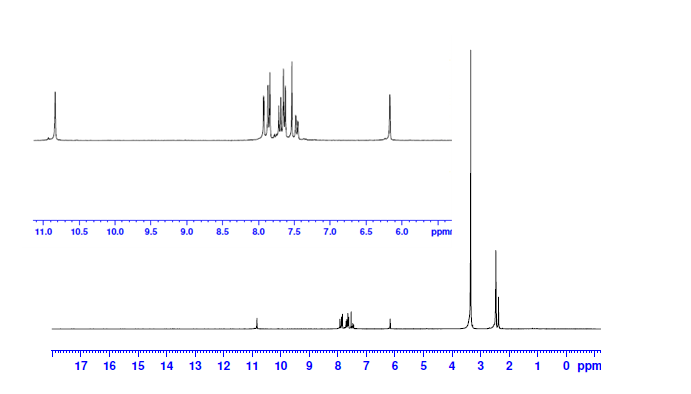
**


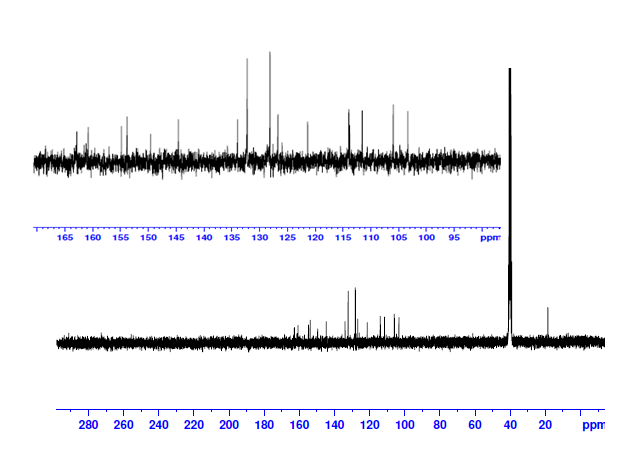


**Figure 2:** ^1^H and^13^C-NMR spectrum for compound **(9b)**

**
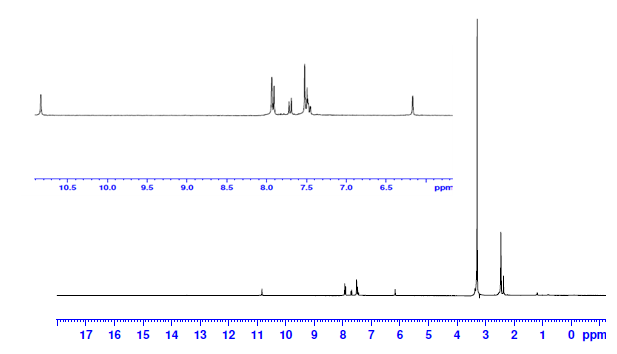
**

**
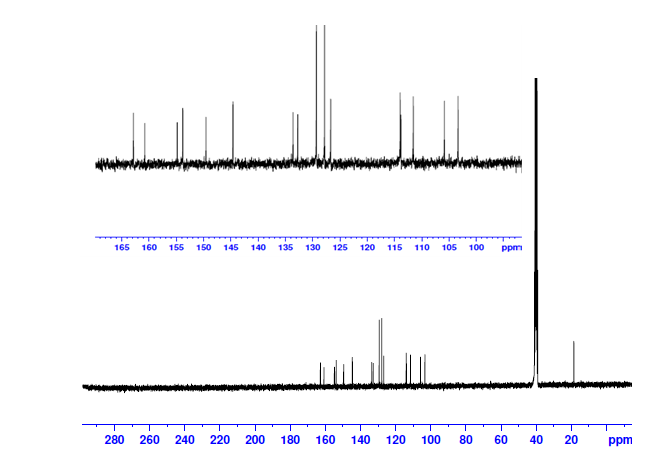
**

**Figure 3:** ^1^H and^13^C-NMR spectrum for compound **(9c)**

**
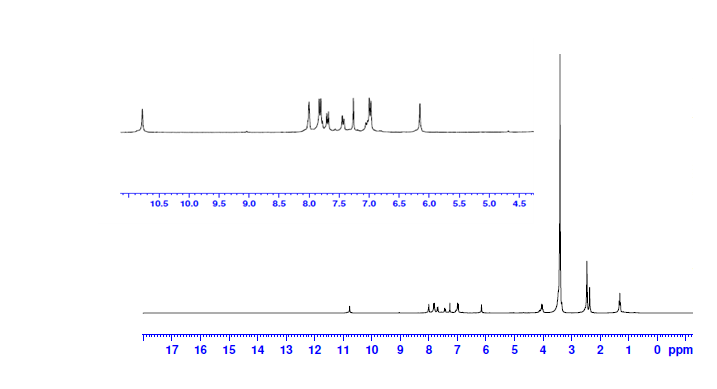
**

**
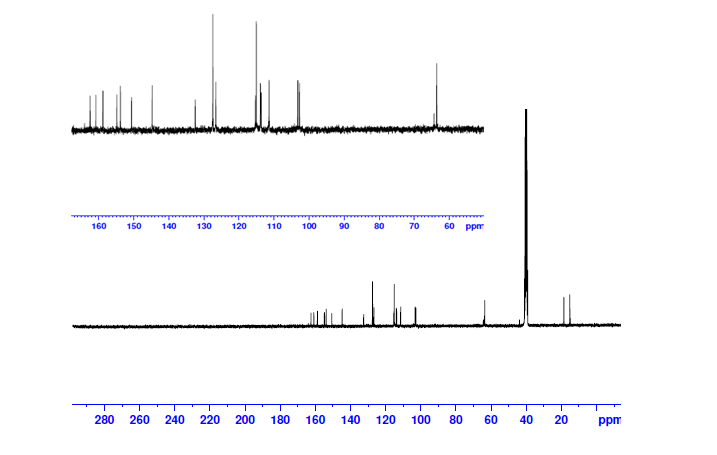
**

**Figure 4:** ^1^H and^13^C-NMR spectrum for compound **(9d)**

**
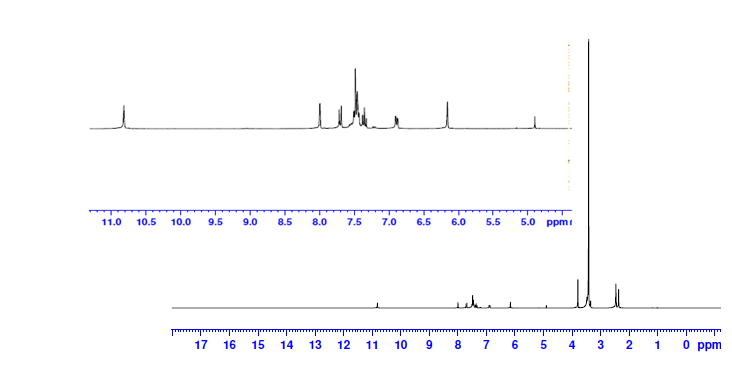
**

**
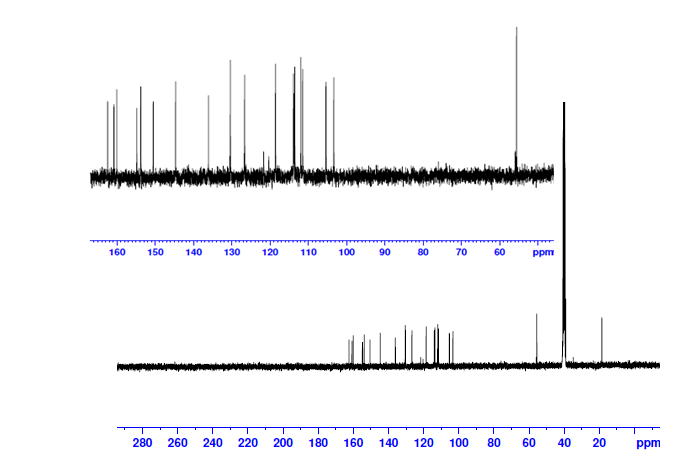
**

**Figure 5:** ^1^H and^13^C-NMR spectrum for compound **(9e)**

**
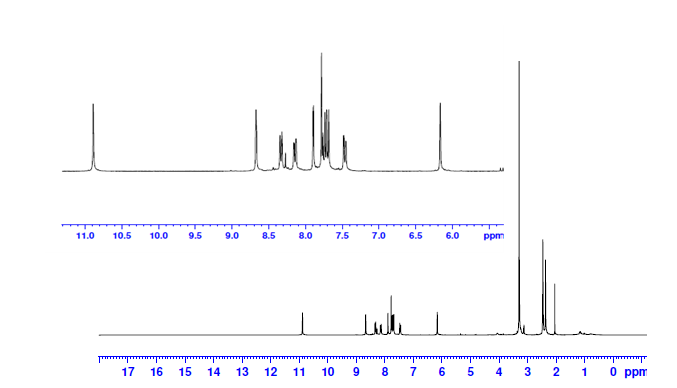
**

**
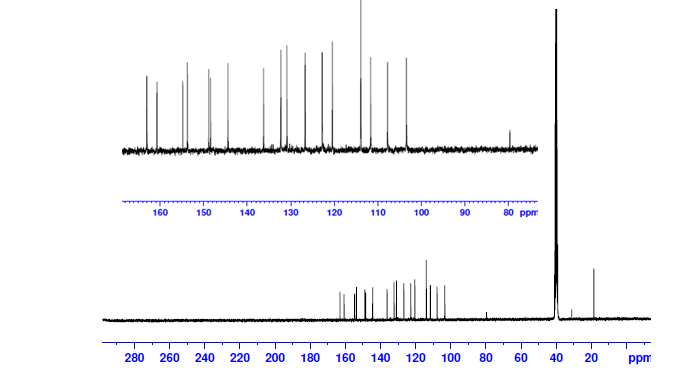
**

**Figure 6:** ^1^H and^13^C-NMR spectrum for compound **(9f)**

**
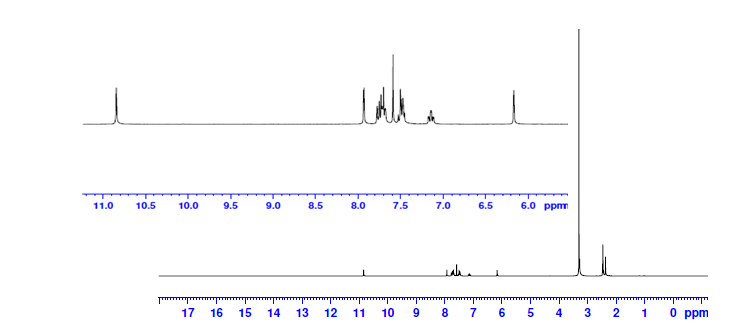
**

**
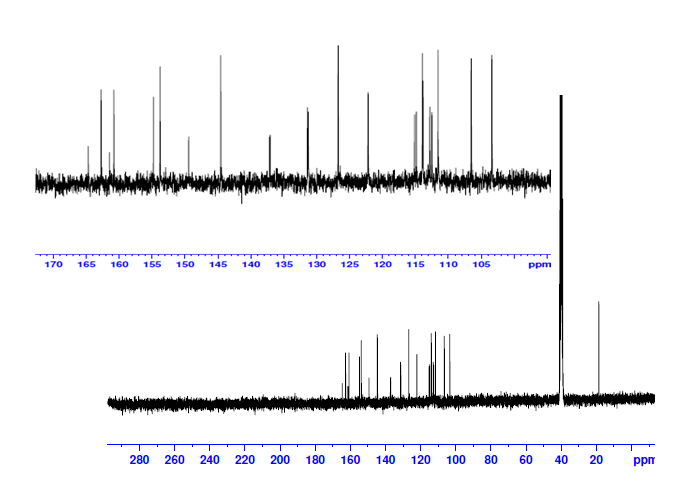
**

**Figure 7:** ^1^H and^13^C-NMR spectrum for compound **(9g)**

**
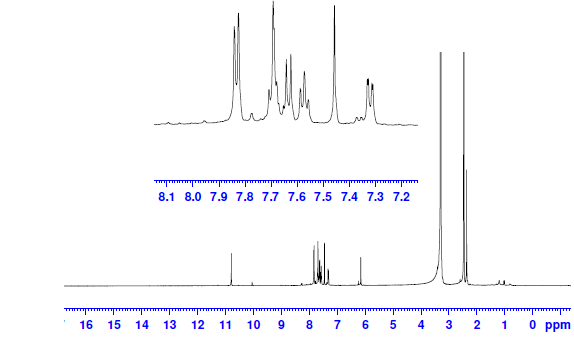
**

**
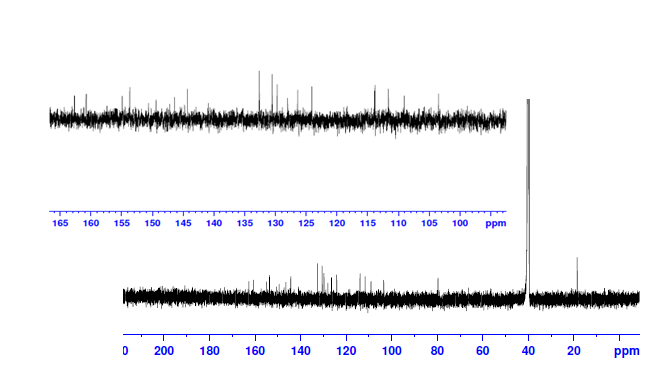
**

**Figure 8:** ^1^H and^13^C-NMR spectrum for compound **(9h)**

**
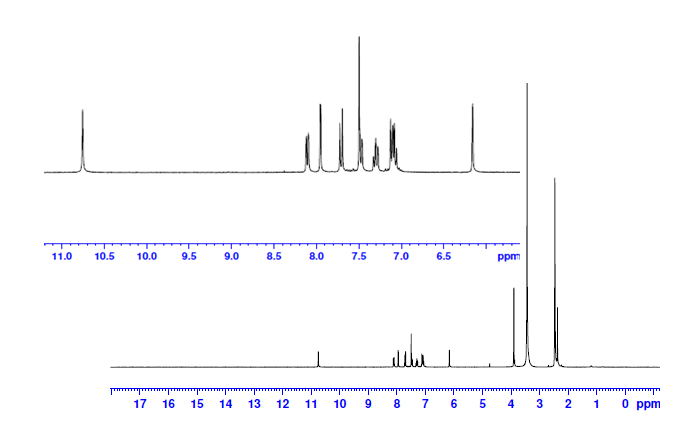
**

**
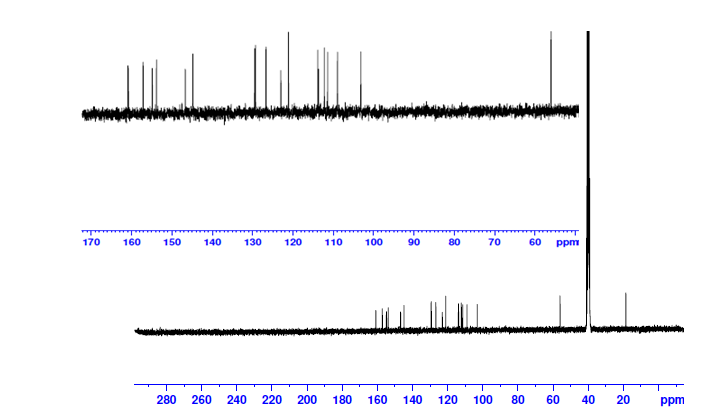
**

**Figure 9:** ^1^H and^13^C-NMR spectrum for compound **(9i)**

**
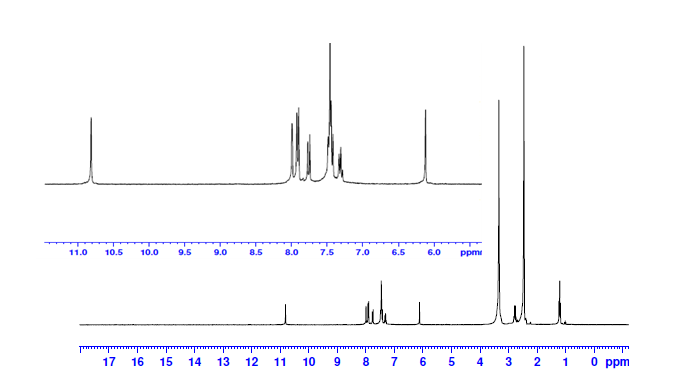
**

**
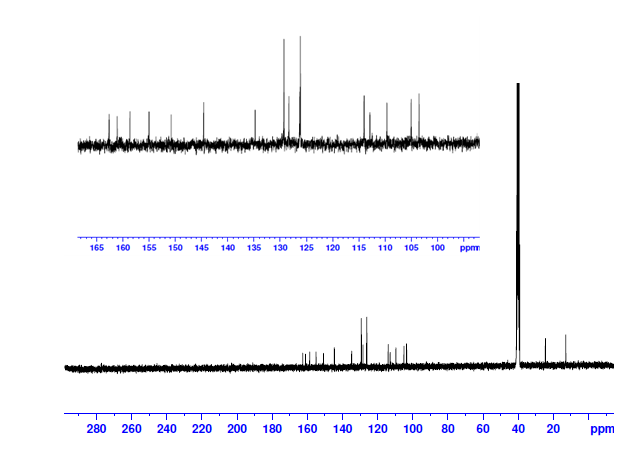
**

**Figure 10:** ^1^H and^13^C-NMR spectrum for compound **(10a)**

**
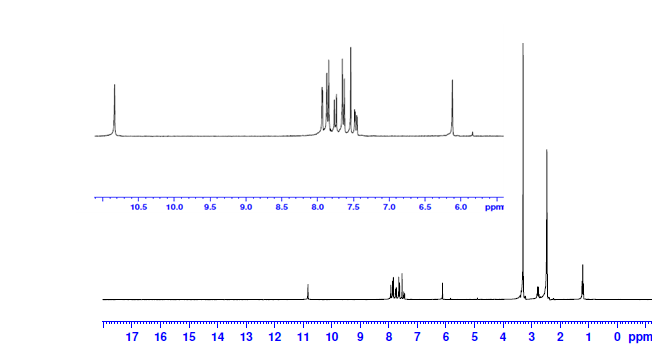
**

**
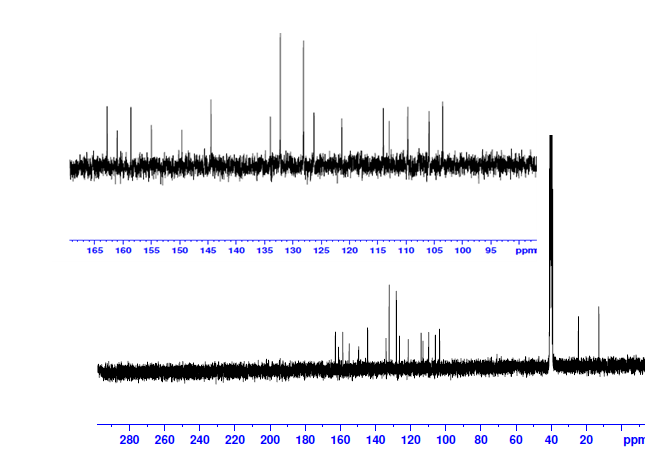
**

**Figure 11:** ^1^H and^13^C-NMR spectrum for compound **(10b)**

**
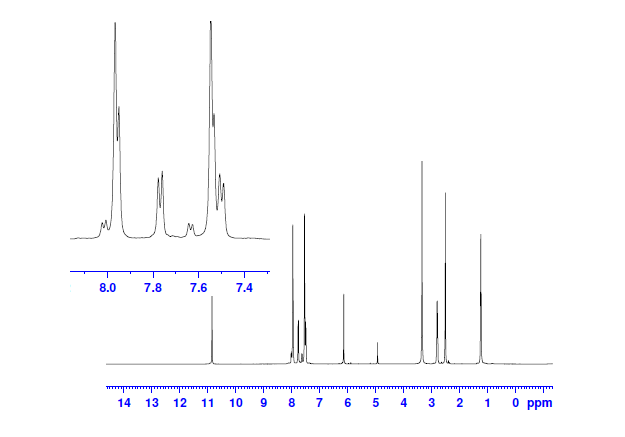
**

**
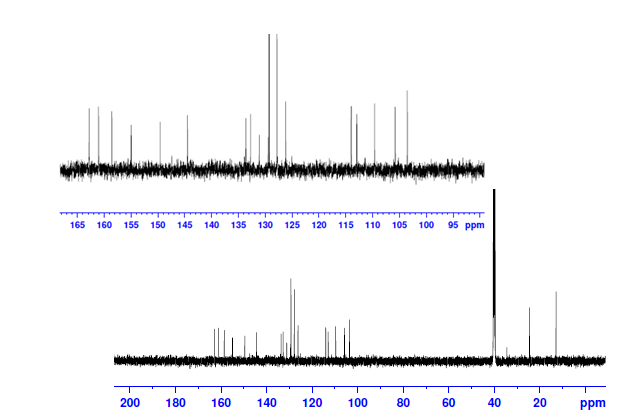
**

**Figure 12:** ^1^H and^13^C-NMR spectrum for compound **(10c)**

**
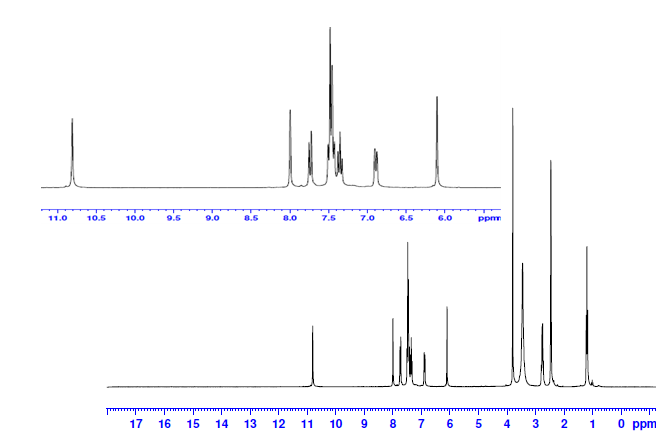
**

**
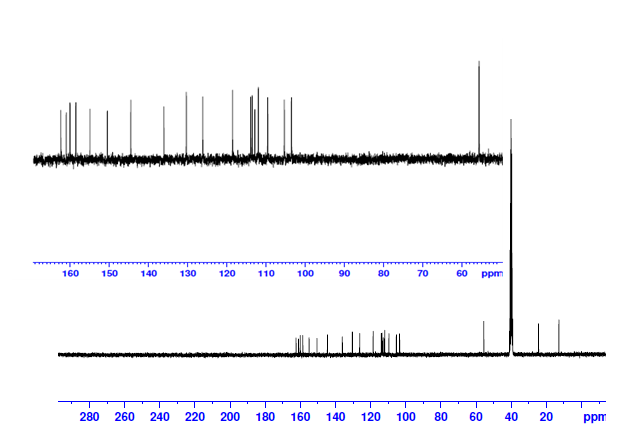
**

**Figure 13:** ^1^H and^13^C-NMR spectrum for compound **(10e)**

**
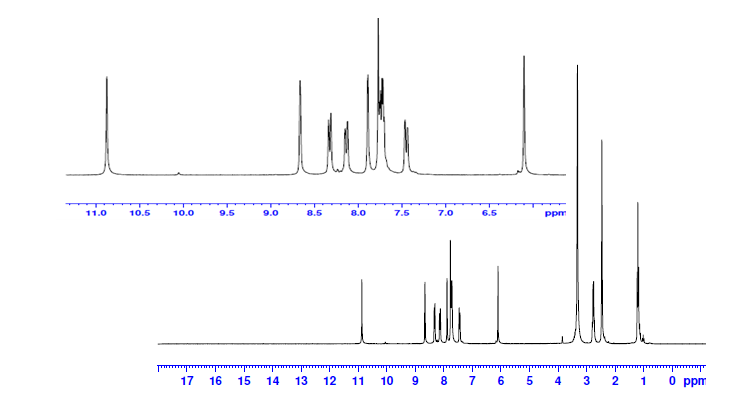
**

**
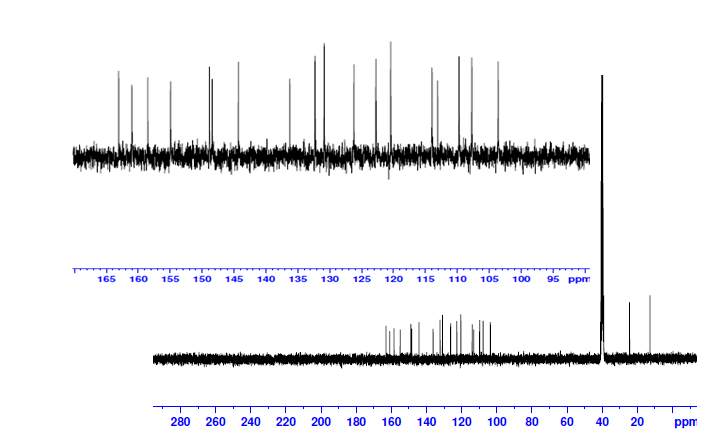
**

**Figure 14:** ^1^H and^13^C-NMR spectrum for compound **(10f)**

**
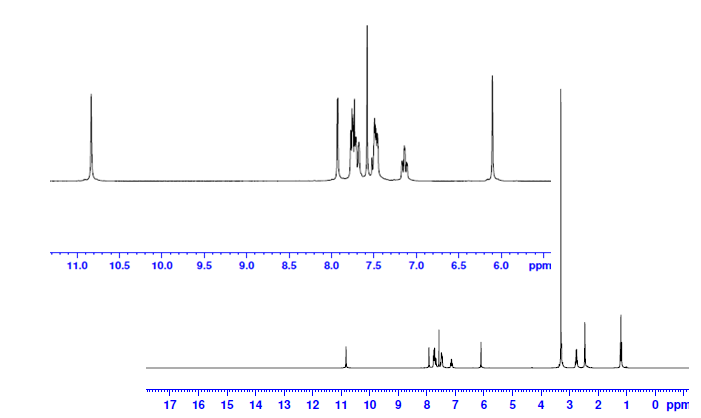
**

**
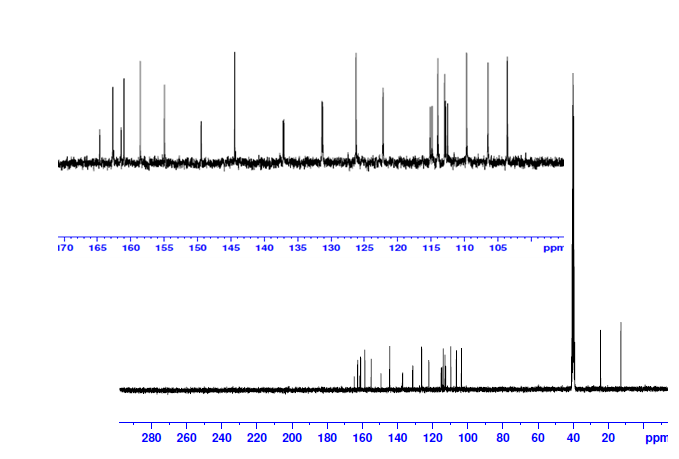
**

**Figure 15:** ^1^H and^13^C-NMR spectrum for compound **(10g)**

**
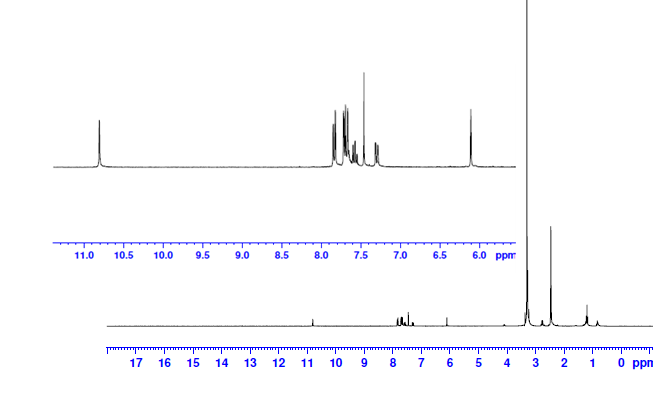
**

**
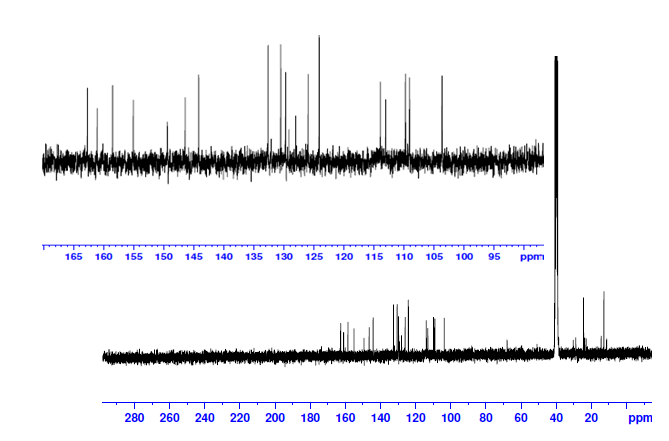
**

**Figure 16:** ^1^H and^13^C-NMR spectrum for compound **(10h)**

**
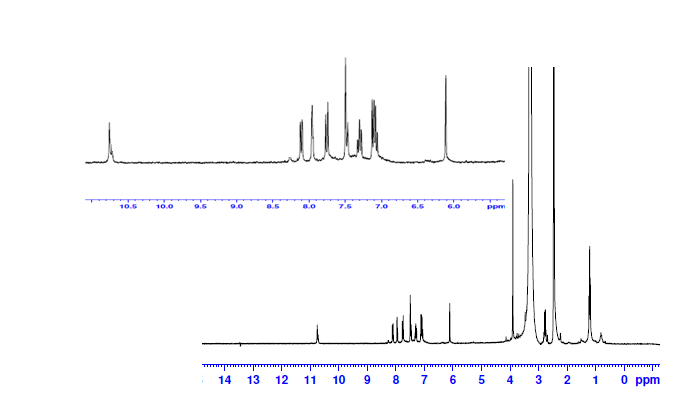
**

**
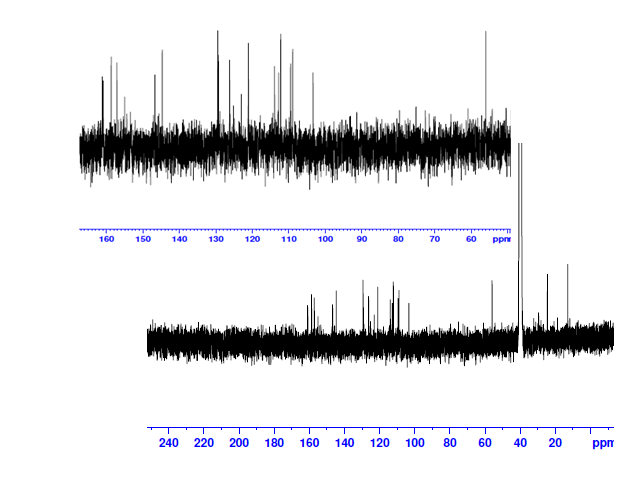
**

**Figure 17:** ^1^H and^13^C-NMR spectrum for compound **(10i)**

**
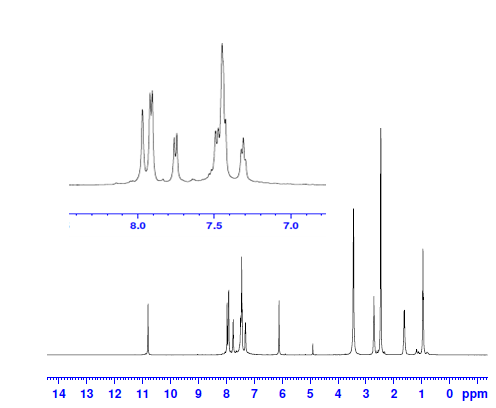
**

**
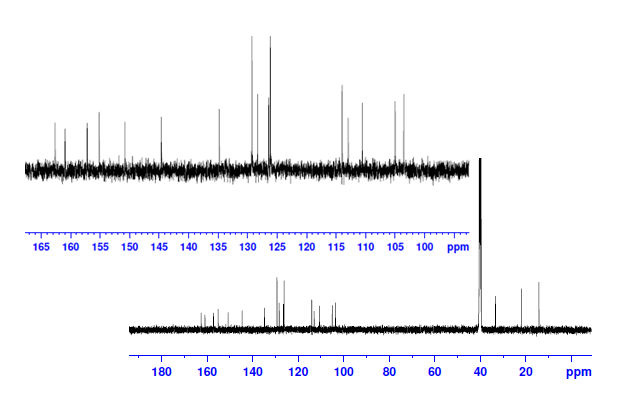
**

**Figure 18:** ^1^H and^13^C-NMR spectrum for compound **(11a)**

**
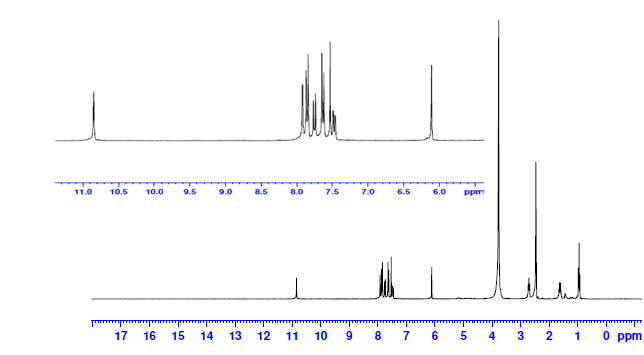
**

**
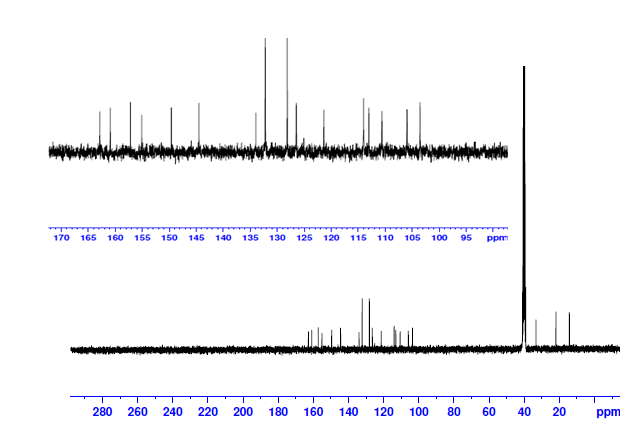
**

**Figure 19:** ^1^H and^13^C-NMR spectrum for compound **(11b)**

**
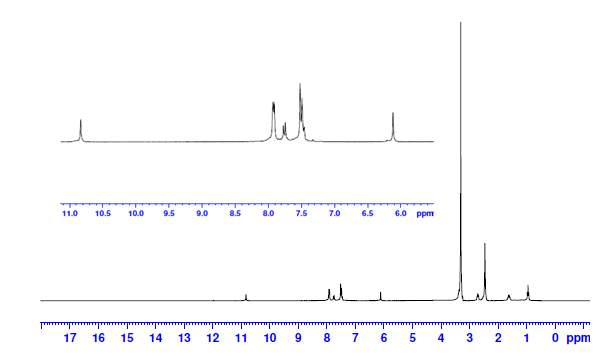
**


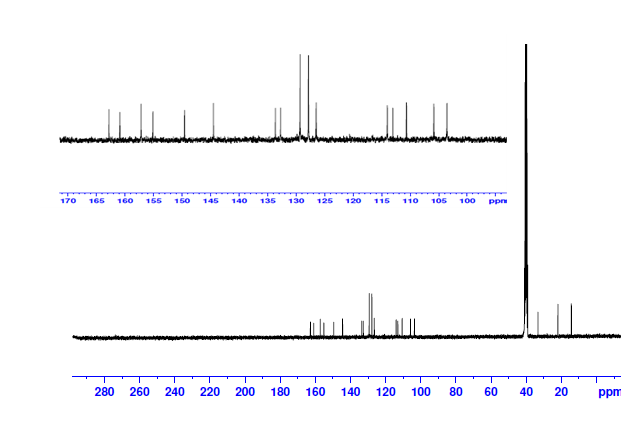


**Figure 20:** ^1^H and^13^C-NMR spectrum for compound **11c.**

**
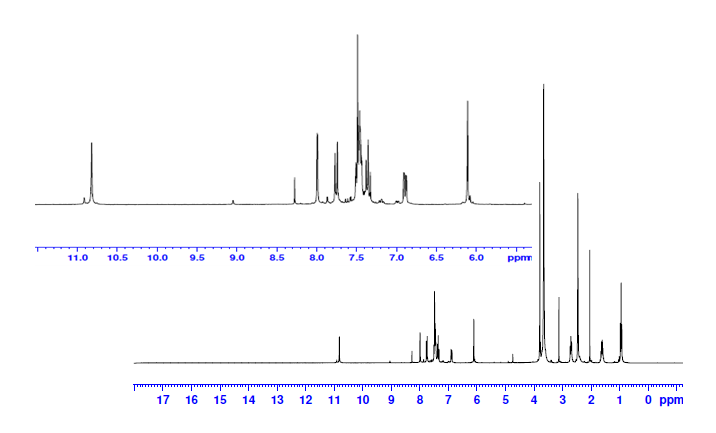
**


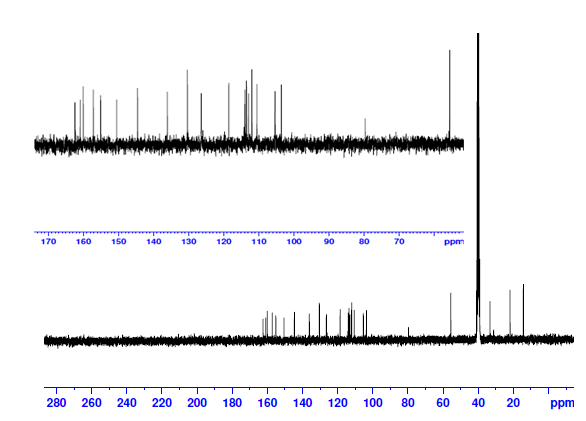


**Figure 21:** ^1^H and^13^C-NMR spectrum for compound **11e.**

**
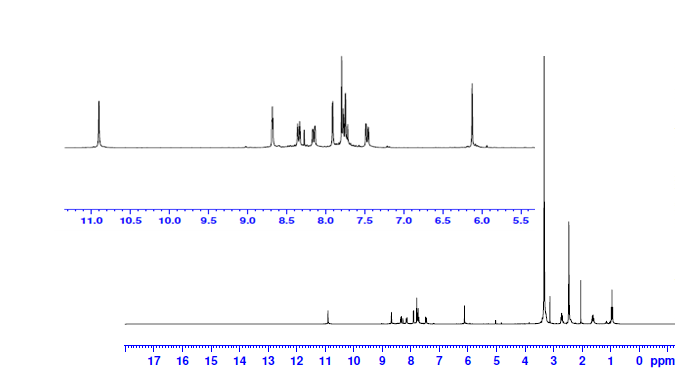
**


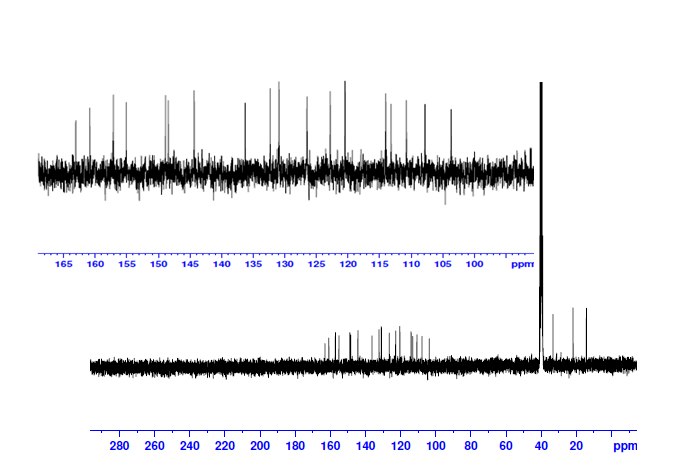


**Figure 22:** ^1^H and^13^C-NMR spectrum for compound **11f.**

**
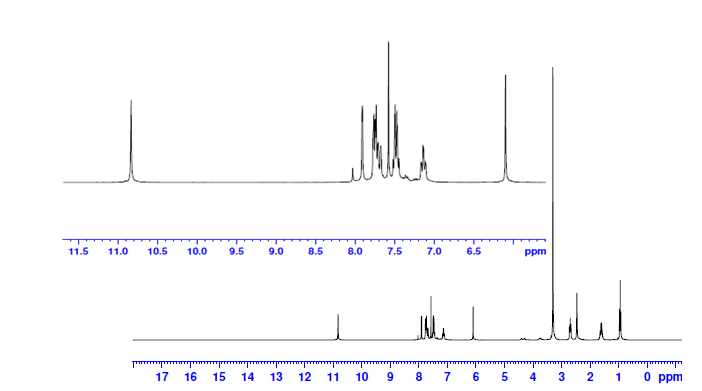
**


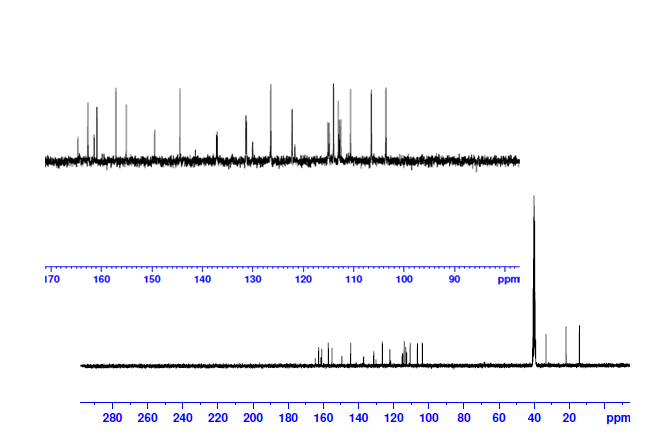


**Figure 23:** ^1^H and^13^C-NMR spectrum for compound **11g.**

**
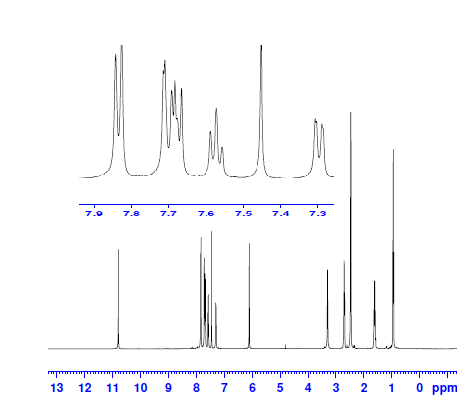
**

**
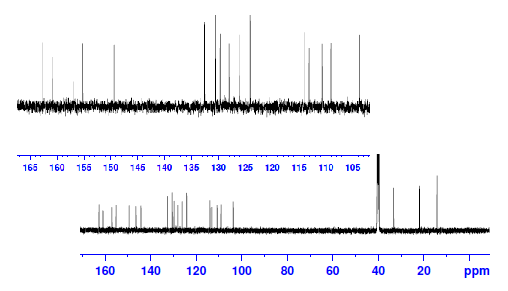
**

**Figure 24:** ^1^H and^13^C-NMR spectrum for compound **11h.**

**Full length Western blot gels**

**Fig. 2A**

**Control**

**Control**

**1**

**9f (µM)**

**2**

**4**

**0.5**

**9b (µM)**

**1**

**2**


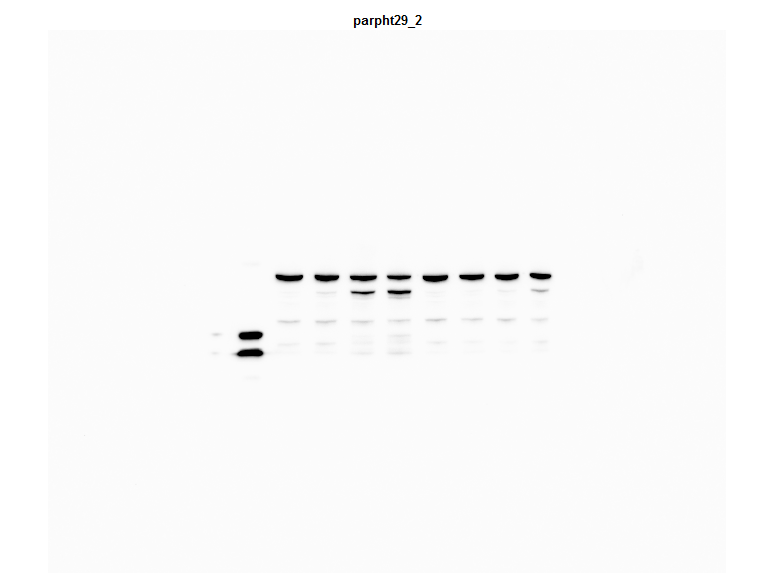


**116KDa**

**89KDa**

**PARP**

**Cleaved PARP**

**50KDa**

**37KDa**

**pAkt-Ser473**

**Control**

**Control**

**1**

**9f (µM)**

**2**

**4**

**0.5**

**9b (µM)**

**1**

**2**


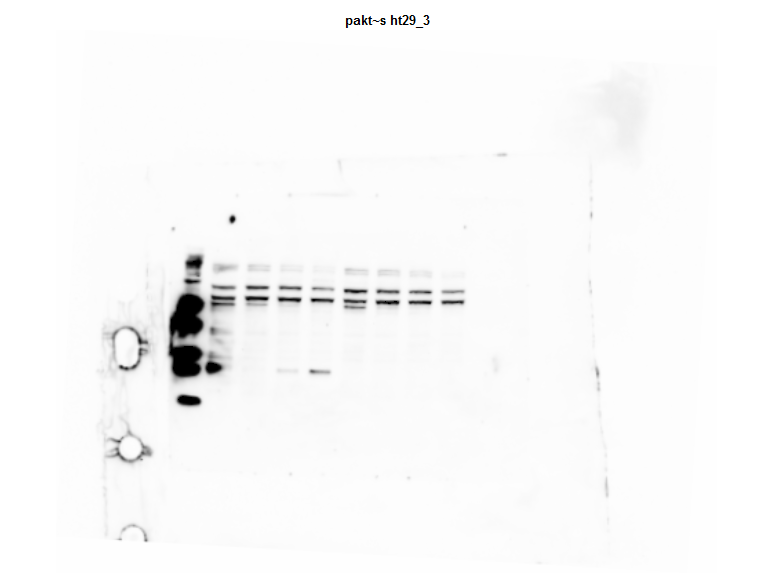


**50KDa**

**37KDa**

**60KDa**

**Control**

**Control**

**1**

**9f (µM)**

**2**

**4**

**0.5**

**9b (µM)**

**1**

**2**


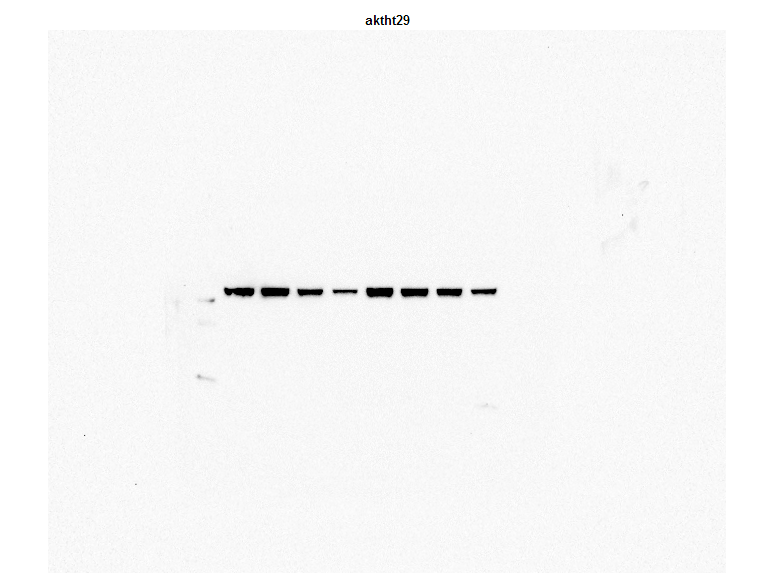


**Akt**

**50KDa**

**60KDa**

**Control**

**Control**

**1**

**9f (µM)**

**2**

**4**

**0.5**

**9b (µM)**

**1**

**2**


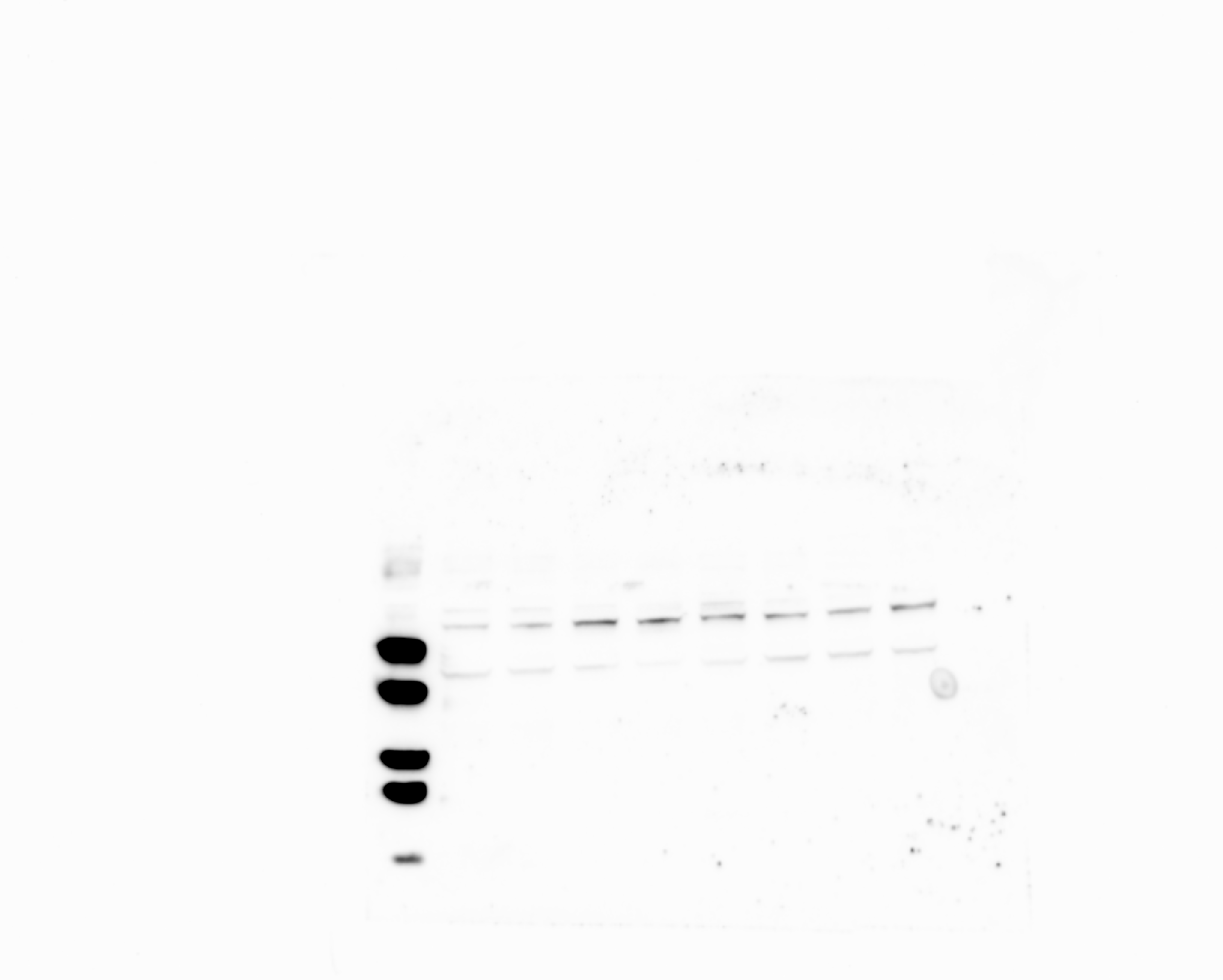


50KDa

37KDa

**62KDa**

**p AMPK**

**Control**

**Control**

**1**

**9f (µM)**

**2**

**4**

**0.5**

**9b (µM)**

**1**

**2**


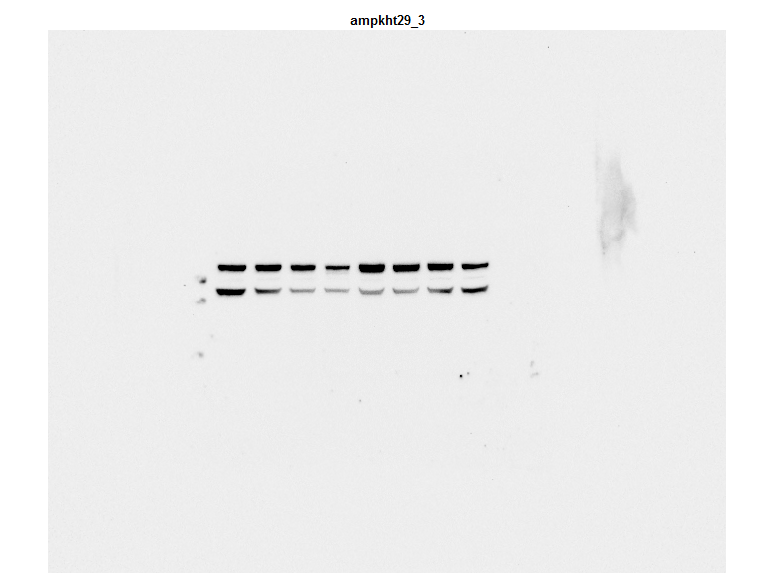


**62KDa**

**AMPK - α**

**50KDa**

**37KDa**

**Control**

**Control**

**1**

**9f (µM)**

**2**

**4**

**0.5**

**9b (µM)**

**1**

**2**


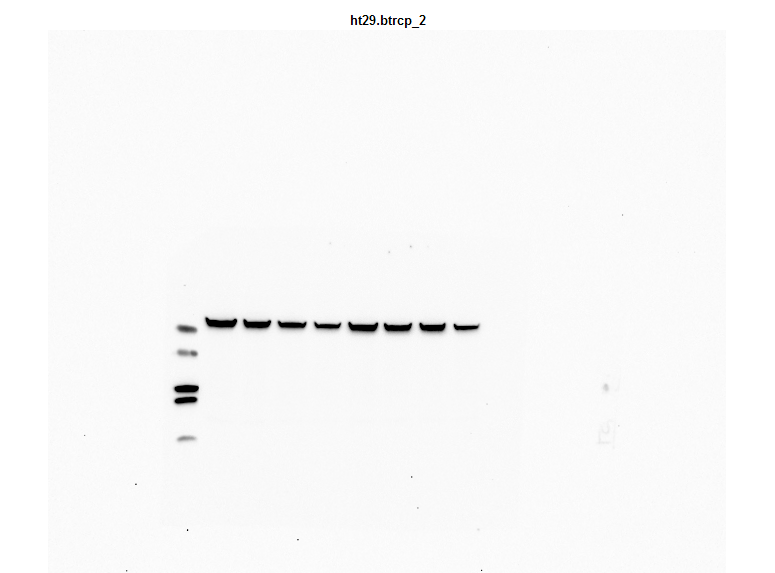


**β-TrCP**

**50KDa**

**37KDa**

**62KDa**

**Control**

**Control**

**1**

**9f (µM)**

**2**

**4**

**0.5**

**9b (µM)**

**1**

**2**

**LC3A/B-I**

**LC3A/B-II**


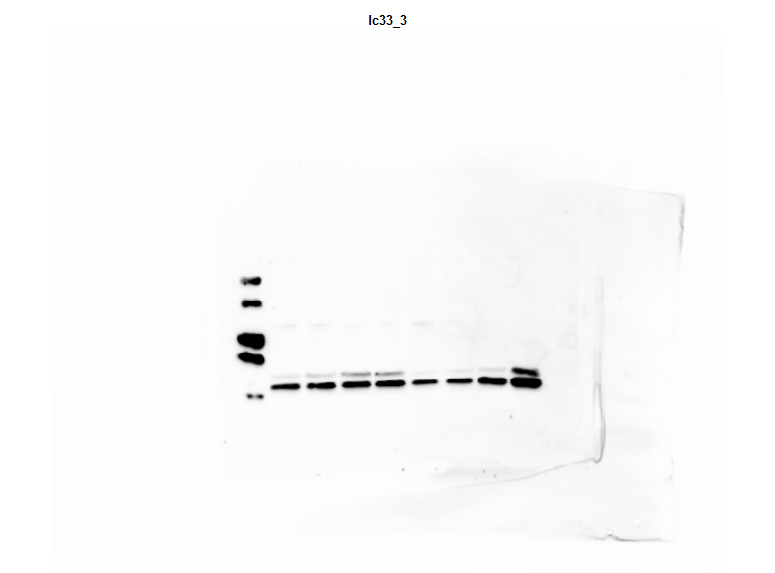


**25KDa**

**20KDa**

**10KDa**

**16KDa**

**14KDa**

**Control**

**Control**

**1**

**9f (µM)**

**2**

**4**

**0.5**

**9b (µM)**

**1**

**2**

**Caspase 3**


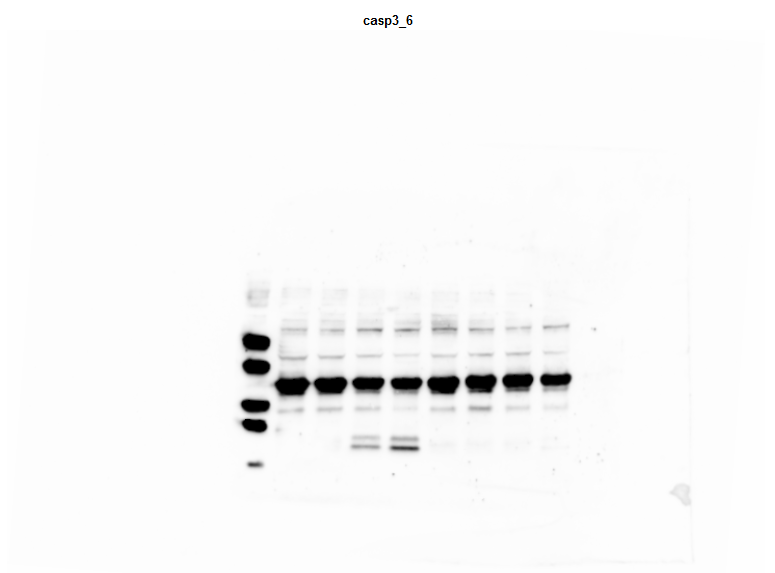


**50KDa**

**37KDa**

**25KDa**

**20KDa**

**10KDa**

**10KDa**

**35KDa**

**19KDa**

**17KDa**

**Control**

**Control**

**1**

**9f (µM)**

**2**

**4**

**0.5**

**9b (µM)**

**1**

**2**

**Cleaved**

**Caspase 3**


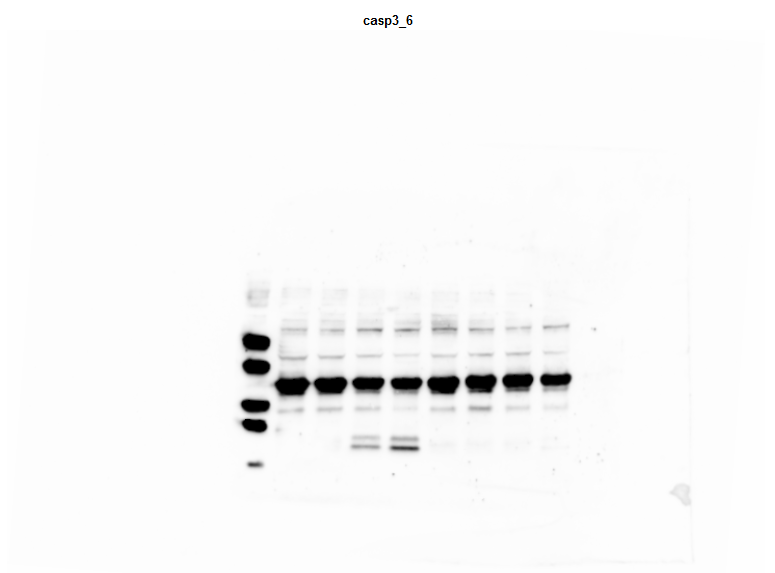


**50KDa**

**37KDa**

**25KDa**

**20KDa**

**10KDa**

**10KDa**

**35KDa**

**19KDa**

**17KDa**

**Control**

**Control**

**1**

**9f (µM)**

**2**

**4**

**0.5**

**9b (µM)**

**1**

**2**

**Cyclin D1**


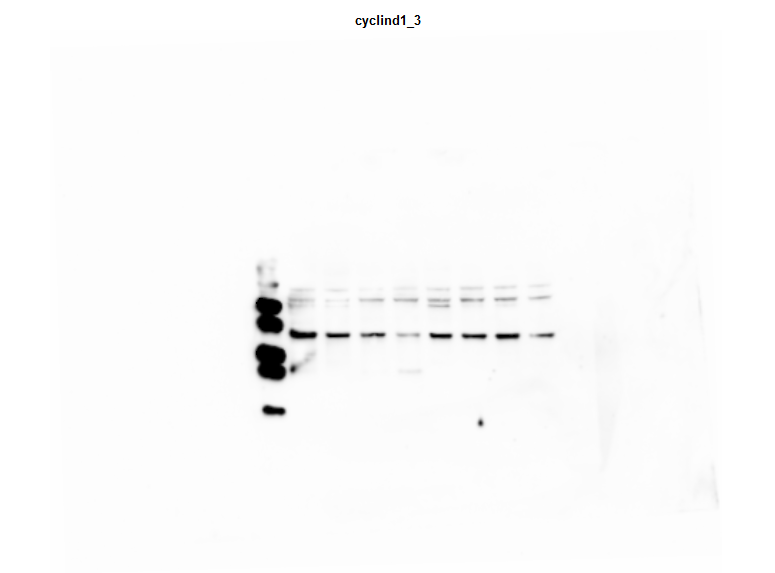


**50KDa**

**37KDa**

**25KDa**

**20KDa**

**10KDa**

**10KDa**

**36KDa**

Control

Control

**9b (µM)**

**9f (µM)**

**0.5**

**1**

**2**

**1**

**2**

**4**

**Bcl-2**


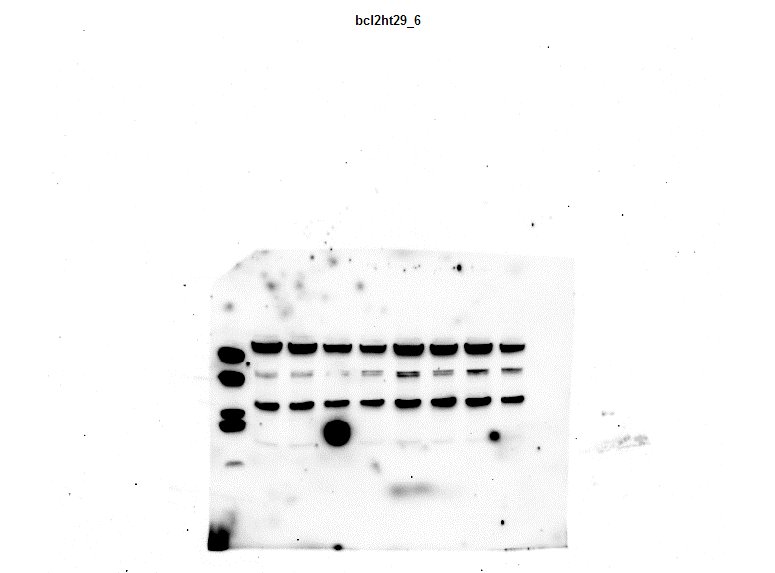


**50KDa**

**37KDa**

**25KDa**

**20KDa**

**10KDa**

**10KDa**

**26KDa**

**β-Actin**


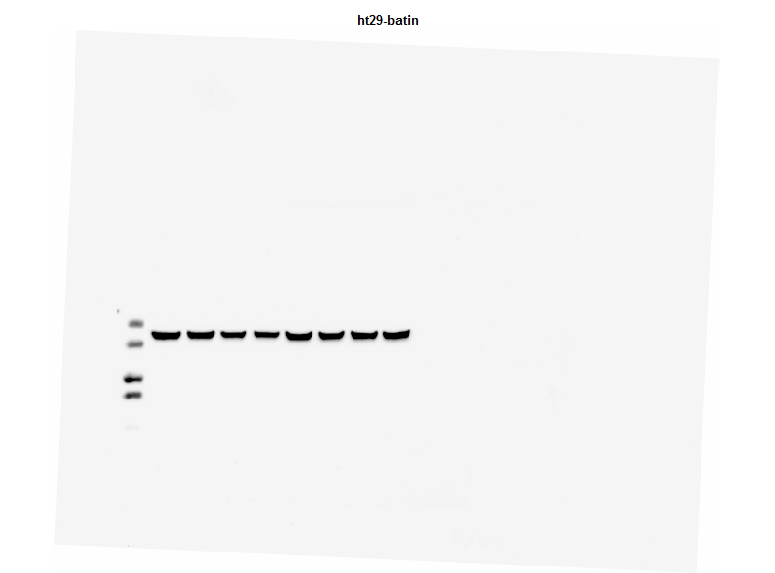


**2**

**1**

**9b (µM)**

**0.5**

**9f (µM)**

Control

Control

**4**

**2**

**1**

**50KDa**

**37KDa**

**25KDa**

**20KDa**

**10KDa**

**10KDa**

**45KDa**

**Fig. 2B**


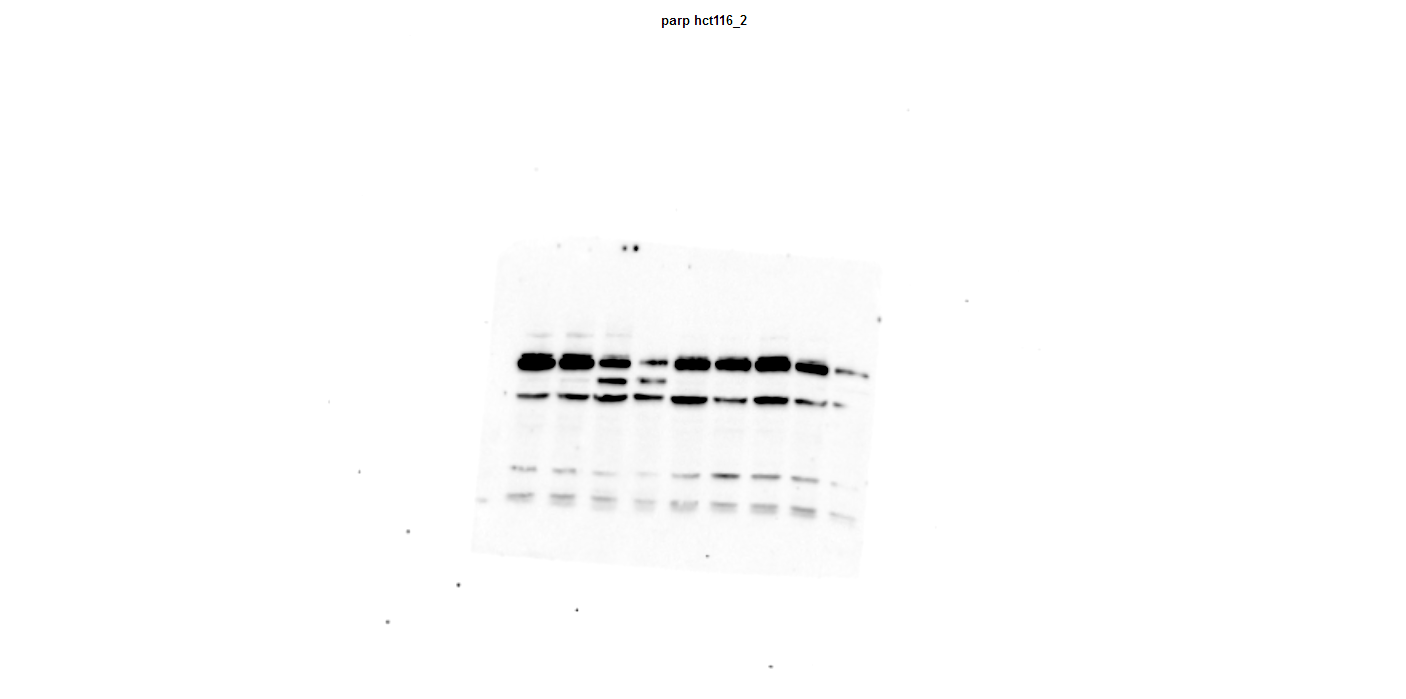


Control

Control

**4**

**0.5**

**9b (µM)**

**9f (µM)**

**PARP**

**Cleaved PARP**

**25KDa**

**10KDa**

**10KDa**

**2**

**1**

**4**

**2**

**1**

**116KDa**

**89KDa**


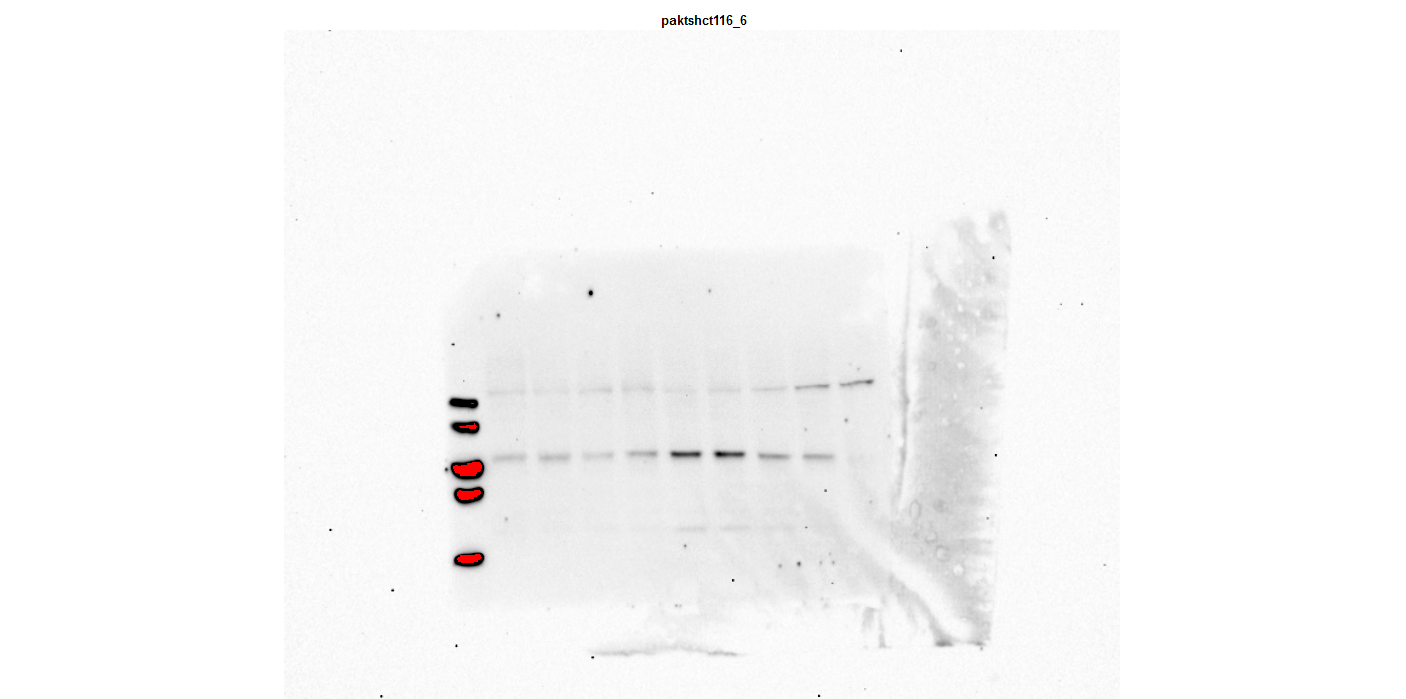


Control

Control

**9f (µM)**

**9b (µM)**

**pAkt-Ser473**

**2**

**1**

**4**

**2**

**1**

**0.5**

**60KDa**

**50KDa**

**37KDa**

**25KDa**

**20KDa**

**10KDa**

**Akt**

**Control**

**Control**

**1**

**9f (µM)**

**2**

**4**

**0.5**

**9b (µM)**

**1**

**2**


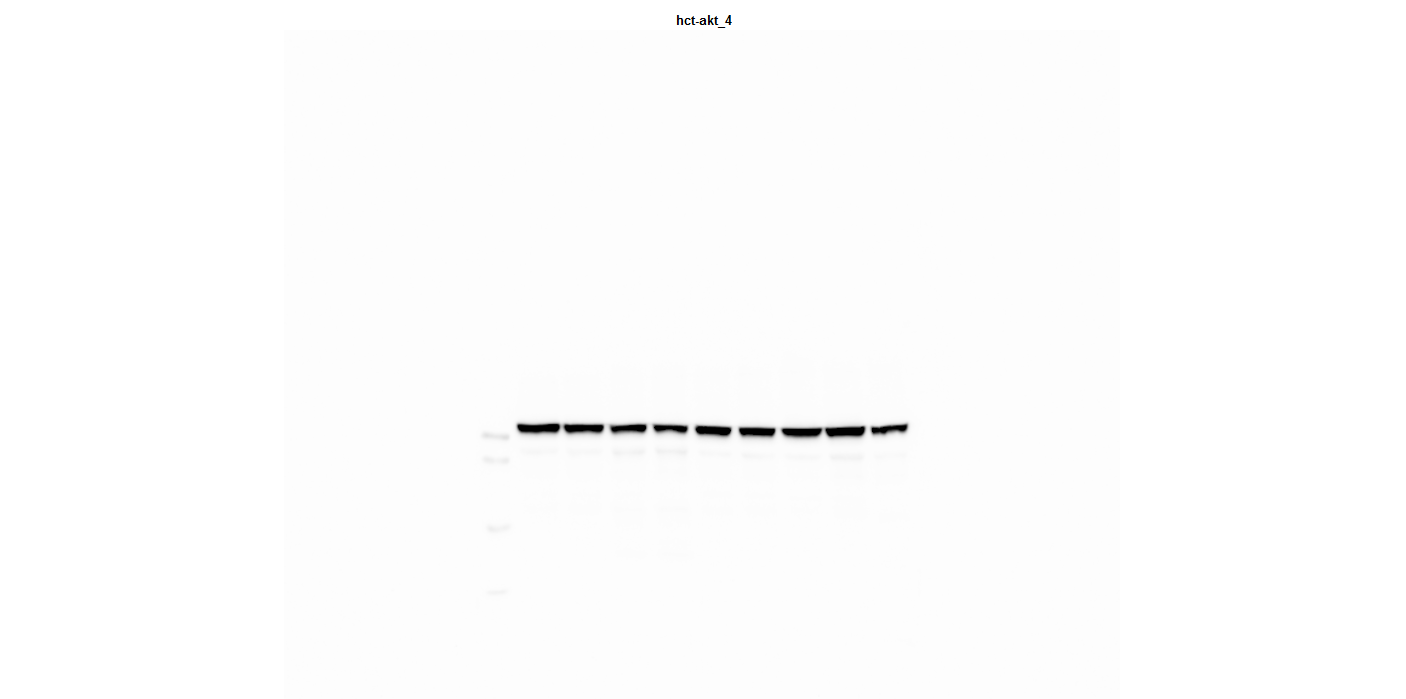


**60KDa**

**50KDa**

**37KDa**

**20KDa**

**10KDa**


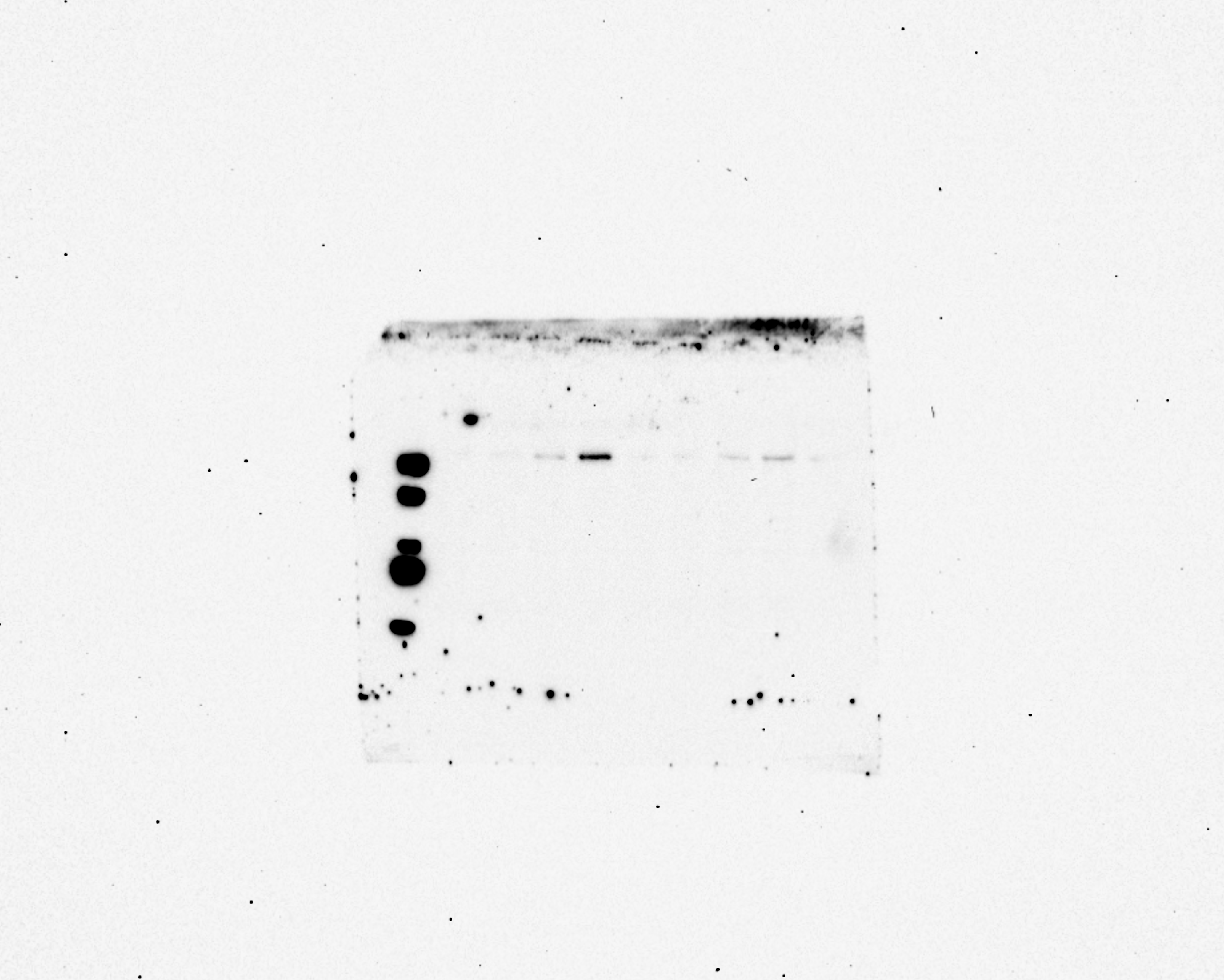


**62KDa**

**50KDa**

**37KDa**

**25KDa**

**20KDa**

**10KDa**

**10KDa**

**Control**

**Control**

**1**

**9f (µM)**

**2**

**4**

**0.5**

**9b (µM)**

**1**

**2**

**p AMPK**

**Control**

**Control**

**1**

**9f (µM)**

**2**

**4**

**0.5**

**9b (µM)**

**1**

**2**

**AMPK - α**


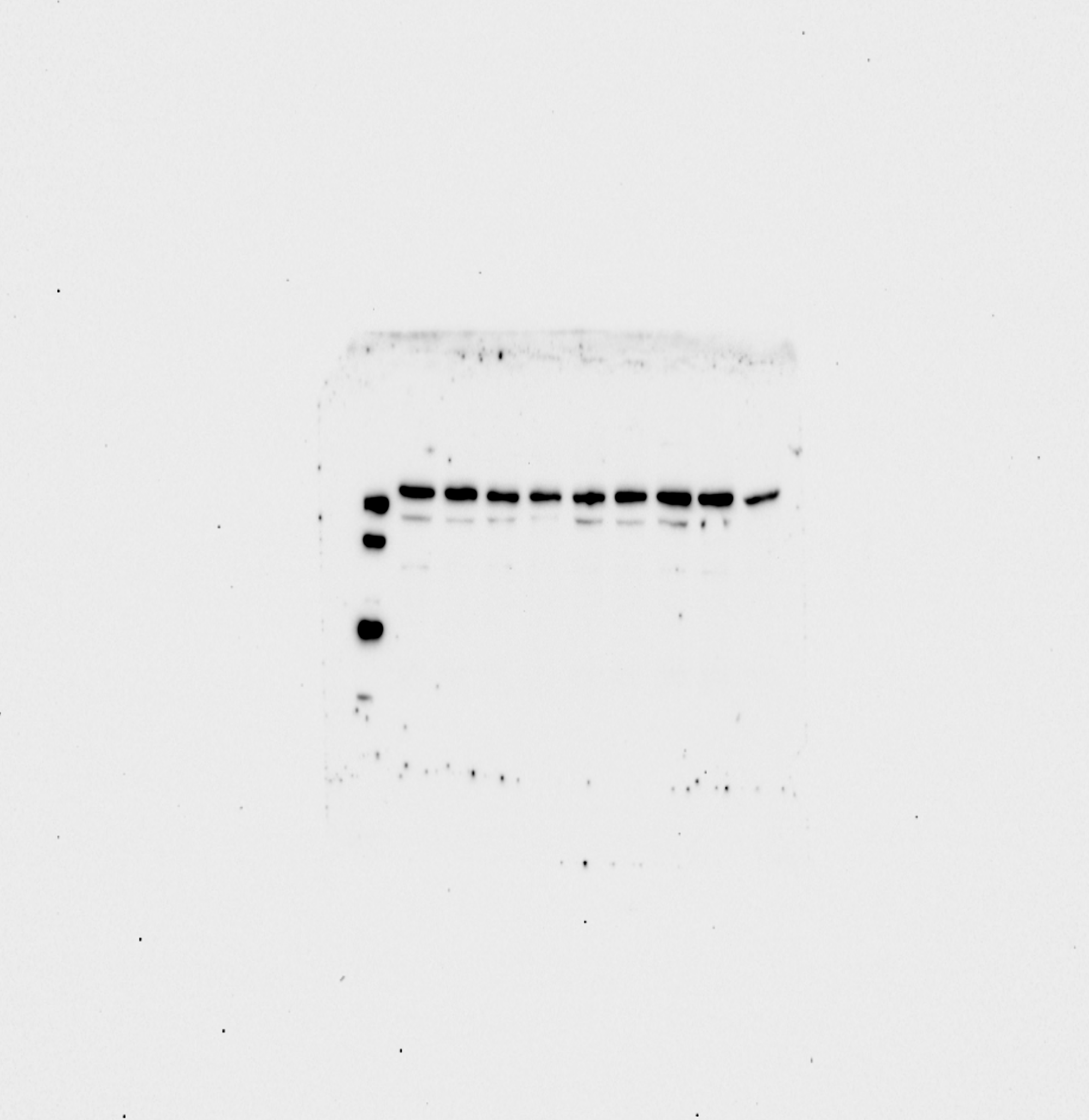


**62KDa**

**50KDa**

**37KDa**

**25KDa**

**20KDa**

**10KDa**

**10KDa**

**Control**

**Control**

**1**

**9f (µM)**

**2**

**4**

**0.5**

**9b (µM)**

**1**

**2**

**β-TrCP**


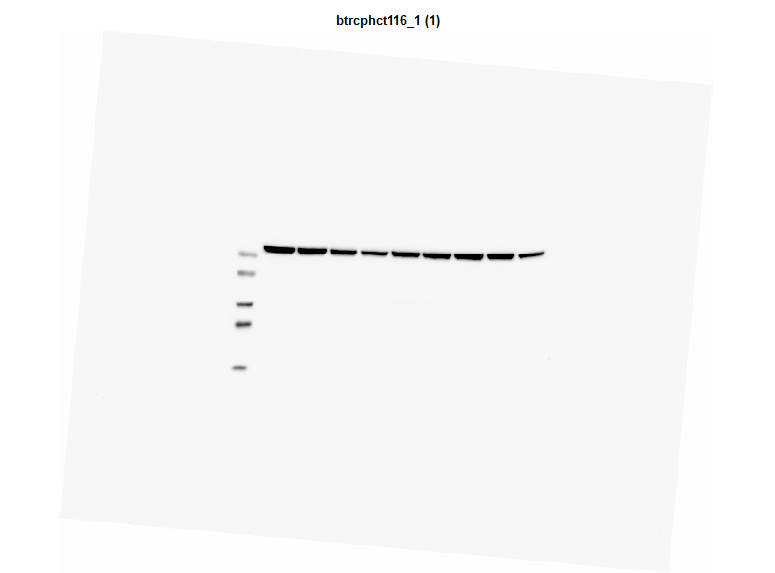


**50KDa**

**37KDa**

**25KDa**

**20KDa**

**10KDa**

**10KDa**

**10KDa**

**62KDa**

**LC3A/B-I**

**LC3A/B-II**

**Control**

**Control**

**1**

**9f (µM)**

**2**

**4**

**0.5**

**9b (µM)**

**1**

**2**


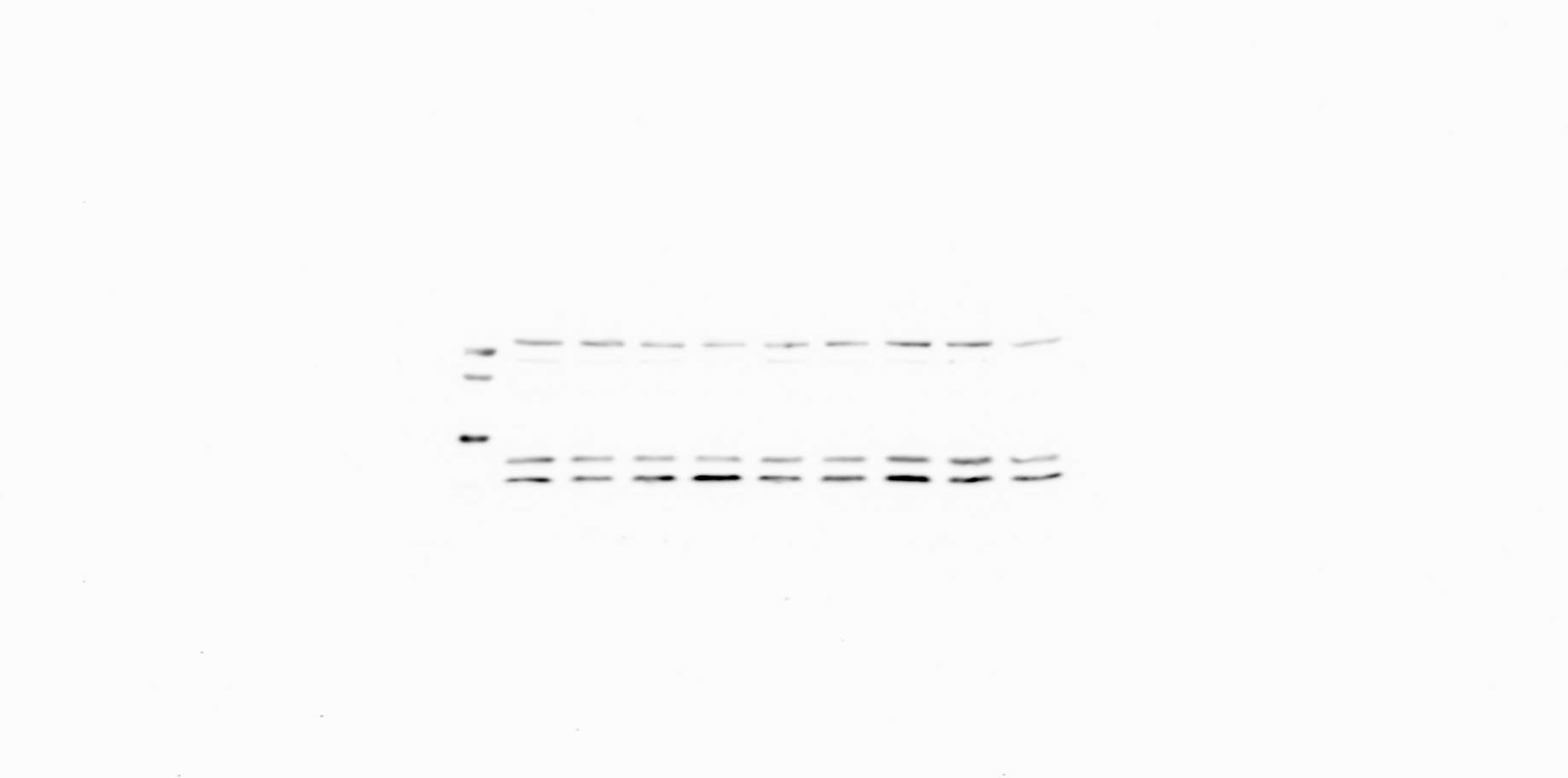


**50KDa**

**37KDa**

**20KDa**

**10KDa**

**16KDa**

**14KDa**

**Control**

**Control**

**1**

**9f (µM)**

**2**

**4**

**0.5**

**9b (µM)**

**1**

**2**

**Caspase 3**


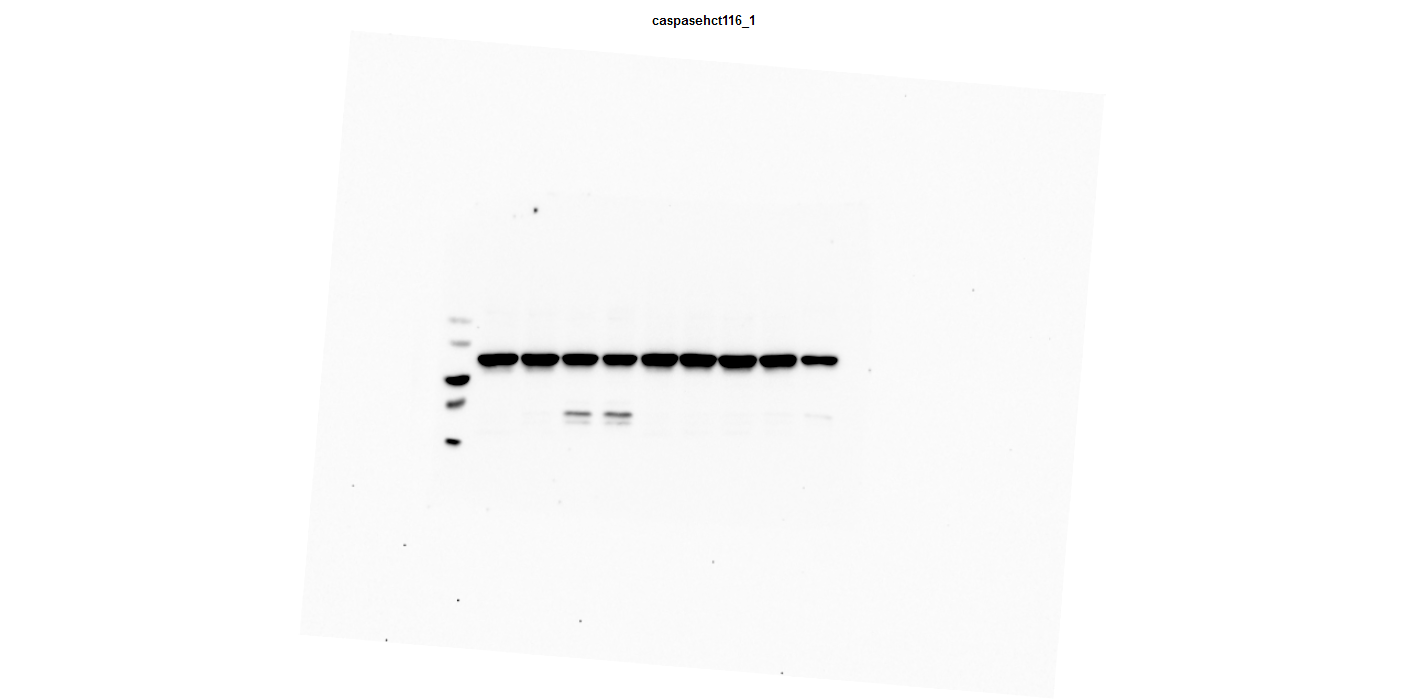


**50KDa**

**37KDa**

**25KDa**

**20KDa**

**10KDa**

**10KDa**

**35KDa**

**19KDa**

**17KDa**

**Control**

**Control**

**1**

**9f (µM)**

**2**

**4**

**0.5**

**9b (µM)**

**1**

**2**

**Cleaved Caspase 3**


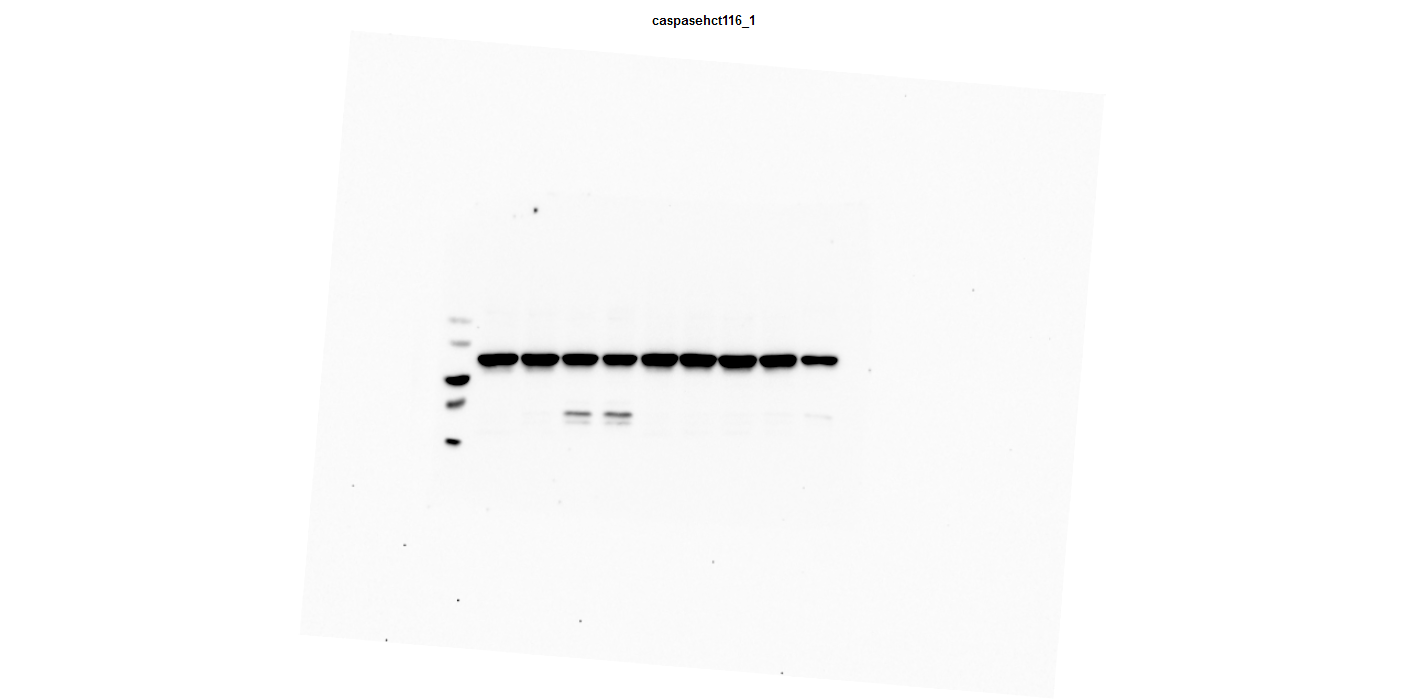


**50KDa**

**37KDa**

**25KDa**

**20KDa**

**10KDa**

**10KDa**

**35KDa**

**19KDa**

**17KDa**

**Control**

**Control**

**1**

**9f (µM)**

**2**

**4**

**0.5**

**9b (µM)**

**1**

**2**

**Cyclin D1**


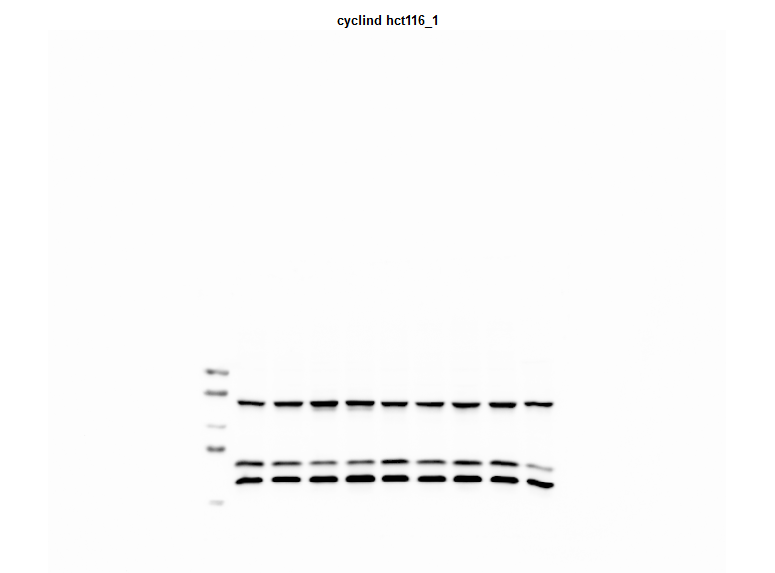


**50KDa**

**37KDa**

**25KDa**

**20KDa**

**10KDa**

**36KDa**

**Control**

**Control**

**1**

**9f (µM)**

**2**

**4**

**0.5**

**9b (µM)**

**1**

**2**

**Bcl-2**


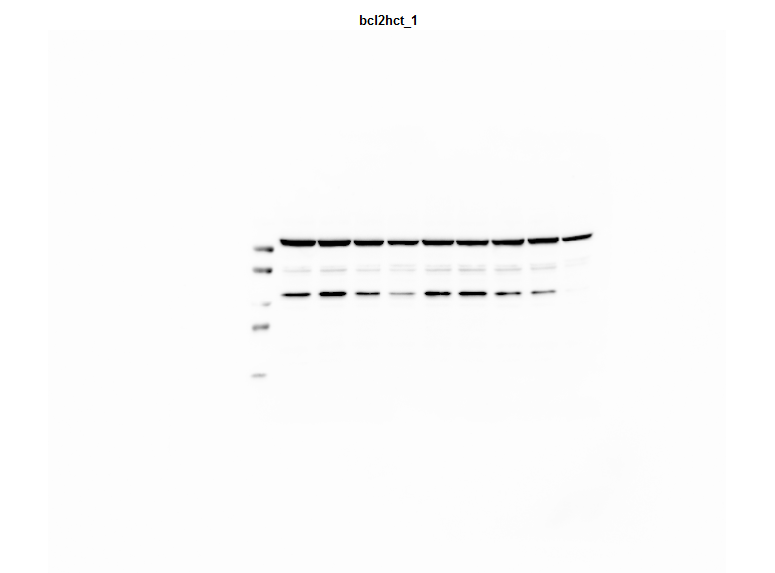


**37KDa**

**25KDa**

**20KDa**

**26KDa**

**Control**

**Control**

**1**

**9f (µM)**

**2**

**4**

**0.5**

**9b (µM)**

**1**

**2**

**Control**

**Control**

**1**

**9f (µM)**

**2**

**4**

**0.5**

**9b (µM)**

**1**

**2**

**β-Actin**


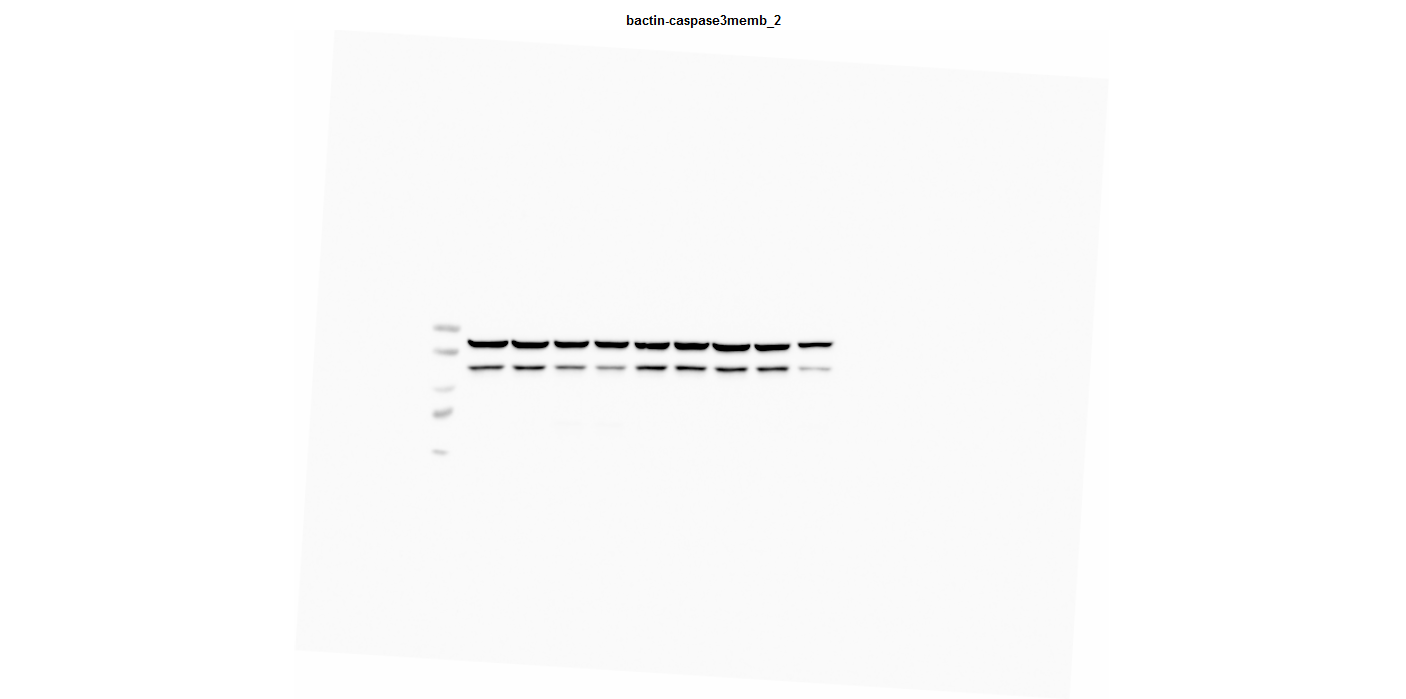


**50KDa**

**37KDa**

**25KDa**

**20KDa**

**10KDa**

**45KDa**

**p-AMPK – α**

**high-exposure**


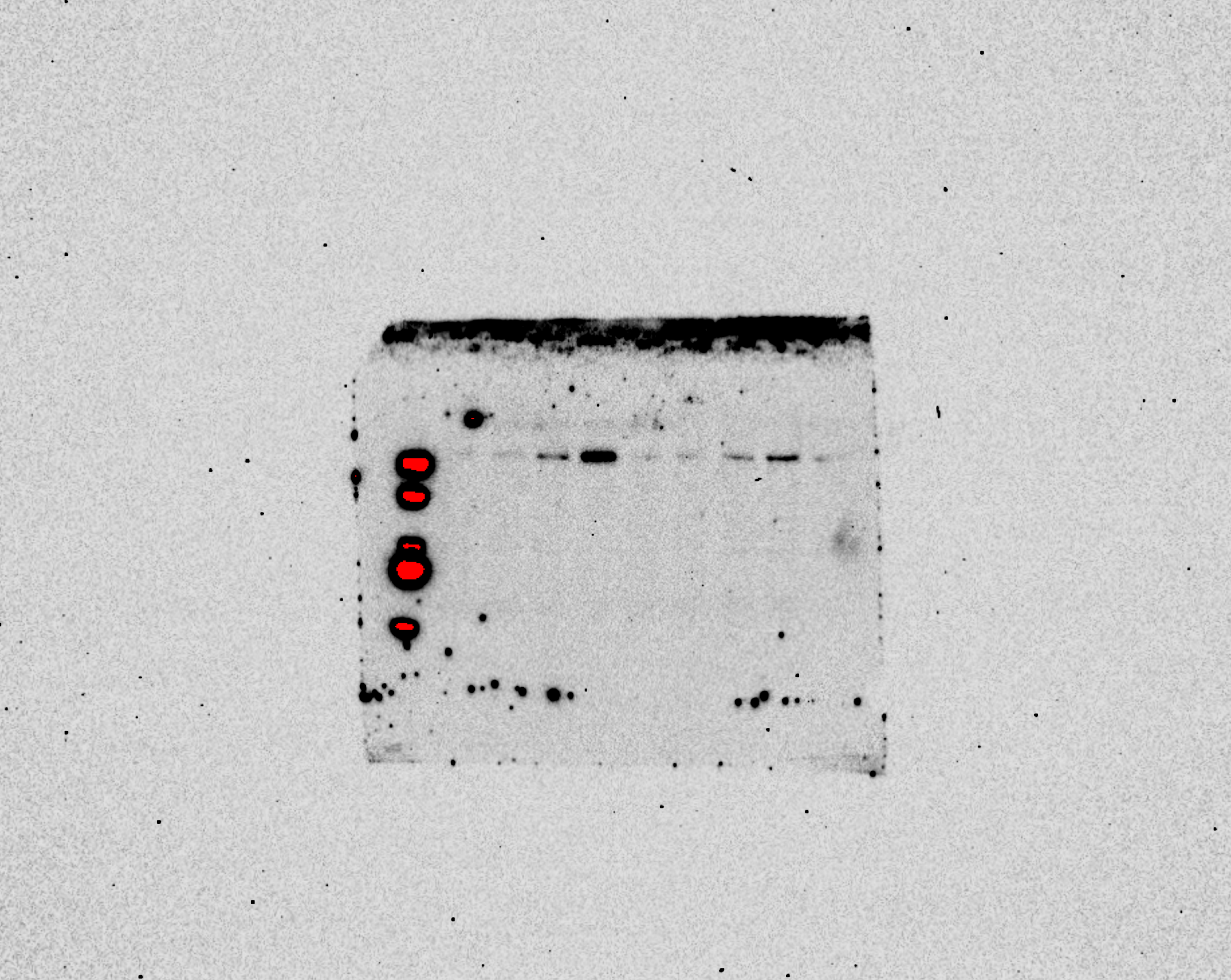


**60KDa**

**Control**

**Control**

**1**

**9f (µM)**

**2**

**4**

**0.5**

**9b (µM)**

**1**

**2**

**Fig. 2c**


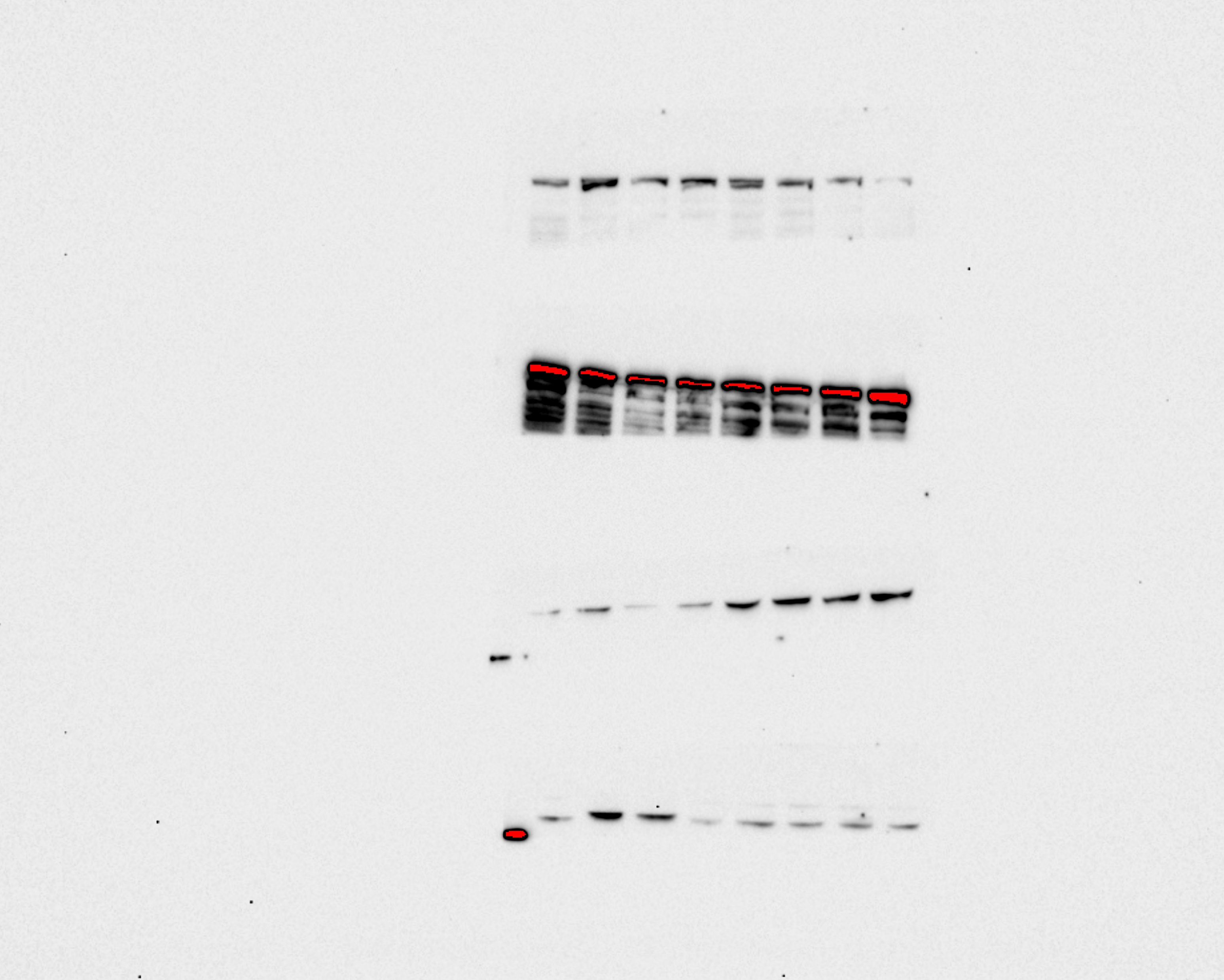


**289KDa**

**p-mTOR**


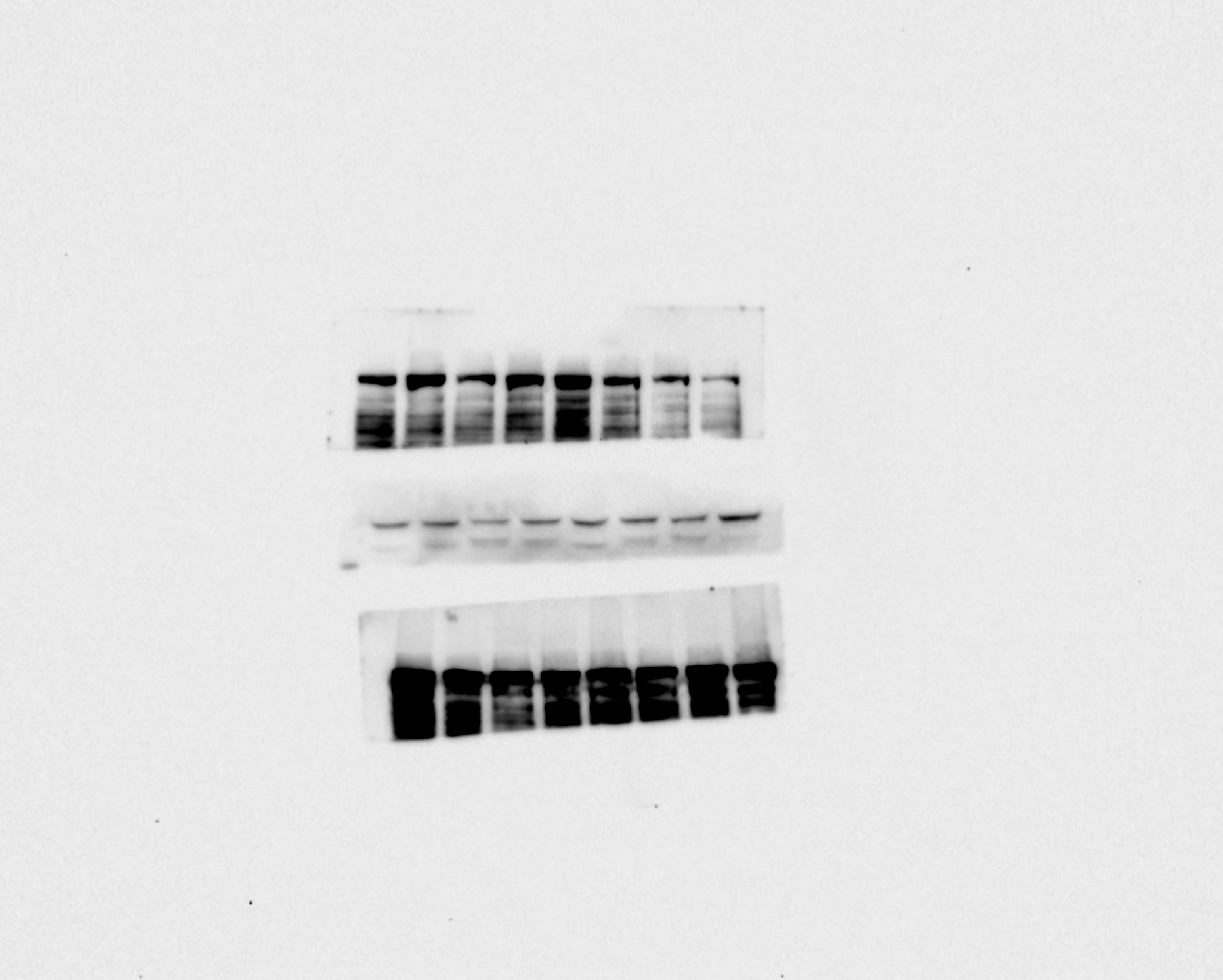


**289KDa**

**mTOR**

**Control**

**Control**

**1**

**9f (µM)**

**2**

**4**

**0.5**

**9b (µM)**

**1**

**2**


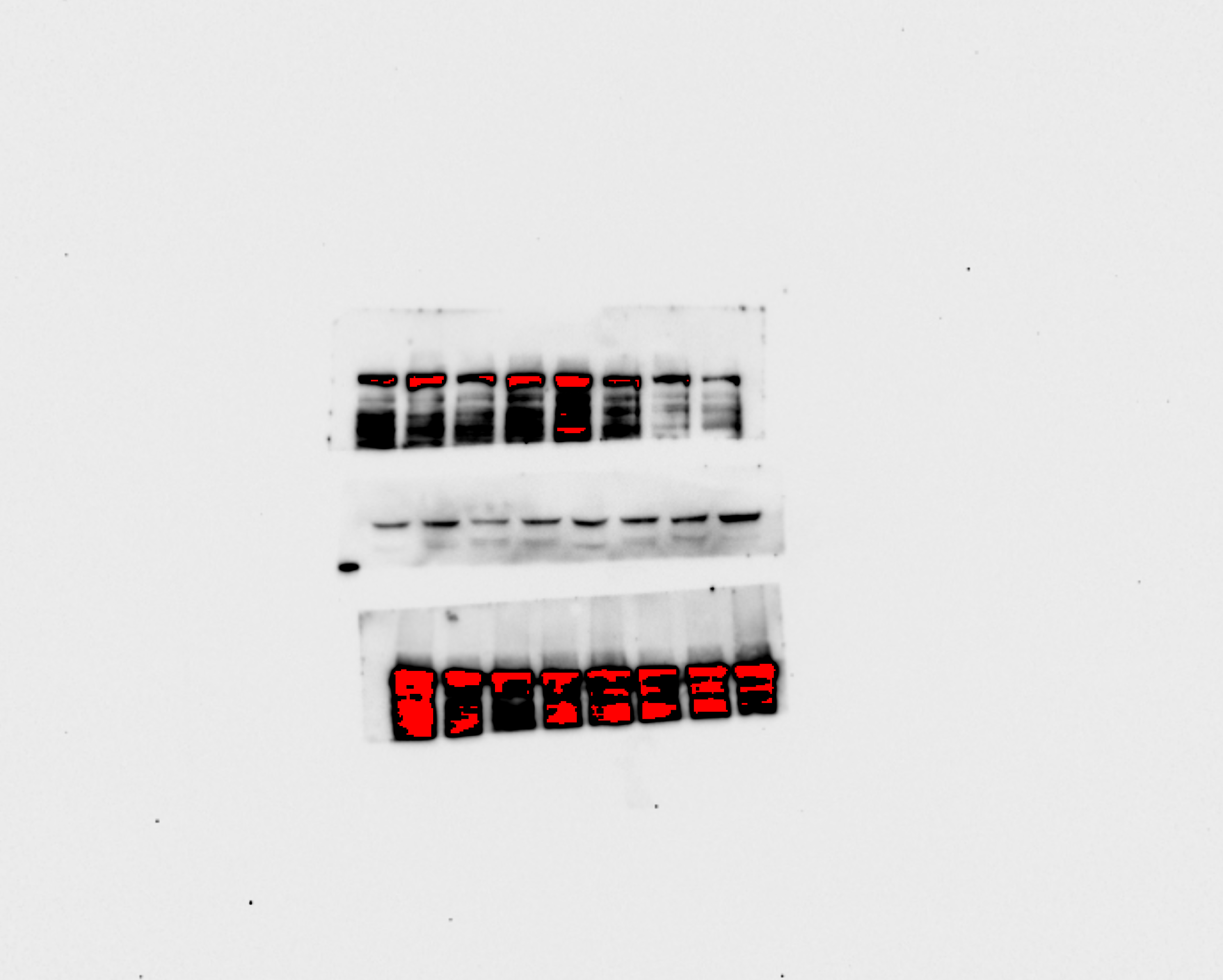


**70KDa**

**β-Actin**

**p-p70S6K**


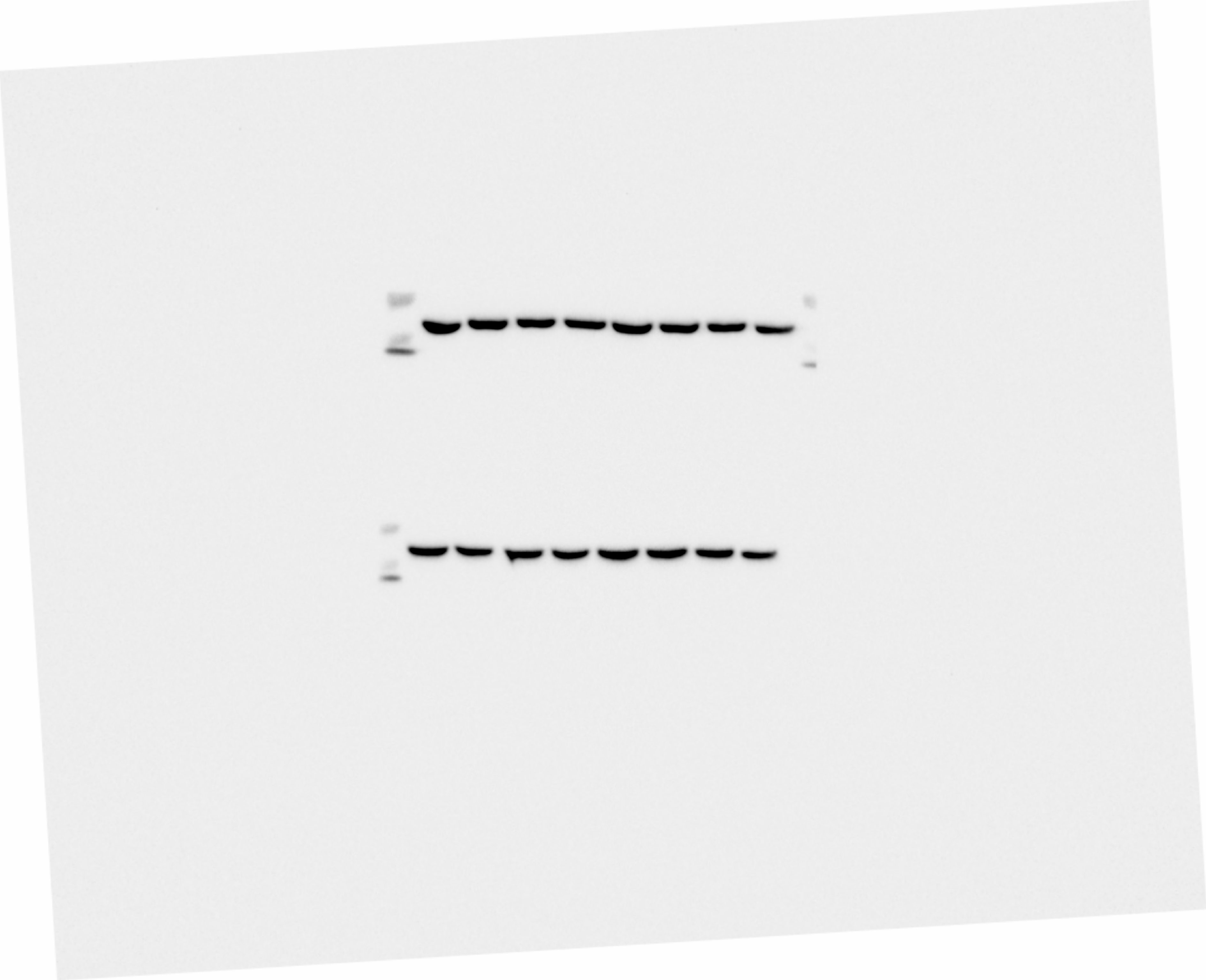


**45KDa**

**50KDa**

**37KDa**

**25KDa**


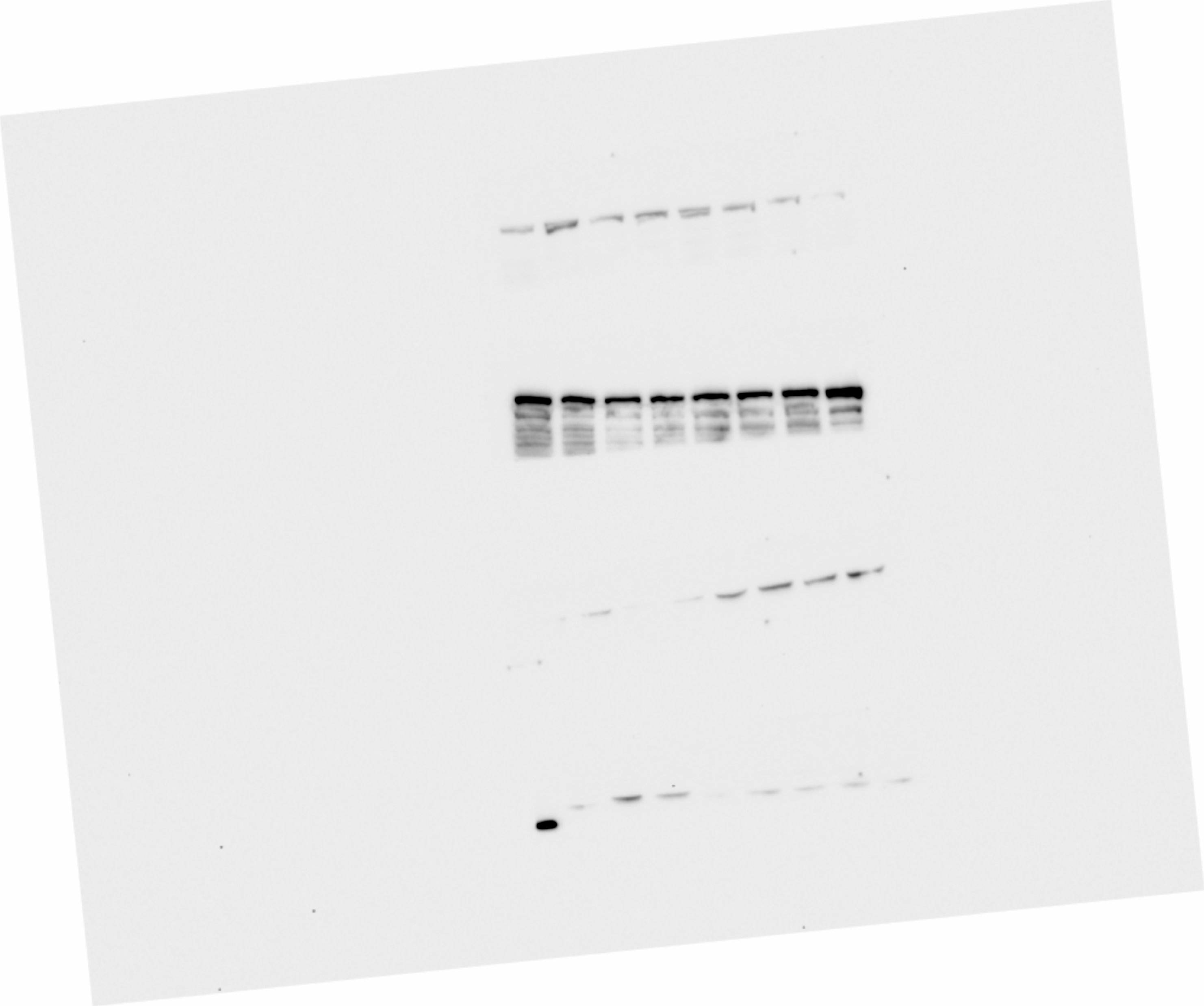


**289KDa**

**Fig. 2D**

**p-mTOR**


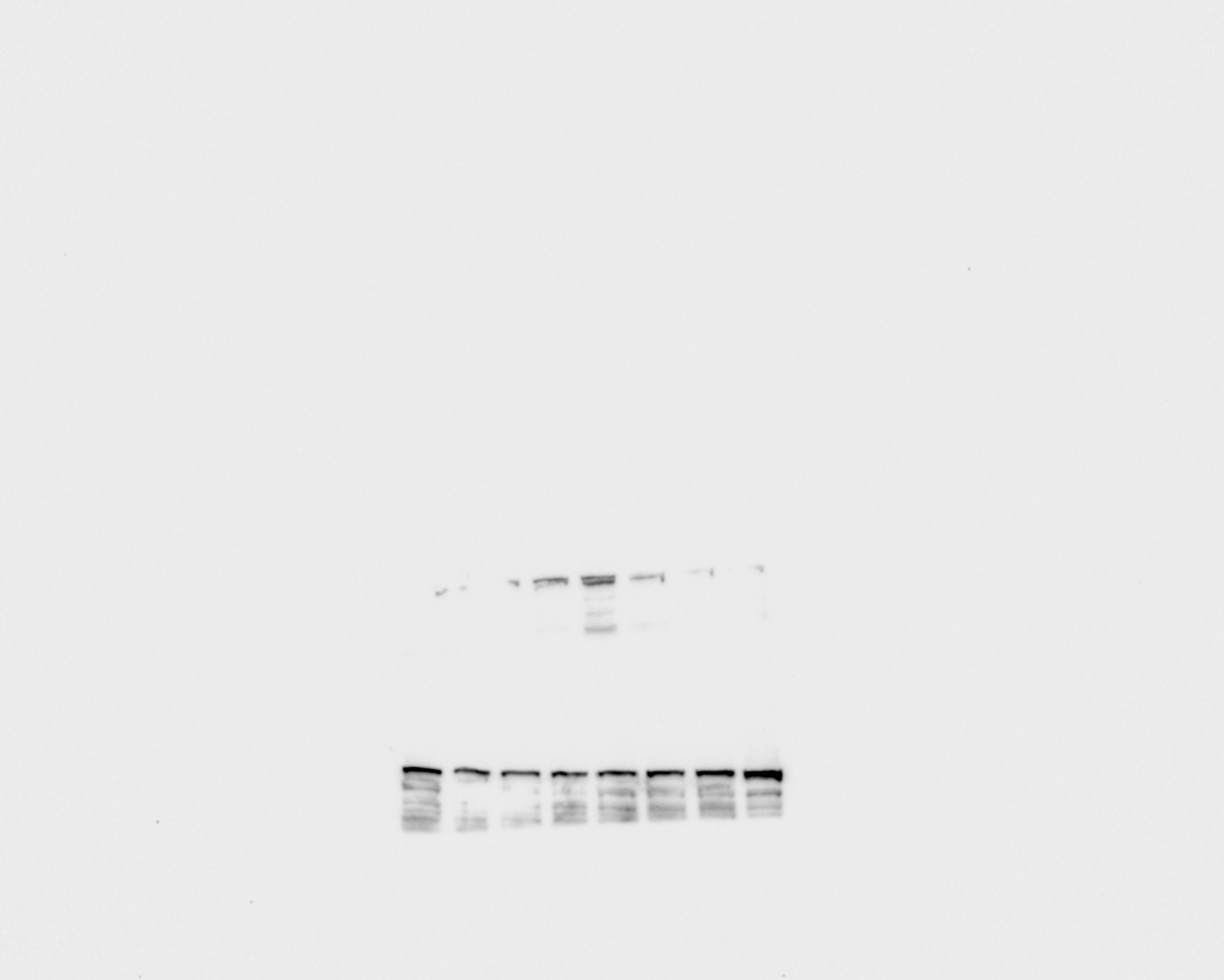


**289KDa**

**mTOR**

**p-p70S6K**


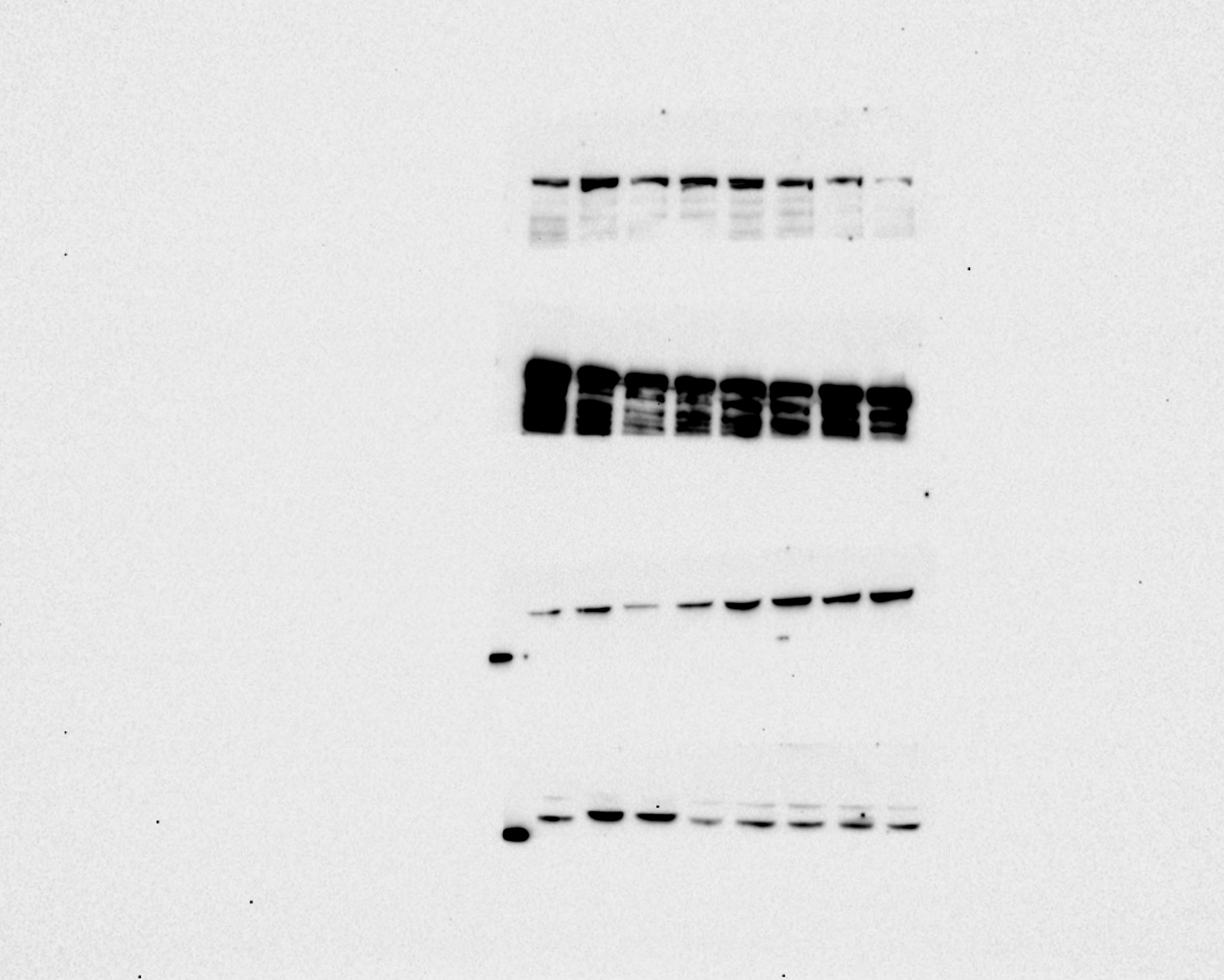


**70KDa**

**β-Actin**


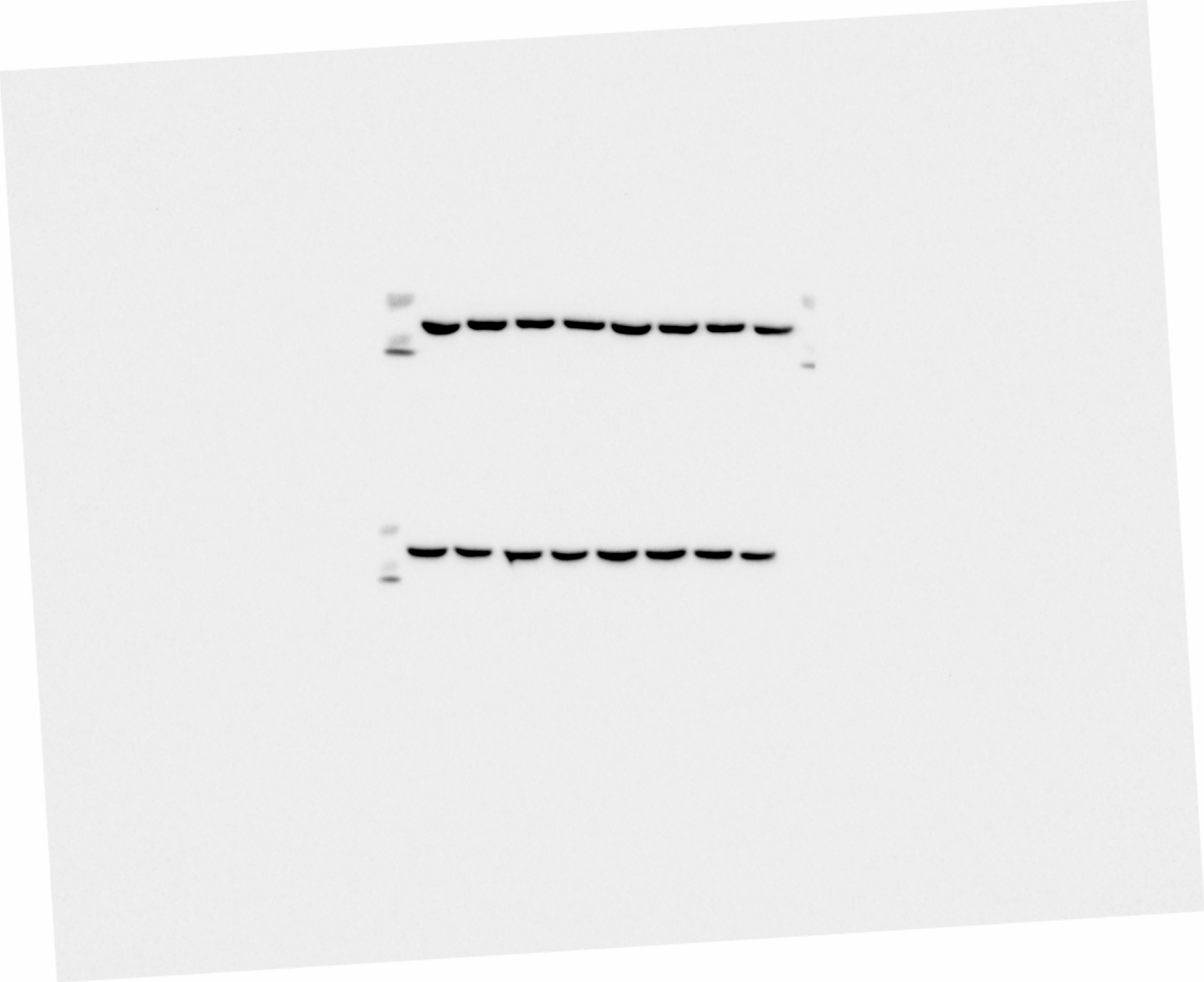


**50KDa**

**37KDa**

**25KDa**

**45KDa**

**The antiproliferative activity of most potent compounds at 24h**

**
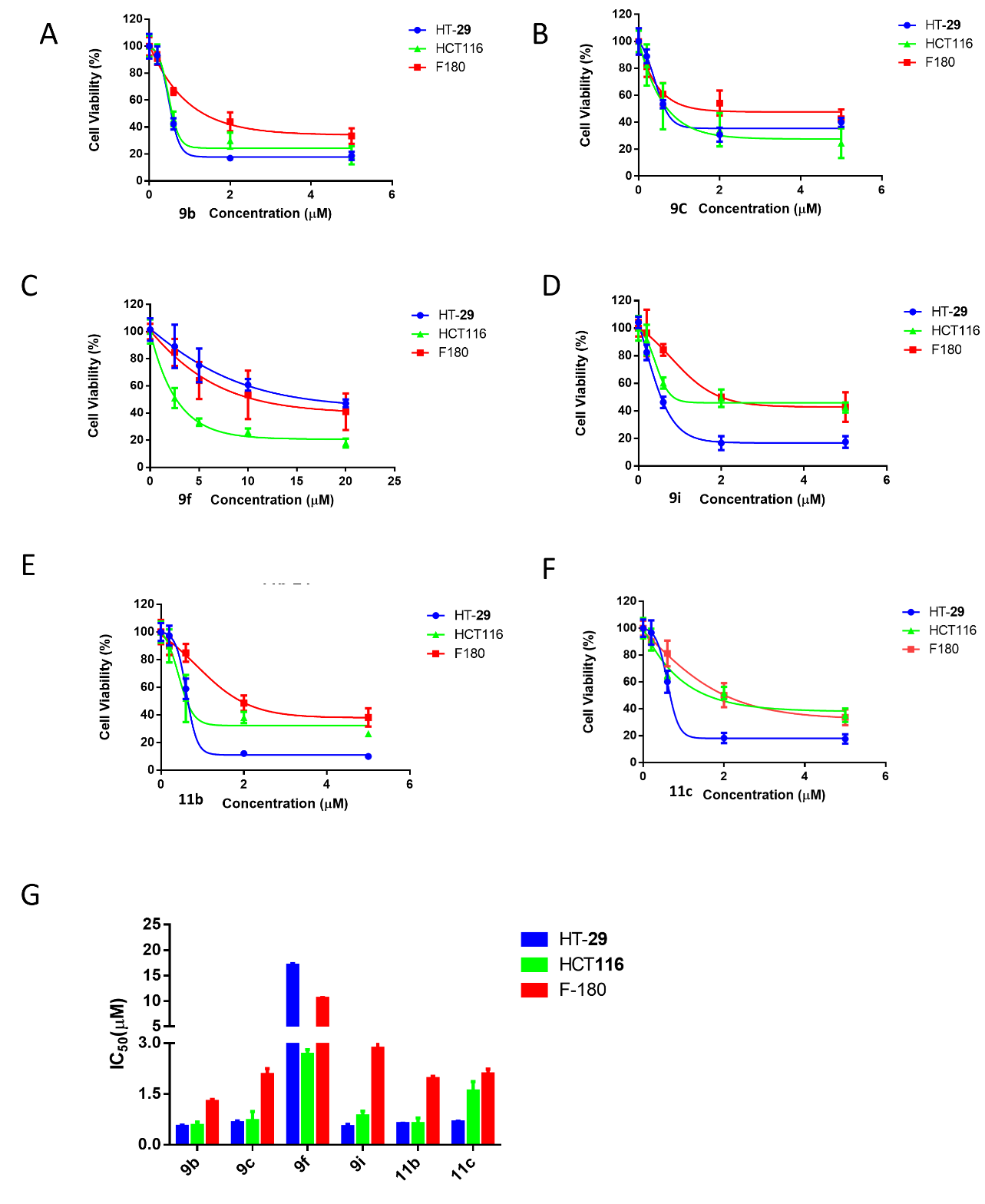
**

**The antiproliferative activity of most potent compounds at 48h**

**
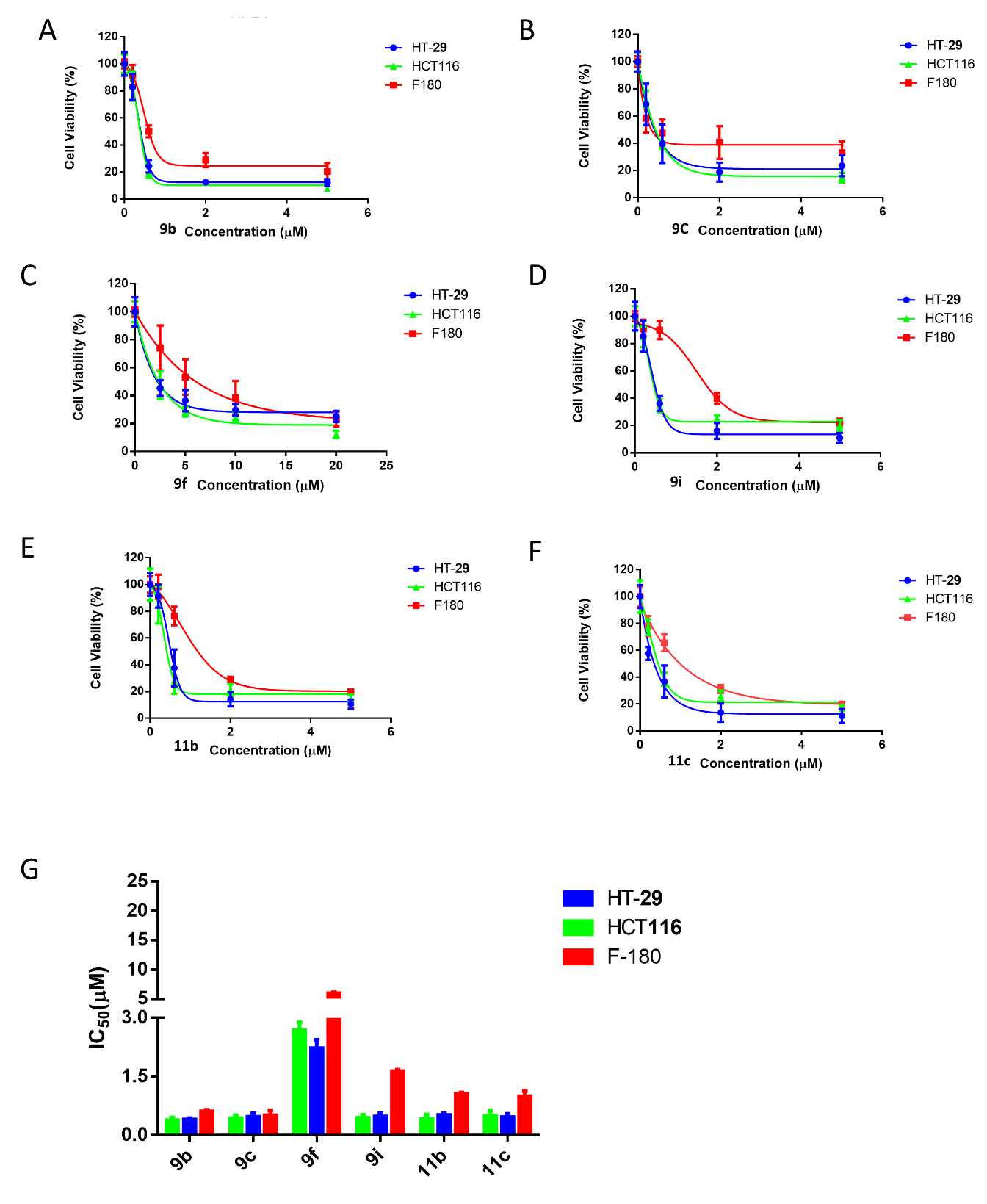
**

**Protein Quantitation Results**

The protein concentration was measured after 16h treatment using DC protein quantification kit.

**
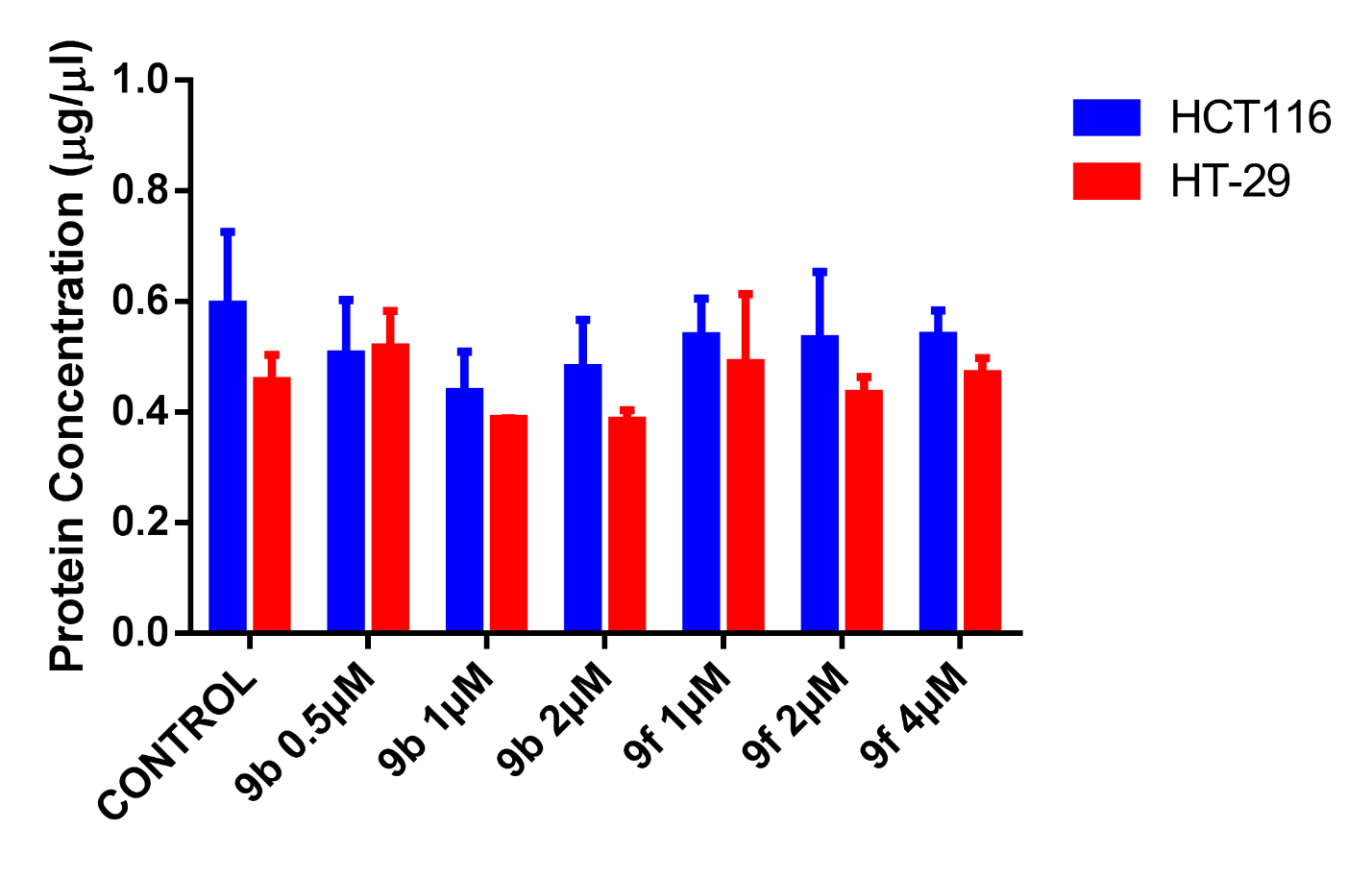
**
